# Optimization of Cation Exchange for the Separation of Actinium-225 from Radioactive Thorium, Radium-223 and Other Metals

**DOI:** 10.3390/molecules24101921

**Published:** 2019-05-18

**Authors:** Jonathan Fitzsimmons, Bryan Foley, Bryna Torre, Megan Wilken, Cathy S. Cutler, Leonard Mausner, Dmitri Medvedev

**Affiliations:** 1 Isotope Production Laboratory, Collider-Accelerator Division, Brookhaven National Laboratory, Upton, NY 11973, USA; ccutler@bnl.gov (C.S.C.); lmausner@bnl.gov (L.M.); dmedvede@bnl.gov (D.M.); 2 Department of Biology, Chemistry, and Geoscience, Fairmont State University, Fairmont, WV 26554, USA; bfoley@tamu.edu; 3 Department of Chemistry, University at Buffalo, the State University of New York, Buffalo, NY 14260, USA; brynator@buffalo.edu; 4 Department of Chemistry, Elizabeth City State University, Elizabeth City, NC 27909, USA; mmwilken412@students.ecsu.edu

**Keywords:** Lanthanum, Rhodium, AG50, MP50, fission products, Actinium-225, ^225^Ac, ^223^Ra, ^227^Th, thorium

## Abstract

Actinium-225 (^225^Ac) can be produced with a linear accelerator by proton irradiation of a thorium (Th) target, but the Th also underdoes fission and produces 400 other radioisotopes. No research exists on optimization of the cation step for the purification. The research herein examines the optimization of the cation exchange step for the purification of ^225^Ac. The following variables were tested: pH of load solution (1.5–4.6); rinse steps with various concentrations of HCl, HNO_3_, H_2_SO_4_, and combinations of HCl and HNO_3_; various thorium chelators to block retention; MP50 and AG50 resins; and retention of 20–45 elements with different rinse sequences. The research indicated that HCl removes more isotopes earlier than HNO_3_, but that some elements, such as barium and radium, could be eluted with ≥2.5 M HNO_3_. The optimal pH of the load solution was 1.5–2.0, and the optimized rinse sequence was five bed volumes (BV) of 1 M citric acid pH 2.0, 3 BV of water, 3 BV of 2 M HNO_3_, 6 BV of 2.5 M HNO_3_ and 20 BV of 6 M HNO_3_. The sequence recovered >90% of ^225^Ac with minimal ^223^Ra and thorium present.

## 1. Introduction

Actinium-225 (^225^Ac) and its daughter, bismuth-213 (^213^Bi), can be used to label molecules for targeted alpha therapy [1,2]. A targeted alpha particle typically has a range of 50–80 microns and results in a high amount of energy deposited to a small area, reducing the bystander damage [3]. The emitted alpha particle(s) can produce high radiation damage due to its high linear energy transfer, yet the short-range of the alpha particle results in less damage to the surrounding tissues [3]. In contrast, a targeted beta particle would have a range of 1–10 mm, and a larger amount of energy would be deposited to normal tissue [3].

The short half-life of ^213^Bi makes it a good therapeutic isotope for small targeting molecules such as peptides. However, the longer half-life of ^225^Ac makes the isotope more suited for large targeting molecules such as antibodies. The ^225^Ac can be used to make a generator to allow local production of ^213^Bi for clinical applications [4]. Some of the recent targeted alpha therapy studies have shown promise at treating cancer and potentially curing some cancers. A study with ^213^Bi DOTATOC shrank tumors when similar therapy with beta emitting isotopes failed [5]. In another study, ^225^Ac PSMA-617 treatment of patients with prostate cancer showed drastic reduction/elimination of metastatic tumors [6].

The recent success of targeted alpha therapy in a clinical setting has led some to estimate the demand of ^225^Ac to be greater than 50 Ci per year [7]. The current world supply of ^225^Ac is 1.2 Ci per year, of which 0.75 Ci is produced by Oak Ridge National Lab by a ^229^Th generator (decay chain: ^229^Th → ^225^Ra → ^225^Ac) [8]. The ability of alpha therapy to be widely used is dependent on the development of large scale production and purification of ^225^Ac. To address the shortage, the US Department of Energy formed a Tri-lab team with scientists from Los Alamos (LANL), Brookhaven (BNL) and Oak Ridge (ORNL) National Laboratories, with the goal of exploring alternative production routes of ^225^Ac. One production approach being examined is the proton irradiation of a ^232^Th target by the ^232^Th (p,x) ^225^Ac nuclear reaction. A 50- to 100-g thorium target could produce 1–5 Ci of ^225^Ac for a 10-day irradiation at Brookhaven Linac Isotope Producer (BLIP) [9].

During the proton irradiation of a ^232^Th target, the nuclear reaction Th(p,f) occurs, and the thorium undergoes fission. The fragmented mass distribution is typically a bimodal distribution with mass number peaks around 100 and 132, but a range of masses from 75–160 are produced [10]. As the proton energy increases (from 13 to 53 MeV), the fragmented mass distribution has a more symmetric fission and the peak is centered around 115 MeV, with a similar range of masses from 75–160 produced [10]. MCNPX calculations for proton-irradiated thorium targets at 199 MeV proton energies have been done, and 663 radioisotopes are produced at the end of the bombardment at greater than 1 mCi [11]. The number of isotopes produced at greater than 1 mCi decreases to 540 at 1 h, 329 at 1 day and 237 at 5 days. The ratio of fission products/^225^Ac is 12:1, and the radionuclides that are produced are from all the different groups in the periodic table [11]. During the proton irradiation of a thorium target, ^227^Ac and ^226^Ac are coproduced, and recently published literature discusses their impact on production [12].

The Tri-lab collaboration has examined the purification of ^225^Ac utilizing the following sequence: (1) HCl/anion exchange resin, (2) chelator/cation exchange resin, and (3) a nitric acid/DGA solvent extraction resin. [13,14]. This approach was a combination of literature methods for the purification of ^225^Ac from thorium [15,16,17]. Various approaches have examined the purification of ^225^Ac from thorium, and a cation exchange column with a chelating agent has been used [13,14,15,16,17,18]. The cation-exchange resin (AG50) contains a sulfonic acid functional group, which would retain the actinium species. Thorium would be chelated to the citric acid and would not be retained by the resin. The LANL study used a rinse step with 63.3 bed volumes (BV) of 0.5 M citric acid, then a rinse step of 13.3 BV of 1 M nitric acid, followed by an elution step utilizing 6 M nitric acid [13]. In the purification, the 1-M nitric acid rinse step did little to remove impurities, and rinsing the column with greater than 60 BV seems excessive and will increase the time of the cation step and produce a large volume of radioactive waste. None of the studies examined optimization of the cation column for the purification of ^225^Ac.

A proton-irradiated thorium target contains a large number of isotopes and is really complex, containing too many radioisotopes to use for preliminary experiments. To model the system, multi-element samples were used, and the optimized separation was developed in stages summarized in Figure 1. Cu, Pb, Zn, Co, Cr, Cd, Ni, Fe, Mn, Al, Ga, Ge, Sr, Be, Mg, Rb, Ba, Ce, Lu and Zr were chosen to study, as they represent elements across different groups of the periodic table. The group also represents environmental contaminates and target cladding materials that could appear in higher mass quantities than the fission fragments. The larger masses could have a higher impact on the retention of ^225^Ac. To better understand the elution behavior of different elements, larger studies with over 40 elements were performed (Figures S50–S154).

The studies herein determined the optimal elution profile for the cation resin step in the presence of a large number of fission products. To optimize the cation purification step results, the following research studies are reported: (1) variations of the pH of the load solution; (2) evaluation of rinse steps with various concentrations of HCl, HNO_3_ and H_2_SO_4_; (3) rinse steps with sequences of HCl and HNO_3_; and (4) comparative elution studies with two different cation resins (Bio-Rad MP50 vs). (5) Many of the studies were done comparing two different thorium chelating agents. (6) Once a suitable rinse sequence was established to remove a large number of impurities, studies were conducted to optimize the sequence to remove as much barium (Ba) as possible. Ba is chemically similar to radium. As the optimization of the cation step was being developed, more complicated samples were used. 

## 2. Results

**Figure 1:**
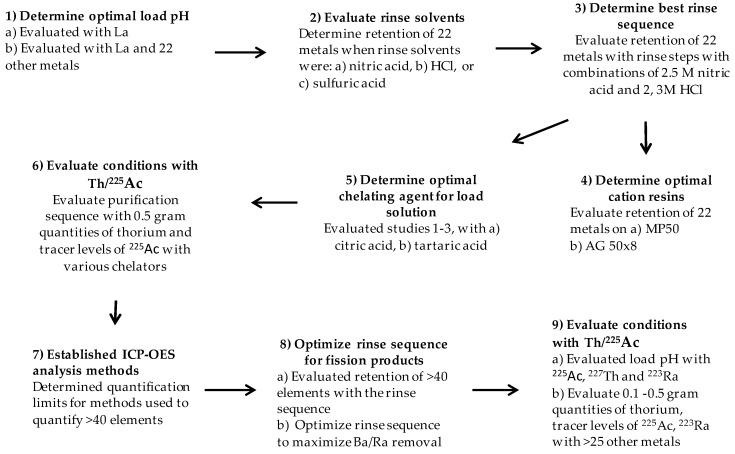
Flow chart illustrating the sequence of studies reported in the manuscript.

Initial research used lanthanum (La) as a surrogate for ^225^Ac, since both elements have a charge of +3 and have similar chemistry. The cation step of the separation was developed by evaluating the elution profiles of multiple elements. The approach utilized a cation exchange resin and a complexing agent (either citric or tartaric acid) to coordinate thorium. The studies are summarized in Figure 1.

### 2.1. Variations of the pH of the Load Solution for Retention of La and Evaluating Two Different Chelating Agents

Lanthanum was retained on the cation column at a pH range of 1.5–2.5. All subsequent studies were performed with a load solution at a pH of 2. Higher amounts of La were eluted in the load solutions of greater than pH 2.5. Initial studies were performed with a complexing agent utilizing lanthanum as a surrogate for ^225^Ac and spikes of Cu, Pb, Zn, Co, Cr, Cd, Ni, Fe, Mn, Al, Ga, Ge, Sr, Be, Mg, Rb, Ba, Ce, Lu and Zr. Separate studies were performed for citric acid and tartaric acid (the data sets are in the Supplementary Materials), and the results are briefly summarized as follows (Figures S1–S16).

### 2.2. Evaluation of Rinse Steps with Various Concentrations of HCl, HNO_3_ and H_2_SO_4_

Evaluation of rinse steps with various concentrations of HCl and/or nitric acid indicated the following. Cu, Pb, Zn, Co, Cr, Cd, Ni, Fe, Mn, Al, Ga, Sr, Be, Mg, Rb, Ba, Ce, Lu and La were all retained on the cation column from the load solution. Ge and Zr had minimal retention and ~30% of Cr was eluted in the load solution (Figures S6–S11). Rinse solutions with HCl eluted the elements at lower molarities than with similar concentrations of HNO_3_ and, in general, higher amounts of the elements were eluted in each HCl rinse step. For some elements such as Cu, Zn, Co, Cd, Ni, Fe, Al Ga, Be, Mg and Rb, elution with sulfuric acid was similar or better than eluting with HCl. La and Ce began to elute in 3 M sulfuric acid, whereas in 3 M HCl Ce and La were not eluted with 3 BV. Lanthanum will begin to elute from the cation column when 5 BV of 3 M HCl are used. As a result, rinse steps with 3 M HCl should be limited to 3 BV. Over 50% of lanthanum was eluted when 3.5 M HCl or 3.5 M nitric acid are used to rinse the column. Cerium (Ce) and La behave the same in most studies presented herein, and their separation is covered in other published studies [13,14]. Lu can be eluted with 3 M HCl and Ba can be eluted starting with 2.5 and 3 M HNO_3_. This set of data indicates that a combination of HCl and HNO_3_ should be used to elute the elements.

### 2.3. Rinses Steps with Sequences of HCl and HNO_3_

The results obtained in the mixed metal elution profiles indicate two rinse sequences were proposed for the cation separation step. One rinse sequence utilized HNO_3_- and water prior to rinsing with HCl in an attempt to remove barium from the solution (Figures S43–S47). A water rinse continued to elute the metals released in the HNO_3_ rinse. The rinse sequence failed at removing the majority of barium in one rinse step. Instead, 10%–20% of barium was eluted in each rinse step after 2.5 M HNO_3_. The second rinse sequence involved rinses with HCl before HNO_3_ (Figures S34–S424). Rinsing the cation column with 2 M HCl, 3 M HCl water and 2.5 M nitric acid resulted in eluting more metals early at higher concentrations than when the elution profile was 2.5 M nitric acid, water and 3 M HCl. A bulk of the lutetium was eluted in the 3-M HCl rinse step. Barium was retained during HCl rinse steps, but 77% was eluted during the 2.5-M nitric acid rinse step. When the metals retained on the cation column were rinsed with 3 BV of: 2 M HCl, 3 M HCl, water, 2.5 M nitric acid and water, then eluted with 20 BV of Conc. HCl, no lanthanum was eluted. Therefore, eluting lanthanum from the column can be achieved in 3.5–8 M HCl with multiple BV of the acid.

### 2.4. Comparative Elution Studies with Two Different Cation Resins

A comparison of Bio-Rad AG-50 and MP-50 resin with the rinse sequence indicated the two resins had different retention of some of the metals (Figures S26–S33). Many elements had similar elution profiles with the two different resins. No lanthanum was eluted from a Bio-Rad MP-50 resin when the rinse sequence was 2 M HCl, 3 M HCl, water, 2.5 M nitric acid, water and 8 M HCl. The same rinse sequence was used with Bio-Rad AG-50 resin and some lanthanum (40%) was eluted. Ba, Sr and Lu all eluted at >80% before the 8-M HCl step from an AG-50 resin with tartaric acid (Figures S26–S33), and similar studies with MP-50 resin eluted <10% of Ba, Sr and Lu before the 8-M HCl step (Figures S26–S33). Rinsing the MP-50 resin with 8 M HCl eluted 96% of the Lu, but in the AG-50 studies over 55% eluted in the 3-M HCl steps, and no La was eluted in the 8-M HCl step. 

### 2.5. Elution Profiles of Rh and La on a Cation Column

Elution profiles of Rh and La were generated with the following rinse sequences: (1) 3 BV of 2 M HCl, 3 BV of 3 M HCl, 3 BV of 18MΩ water, 3 BV of 2.5 M HNO_3_, 3 BV of 18 MΩ water and 10 BV of 10 M HNO_3_ (Figures S48–S49); and (2) 3 BV of 2 M HCl, 3BV of 3 M HCl, 3 BV of 18 MΩ water, 3 BV of 2.5 M HNO_3_, 10 BV of 3.5 M HCl, 10 BV of 4 M HCl, 10 BV of 5 M HNO_3_ and 10 BV of 6 M HNO_3_ (Figures S48–S49). Sequence (1) eluted 57.9% of the La in the 10-M nitric acid and no La was eluted in the previous steps. In sequence (2), La eluted in 4 M HCl (26.8%), 5 M HNO_3_ (43.7) and 6 M HNO_3_ (17.6), while no La was eluted in previous steps. 

### 2.6. Optimizing the Elution Profile

Elution profiles of all the elements examined in studies 1–3 are shown in the Supplementary Figures S50–S154). Briefly, the profiles are summarized as follows. In studies 1 and 2, many of the elements eluted in similar rinse steps. For both studies the following elements were primarily eluted in the citric acid and water rinse steps: Ga, B, Bi, Re and Sc. For both studies the following elements were primarily eluted in the 2- and 3-M HCl rinse steps: Ag, Co (evaporated in nitric acid), Ni, Cu, Zn, Mn Li, Rb, Be Mg, In, Rb and Zn. In studies 1 and 2 the following elements were eluted in both the load and HCl solutions: Al, Cr, Tl, Ge Rh, U and Se. Sr was eluted in the HCl and 2.5-M nitric acid rinse steps in both experiments. Fe, Pb and Ru eluted in the HCl and 6-M nitric acid steps in study 1, while Fe was present in the load and HCl rinse steps in study 2 but was not present in the 6-M nitric acid. Pb had a similar elution profile in study 2 and showed up in the HCl rinse steps. Ru eluted in the HCl and 2.5-M nitric acid rinse steps in study 2 and was not present in the 6-M nitric acid rinse steps.

In study 1, the metals were evaporation from either HCl or nitric acid, dissolved in 1 M citric acid pH 2 and the cation column performed. For many elements the elution profile was similar. When Ga was evaporated in HCl only 57% was recovered, and it was eluted in the rinse step, but Ga was not present in the 6-M nitric acid solutions. However, when Ga was evaporated from nitric acid 37.3% of the Ga, it was present in the rinse step and 37.9% was present in the 6-M nitric acid step. Mo was eluted in the load solution with 73% present when the sample was evaporated from HCl compared with only 25% eluted when the sample was evaporated from nitric acid. The load solution contained 38% of the W when the sample was evaporated from HCl, but no W was detected when the sample was evaporated from nitric acid. Similar results were observed with Ti, where over 40% was eluted in the load solution when evaporated from HCl. Less than 10% of Ti was in the load solution when the sample was evaporated from nitric acid. In the study, 27% of As was eluted in the 6-M nitric acid solution when the sample was evaporated from nitric acid. When the sample was evaporated in HCl, and the separation performed, As was below the detection limit in the 6-M nitric acid step. 

Lanthanides eluted over multiple rinse steps in studies 1 and 2, and Dy, Er, Eu, Gd, Ho, Lu, Nd, Sm, Tb, Tm, Yb and Y were initially eluted in 2 or 3 M HCl, and in the 2.5- and 6-M nitric acid steps (Figures S48–S154). Many of these elements were eluted in the 6-M nitric acid step, but in very low percentages. La, Ce and Pr had similar elution profiles in study 1 where all three metals began to elute in the second 3-M HCl elution step. The metals gradually eluted over the 2.5- and 6-M nitric acid steps. In study 2, La, Ce and Pr all had similar elution profile with the 6-M nitric acid step, eluting many of the metals. The step contained 60%–85% of the eluted La (Figures S48–S49), 79% of the eluted Ce and 69% of the eluted Pr and 55% of the eluted Nd; all other lanthanides were present in less than 50%. Ba is a surrogate for Ra, as the elements are in the same group and have a charge of +2. Both elements are produced in a proton-irradiated Th target as ^140^Ba and in multiple radioactive Ra nuclides. Removal of these elements is important for the purification of ^225^Ac. In study 1, Ba initially eluted in the first 3-M HCl step, peaked in the first 2.5-M nitric acid step and finished eluting in the 6-M nitric acid steps. In study 2, the elution of Ba was ideal for the separation, where over 90% of the Ba eluted in the 2.5-M nitric acid rinse steps and none was present in the 6-M nitric acid (Figures S102–S154).

### 2.7. Th/^225^Ac Studies

#### 2.7.1. Th/^225^Ac Studies with Different Chelating Agents

Dissolving thorium in citric acid provided some interesting insights, as solubility issues have been problematic. When 0.5 g of thorium nitrate was dissolved in citric acid solutions with volumes of 25 mL, the solution appeared to form a white solution between pH 1.3–1.6. At pH 1.1–1.2 and 2–3.36 the solution was clear. This is consistent with the literature studies where solutions of thorium citrate formed a white precipitate Th(Cit)_2_ species [19]. Performing the cation column separation with a 3:1 ratio of citric acid/thorium at pH 2 resulted in precipitation of thorium on the column (white bands appeared) as well as some precipitation in the collection vial (BNL). The solution was clear when it was loaded onto the column. This indicates that the formation of the insoluble Th(Cit)_2_ species may be kinetically slow. No precipitation was observed with a 5:1 ratio of citric acid/thorium with a ^225^Ac spiked solution and ~0.5 g of thorium. A ratio of 5:1 of citric acid/thorium provided a solution where ^225^Ac could be retained while Th was eluted through the column. Studies conducted at a 10:1 ratio of citric acid/thorium using the elution profile: 5 BV of 1 M citric acid pH 2.0, 3 BV of water, 3 BV of 2 M HNO_3_, 2 × 3 BV of 2.5 M HNO_3_ and 2 × 10 BV of 6 M HNO_3_. Analysis of the 6-M nitric acid solution indicated quantitative recovery of ^225^Ac with thorium below quantification limits by inductively coupled plasma—optical emission spectrometry (ICP-OES) (0.1 ppm). Studies with tartaric acid produced similar results with white precipitation of the thorium species. Attempts to solubilize the thorium species were more difficult, and in some solutions the thorium precipitate remained insoluble until pH 3.0. The thorium tartrate precipitate seemed to form a larger and harder precipitate compared to precipitates observed with citric acid. The insoluble thorium tartrate species was much more difficult to solubilize, and all subsequent studies were performed with citric acid.

#### 2.7.2. Variations of the pH of the Load Solution for Retention of ^225^Ac, ^227^Th and ^223^Ra with Citric Acid

**Figure 2:**
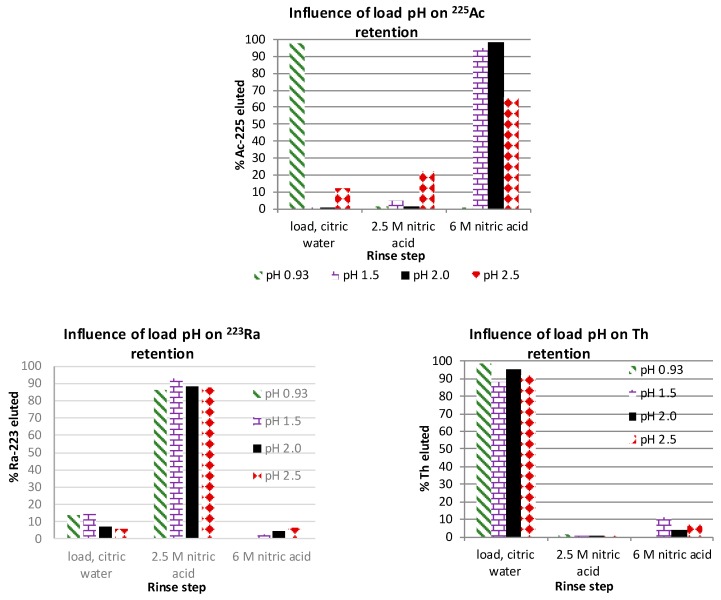
Influence of load pH on retention of ^225^Ac, ^223^Ra and ^227^Th. Load solution = 0; rinse sequence: 3 mL of 1 M citric acid pH 2, water, 2 × 3 mL of water and 2 × 10 mL of 6 M nitric acid. 95%–98% of ^225^Ac was recovered in the 6-M nitric acid rinse when the load pH was 1.5 or 2.0. When the pH was 2.5, the recovery of ^225^Ac was reduced to 66%. pH had minimal influence on the retention of ^227^Th, and 86%–92% of ^223^Ra was eluted in the 2.5-M nitric acid rinse step.

**Figure 3:**
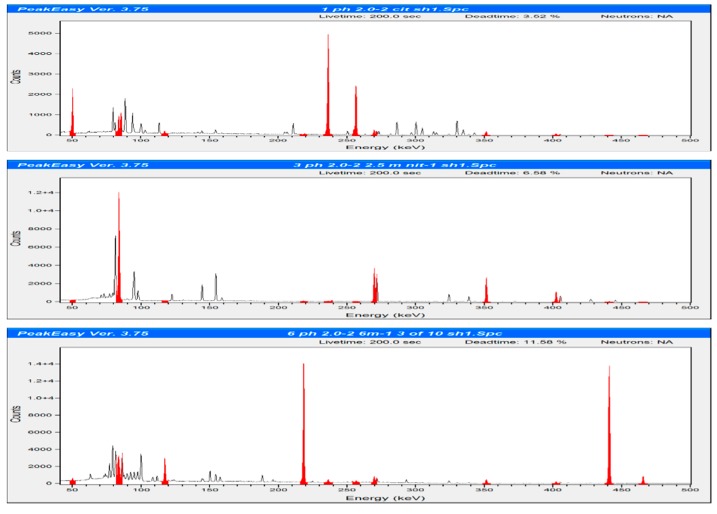
Gamma spectroscopy spectrums of the eluted: load (**top**), 2.5-M nitric acid (**middle**) and 6-M nitric acid (**bottom**) solutions from the cation study with 1 M citric acid at pH 2.0. PeakEasy version 3.75 was used to plot the spectra from 40.9 to 500.5 keV, and the following regions of interest were highlighted: ^227^Th and daughters 50 keV (^227^Th), 83 keV (^223^Ra), 118 keV (^209^Tl), 218 keV (^221^Fr), 236 keV (^227^Th), 256 keV (^227^Th), 269 keV (^223^Ra), 271 (^229^Rn), 351 keV (^211^Bi), 402 keV (^219^Rn), 440 keV (^213^Bi) and 465 keV (^209^Tl).

The influence of the pH (pH = 0.93, 1.5, 2.0 and 2.5) of the load solution (1 M citric acid) was investigated by looking at the retention of ^225^Ac, ^223^Ra and ^227^Th across the rinse sequence: 3 mL of 1 M citric acid pH 2.0, 3 mL of water, 2 × 3 mL of 2.5 M nitric acid and 2 × 10 mL of 6 M nitric acid (Figure 2 and Figure 3). At pH 0.93 ^225^, Ac and ^227^Th were not retained on the cation resin with ~98% and 99% eluting in the load, citric acid and water rinse steps. Only 13.6% of ^223^Ra was eluted in the same rinse steps at pH 0.93. ^225^Ac was eluted in the 6-M nitric acid rinse at 95% and 98% when the load solution was at pH 1.5 and 2.0. When the load solution pH was 2.5, the elution of ^225^Ac in the 6-M nitric acid rinse step was reduced to 66% and 11.8% of ^225^Ac was eluted in the load, citric and water rinse steps, 22% of the ^225^Ac was in the 2.5-M nitric acid rinse step. ^223^Ra had similar elution profiles at the different pH values tested and 86%–93% was eluted in the 2.5-M nitric acid rinse step. The elution of Th was similar across the pH values tested with 88%–99% eluted in the load, citric acid and water rinse steps.

#### 2.7.3. Th/^225^Ac Studies and Other Metals with the Optimized Rinse Sequence

The following rinse sequences were evaluated for retention of ^227^Th, ^225^Ac, ^223^Ra and over 20 other metals. Rinse Sequence (1): 5 BV of 1 M citric acid pH 2.0, 3 BV of water, 3 BV of 2 M HNO_3_, 2 × 3 BV of 2.5 M HNO_3_ and 2 × 10 BV of 6 M HNO_3_ (Table 1 summarizes the elution of different elements). Rinse Sequence (2): 5 BV of 1 M citric acid pH 2.0, 3 BV of water, 3 BV of 2 M HCl, 3 BV of 3 M HCl, 3 BV of water, 2 × 3 BV of 2.5 M HNO_3_ and 2 × 10 BV of 6 M HNO_3_ (Table 2 summarizes the elution of different elements). Rinse Sequence (3): 5 BV of 1 M citric acid pH 2.0, 3 BV of water, 2 × 3 BV of 2.5 M HCl, 3 BV of water, 2 × 3 BV of 2.5 M HNO_3_ and 2 × 10 BV of 6 M HNO_3_ (Table S1 summarizes the elution of different elements).

## 3. Discussion

^225^Ac can be produced with a linear accelerator by proton irradiation of a thorium (Th) target [20]. However, during the irradiation the thorium also undergoes fission and produces 400 other radioisotopes and other stable elements. The published three-step purification process used was: (1) MP1/HCl; (2) cation exchange/complexing agent; and (3) B-DGA resin/nitric acid [13,14]. The studies herein were designed to optimize the conditions needed to elute the cation column step for the purification of ^225^Ac from thorium. The MP1/HCl step in the current process was designed to remove many of the fission products, but does not remove ^225^Ac or thorium.

Various conditions were evaluated and optimized for the cation purification step in the ^225^Ac purification process. Minor differences were observed in the metal elution profile when citric or tartaric acids were used, and the lanthanum elution profile was similar, using either citric or tartaric acid as the complexing agent. However, studies with thorium indicated citric acid was much easier to use then tartaric acid. Bio-Rad AG-50X8 (100–200 mesh) resin was better at eluting La than the MP-50 version of the resin. The influence of the load pH on retention of ^225^Ac, ^223^Ra and ^227^Th was tested with the following rinse sequence: 3 mL of 1 M citric acid pH 2, water, 2 × 3 mL of 2.5 M HNO_3_ and 2 × 10 mL of 6 M nitric acid. When the load pH was 1.5 or 2.0 there was a 95–98% recovery of ^225^Ac in the 6-M nitric acid rinse step. When the pH was 2.5, the recovery of ^225^Ac was reduced to 66%. pH had minimal influence on the retention of ^227^Th, and 86–92% of ^223^Ra was eluted in the 2.5-M nitric acid rinse step. To achieve a desired product quality, the United States Food and Drug Administration (FDA) requires critical process parameters to be identified, then monitored or controlled [21]. During the purification of ^225^Ac, the pH of the load solution for the cation purification step would influence the ^225^Ac yield and needs to be monitored with the optimal pH between 1.5 to 2.

The three rinse sequences reported in these studies were evaluated to determine if one rinse sequence could replace the MP1/HCl or the BDGA step in the current process to purify ^225^Ac from thorium. A rinse sequence of 5 mL of 1 M citric acid pH 2, 3 mL of water, 3 mL of 2 M HCl, 3 mL of 3 M HCl, 3 mL of water, 2 × 3 mL of 2.5 M nitric acid and 2 × 10 mL of 6 M nitric acid was evaluated for the purification of ^225^Ac from ^223^Ra, thorium and other metals (Table 2). The sequence eluted 90% of the ^225^Ac in the 6-M nitric acid step, with 9.5% of the ^225^Ac in the 2.5-M nitric acid rinse step. Only 44% of the ^223^Ra was eluted in the 2.5-M nitric acid rinse step, with 48.2% eluted in the first 6-M nitric acid rinse. The load, citric and first water rinse steps contained 85% of the ^227^Th. The elution of lanthanides was interesting, as lighter lanthanides (La, Ce and Pr) were retained longer and the peak of their elution was in 6-M or 2.5-M nitric acid fractions. The elution peak for many of the other metals was in the 2-M or 3-M HCl. This sequence was the best sequence to elute the lanthanides and other metals studied. However, almost half of the ^223^Ra was coeluted with the ^225^Ac in the 6-M nitric acid rinse step. A rinse sequence of 5 mL of 1 M citric acid pH 2, 3 mL of water, 2 × 3 mL of 2.5 M HCl, 3 mL of water, 2 × 3 mL of 2.5 M nitric acid and 2 × 10 mL of 6 M nitric acid was evaluated for the purification of ^225^Ac from ^223^Ra thorium and other metals (Table S1). The 6-M nitric acid step eluted 77.4% of the ^225^Ac, and 18.7% of the ^225^Ac was eluted in the 2.5-M nitric acid rinse step. During the 2.5 M nitric acid rinse step 76.3% of the ^223^Ra was eluted, and 8.4% was eluted in the 6-M nitric acid step. The load, citric acid and first water rinse steps eluted 92.3% of the ^227^Th. The elution peak of the lanthanides was in either the second 2.5-M nitric or first 6-M nitric acid rinse steps. The elution peak for the other metals was in the 2.5-M HCl step. The low recovery of the ^225^Ac in the 6-M nitric acid step was the worst of the three rinse sequences. A rinse sequence of 5 mL of 1 M citric acid pH 2, 3 mL of water, 3 mL of 2 M nitric acid, 2 × 3 mL of 2.5 M nitric acid and 2 × 10 mL of 6 M nitric acid was evaluated for the purification of ^225^Ac from ^223^Ra thorium and other metals (Table 1). The 6-M nitric acid rinse step eluted 93.3% of the ^225^Ac, the 2.5-M nitric acid rinse step eluted 97.3% of the ^223^Ra and the load, citric acid and first water rinse steps eluted 93.5% of the ^227^Th. The rinse sequence was the best at separating the three isotopes. The elution of most of the lanthanides peaked in the 6-M nitric acid rinse, and the elution peak of many of the other metals was in the 2.5-M nitric acid rinse step. All three rinse sequences can remove a lot of elements prior to the elution of ^225^Ac in 6 M nitric, and could potentially replace the MP1/HCl step in the purification process. However, none of the rinse sequences can separate lanthanides from Ac, so a second purification step such as B-DGA resin would be needed for the purification.

### Impact on the Separation Being Developed

The rinse sequence of 5 BV of 1 M citric acid pH 2, 3 BV of water, 3 BV of 2 M HNO_3_, 6 BV of 2.5 M HNO_3_ and 20 BV of 6 M HNO_3_ was incorporated into the cation resin step for processing 10–20 g of irradiated thorium production targets at ORNL [22]. Thorium was not present in the 6-M nitric acid step, but greater than 95% of ^225^Ac was eluted in the 6-M nitric acid step. The overall recovery of ^225^Ac for the total process was greater than 90%, with no thorium present in the purified ^225^Ac. The 2.5-M nitric acid rinse step removed >85% of the radioactive Ba/Ra during the processing of the thorium target. The 2.5-M nitric acid rinse step can be used to elute Ba/Ra. The addition of the rinse step provides an easier approach to capture the Ba/Ra fraction compared with other published methods [17,18,23]. In a processed target, this fraction would contain ^140^Ba, ^223^Ra, ^225^Ra and ^224^Ra. The fraction could be used in a generator system to produce pure ^225^Ac, lead isotopes (^212^Pb, ^211^Pb) and ^140^La. Conducting the separation with different personnel and at two national labs with similar results indicates that the separation of thorium from ^225^Ac had excellent reproducibility.

## 4. Materials and Methods

MP50 and AG50 × 8 (100–200 mesh) resin and disposable columns were purchased from Bio-Rad (Hercules, CA, USA), and all materials were purchased from Fisher Scientific (Hampton, NH, USA). All acid solutions were prepared from either reagent or trace metal grade solutions and diluted with 18 MΩ water (Millipore, Burlington, MA, USA). Metal solutions were prepared from ICP Standards purchased from SPEX Certiprep (Metuchen, NJ, USA). La and multi-element solutions were prepared using 50 µg of the metal(s) from the ICP standards, and solutions were evaporated to dryness and dissolved in 0.1 M HCl prior to use in the separations. For initial multi-element studies the following elements were used: Al, Ba, Be, Ca, Cd, Ce, Co, Cs, Cr, Cu, Fe, Ga, Ge, La, Lu, Mg, Mn, Nb, Ni, Pb, Rb, Sr, Zn and Zr. ^225^Ac was received from Oak Ridge National Laboratory as a dried sample and was dissolved in 1 mL of 0.1 M HCl prior to use. 1 mCi (37 MBq) of accelerator-produced ^225^Ac was received from Oak Ridge National Laboratory, and 1–20 µCi (37–740 kBq) were used in ^225^Ac tracer experiments. ^223^Ra and ^227^Th were present in the sample from the decay of ^227^Ac, and the isotopes were used to evaluate the elution profiles of thorium and radium.

### 4.1. pH Studies

La only: The La solution was added to 3 mL of 1 M citric acid, the pH was adjusted to 1.65 with concentrated ammonium hydroxide and loaded onto an AG50 resin (1 mL). Following the load elution, the resin was rinsed with 1 BV of citric acid at pH 1.65. The resin was subsequently rinsed with 1 BV of water, 1.5 M HCl, 4 M HCl and 6–8 M HCl, with each collected into separate test tubes, and La was quantified by ICP-OES. The preceding procedure was repeated for pH values of 2, 2.5, 3, 3.4 and 4.6, and the experiment was repeated with tartaric acid.

Multi-element: The multi-element solution was prepared and added to 15 mL of 1 M citric acid; the pH was adjusted with concentrated ammonium hydroxide to 1.5 and loaded onto an AG50 resin. The rinse steps mirrored those used in the La-only test, and the eluted elements were quantified by ICP-OES. The procedure was repeated at pH 1.97 and 2.48, and the studies were repeated with tartaric acid.

^225^Ac, Th, ^227^Th, and ^223^Ra: Thorium nitrate (25 g) was dissolved in 25 mL of water and 0.25 mL was added to a vial. A tracer level of accelerator-produced ^225^Ac with ^223^Ra and ^227^Th was added to the vial. The pH of sample was adjusted with 4 M nitric acid to a pH of either 0.93, 1.5, 2.0 or 2.5. The solution was loaded onto a 1-mL AG50 × 8 column. The column was rinsed with 3 mL of 1 M citric acid pH 2.0, water, 2 × 3 mL of 2.5 M nitric acid, and 6 M nitric acid. Gamma spectroscopy was used to quantify the amount of ^225^Ac, ^223^Ra and ^227^Th in the different fractions.

### 4.2. Evaluation of Rinse Solutions: HCl, HNO_3_ and H_2_SO_4_

Mixed metal solution: The multi-element solution was prepared and added to 15 mL of 1 M citric acid; the pH was adjusted to 2 with concentrated ammonium hydroxide; the solution was loaded onto an AG50 resin and rinsed with 3 BV of citric acid. The column was further rinsed with 3 BV of water, 1.5 M HCl, 2 M HCl, 2.5 M HCl, 3 M HCl and 5 BV of 8 M HCl. All samples were collected, and ICP-OES was used to determine the concentration of the metals in each rinse solution. The experiment was then repeated using the same concentrations of HNO_3_ and H_2_SO_4_. The HCl and Nitric acid studies were repeated with tartaric acid.

### 4.3. Evaluation of Rinse Sequences with Combinations of HCl and Nitric Acid and MP50 or AG50 × 8 Resin

Approximately 50 µg of La and over 20 other metals were taken up in 0.1 M HCl and added to 15 mL of 1 M citric acid at a pH of 2.0. The solution was loaded onto an AG50 resin and rinsed with 3 BV of citric acid at the same pH as the load solution. The column was further rinsed with 3 BV of water, 2.5 M HNO_3_, water, 3 M HCl and 8 M HCl. The samples were collected and ICP-OES was used to determine the concentration of the metals in each rinse. The experiment was then repeated with the following rinse scheme: 3 BV of water, 2 M HCl, 3 M HCl, water, 2.5 M HNO_3_, water and 8 M HCl. Both experiments were repeated with tartaric acid. The study with the rinse sequence of 2 M HCl, 3 M HCl, water, 2.5 M HNO_3_, water and 8 M HCl was repeated with tartaric acid and MP50 resin.

### 4.4. Retention of Rh and La on a Cation Column

To a vial was added 50 mL of 1 M citric acid at pH 2 and 50 μg of La; the pH was adjusted to 2 with concentrated ammonium hydroxide and loaded onto a 1-mL AG 50 × 8 column. The column was rinsed with 3 BV of 2 M HCl, 3 BV of 3 M HCl, 3 BV of 18 MΩ water, 3 BV of 2.5 M HNO_3_, 3 BV of 18 MΩ water, and 10 BV of 10 M HNO_3_. The study was repeated with 50 μg Rh and La and the elution profile was changed to 3 BV of 2 M HCl, 3 BV of 3 M HCl, 3 BV of 18 MΩ water, 3BV of 2.5 M HNO_3_, 10 BV of 3.5 M HCl, 10 BV of 4 M HCl, 10 BV of 5 M HNO_3_ and 5 BV of HNO_3_.

### 4.5. Mixed Metal Separation Method (26 Elements)

#### 4.5.1. Study 1: Evaluating 2.5-M Nitric Acid Rinse Step to Remove Ba

To a vial was added 1 mL of PE Pure Plus atomic spectroscopy calibration standards for multi-element ICP-MS standards 2, 3 and 5. The solution contained Al, As, Ba, Be, Bi, Ca, Cd, Co, Cr, Cs, Cu, Fe, Ga, In, K, Li, Mg, Mn, Ni, Pb, Rb, Se, Na, Ag, Sr, Tl, V, U, Zn, B, Ge, Mo, Nb, P, Re, S, Si, Ta, Ti, W, Zr, Ce, Dy, Er, Eu, Gd, Ho, La, Lu, Nd, Pr, Sm, Sc, Tb, Th, Tm, Y, Yb and Ru, and the solution was evaporated to dryness. The residue was taken into 25 mL of concentrated nitric acid and dried. Twenty mL of a 1-M citric acid pH 2 solution was added and the sample was dissolved and added to a 1-mL AG50 × 8 column. The following elution profile was used: 5 BV of 1 M Citric acid, 3 BV of 2 M HCl, 2 × 3 BV of 3 M HCl, 3 BV of water, 4 × 3 BV of 2.5 M HNO_3_ and 2 × 10 BV of 6 M NO_3_. The elution profile of each element was determined by ICP-OES. The process was repeated, and the initial dried residue was taken up in 25 mL of concentrated HCl, dried, and the procedure was followed.

#### 4.5.2. Study 2: Optimized Elution Procedure

To a vial was added 1 mL of periodic table mix 1, 2 and 3 (Sigma Aldrich, St. Louis, MO, USA) (10 µg of Ag, Al, As, B, Ba, Be, Bi, Ca, Cd, Ce, Co, Cr, Cu, Dy, Er, Eu, Fe, Ga, Gd, Ge, Ho, In, K, La, Li, Lu, Mg, Mn, Mo, Nb, Nd, Ni, Pb, Pr, Rb, Re, Rh, Ru, S, Sc, Se, Si, Sm, Sr, Ta, Tb, Th, Ti, Tl, Tm, U, V, W, Zn, Y, Yb and Zr). The study was performed with the following elution protocol: rinsed with 3 BV of 1 M Citric acid, 3 BV of 2 M HCl, 3 BV of 3 M HCl, 2 × 3 BV of 2.5 M HNO_3_, 2 × 10 BV of 6 M HNO_3_.

#### 4.5.3. Analysis

After elution from the column, a portion of the load, rinse and elution solutions were diluted to 4 or 10 mL with 2% nitric acid, and ICP-OES analysis was performed according to published analytical methods [24]. For all ICP-OES analysis methods a standard curve and limit of quantification were determined as previously described [24]. Wavelengths for each element and quantification limits for each element are provided in the Supplementary Materials. study 1: ICP-OES methods were established for two methods using PE Pure Plus atomic spectroscopy calibration multi-element ICP-MS standards numbered 2, 3 and 5. One method contained “the lanthanides”: Sc, La, Ce, Lu, Th, Dy, Er, Eu, Gd, Ho, Nd, Pr, Sm, Tb, Th, Tm, Y, Yb, and Rh, with an internal standard of 0.15 ppm In. The other method combined ICP standards 3 and 5 and evaluated elements Pb, Zn, Co, Cr, Cd, Ni, Fe, Mn, Al, Ga, Ge, In, Sr, Rb, Ba, Nb, Be, Cu, Mg, K, V, Li, U, Tl, As, Se, Bi, B, Mo, Re, S, Ta, Ti, Th, W, Zr and Ag with an internal standard of Sc. Study 2 & 3. In studies 2 & 3, three methods were developed for periodic table mix 1, 2 and 3 (Sigma Aldrich). Methods for periodic table mix 1 and 2 used 0.15 ppm scandium as the internal standard, and periodic table mix 3 used 1 ppm Indium as the internal standard. During ICP-OES method development, all wavelengths for each element being analyzed were evaluated. Wavelengths with the lowest quantification limits were used for the analysis of each element of the method. The acceptance criteria for the true concentration value and the % relative standard deviation (RSD) was within 10%. 

#### 4.5.4. Evaluation of Optimized Elution Procedure with ^225^Ac, Th, ^223^Ra and Other Elements

A working solution of thorium was prepared by dissolving 25 g of thorium nitrate in 25 mL of water. In a vial, 1 mL of periodic mix 1 and 1 mL of periodic mix 3 was added and the solution was evaporated to dryness. Then, 0.25 mL of the thorium solution was added, and a trace amount of ^225^Ac was added, followed by the addition of 6.5 mL of a 1-M citric acid solution with the pH of the solution adjusted to 1.5–2.0. A 1-mL AG50 × 8 column was prepared and the solution was added to the column. The following sequence was used to elute the column: 5 mL of 1 M citric acid pH 2, 3 mL of water, 3 mL of 2 M HCl, 3 mL of 3 M HCl, 3 mL of water, 2 × 3 mL of 2.5 M nitric acid and 2 × 10 mL of 6 M nitric acid. The elements in the different fractions were quantified by ICP-OES or gamma spectroscopy. The experiment was repeated with the following rinse sequence: 5 mL of 1 M citric acid pH 2, 3 mL of water, 3 mL of 2 M nitric acid, 2 × 3 mL of 2.5 M nitric acid and 2 × 10 mL of 6 M nitric acid. The experiment was repeated with the following rinse sequence: 5 mL of 1 M citric acid pH 2, 3 mL of water, 2 × 3 mL of 2.5 M HCl, 3 mL of water, 2 × 3 mL of 2.5 M nitric acid and 2 × 10 mL of 6 M nitric acid.

#### 4.5.5. Analysis

The separation fractions containing ^225^Ac, ^227^Th and ^223^Ra were quantified after 24 h by gamma spectroscopy at 236 (^227^Th), and 269.6 keV (^223^Ra). At the time of analysis, ^225^Ac and its daughters (specifically ^221^Fr and ^213^Bi) were at equilibrium with ^225^Ac, and the gamma peak at 440 KeV for Bi-213 was used to quantify ^225^Ac. The 218 KeV gamma peak for ^221^Fr was used to quantify ^225^Ac and similar results were obtained. Elemental analysis was performed to determine the elution of various elements (wavelengths used in analysis of elements are summarized in Supplementary Tables S2–S5). 

## 5. Conclusions

In this series of experiments, various conditions were evaluated to optimize the cation separation step of the ^225^Ac separation. The optimal pH of the load solution was between 1.5 and 2, and the pH should be monitored, as it influences the ^225^Ac yield. Three rinse sequences were evaluated, and all three rinse sequences might be able to replace the MP1/HCl step of the ^225^Ac separation. None of the three sequences could separate lanthanides from ^225^Ac, so a subsequent BDGA separation step is needed. The optimized rinse sequence for the separation step was: 5 BV of 1 M citric acid pH 2.0, 3 BV of water, 3 BV of 2 M HNO_3_, 6 BV of 2.5 M HNO_3_ and 20 BV of 6 M HNO_3_. The rinse sequence removed thorium, and the 2.5-M HNO_3_ rinse step removed >85% of Ba/Ra. The 6-M HNO_3_ rinse step was able to elute >90% of ^225^Ac, and was incorporated into the separation of ^225^Ac from thorium.

## Figures and Tables

**Table 1:** Percentage of eluted elements in the various steps of the separation using the following rinse sequence: 5 mL of 1 M citric acid pH 2, 3 mL of water, 3 mL of 2 M nitric acid, 2 × 3 mL of 2.5 M nitric acid and 2 × 10 mL of 6 M nitric acid. Volume of Ag50 × 8 1 mL. Bolded values are the highest value for the elution of the element, and empty boxes represent samples that were <1% or not detected. NM: not measured.

	Load	Citric	Water	2 M HNO_3_	2.5 M HNO_3_	2.5 M HNO_3_	6 M HNO_3_	6 M HNO_3_
Al	NM	**32.3**		8.5	8.4	4.8	5.6	
Ba	NM		1.8		68.9	29.4	1.3	
Be	NM	**69.9**	3.4	19.3	7.3	2.8	12.1	1.6
Cd	NM	6.6	0.4	28.3	**62.5**	2.8	1.2	
Co	NM			23.8	**65.1**	3.0		
Cr	NM	7.2		2.1	11.9	**29.9**	16.4	
Fe	NM	**83.3**	7.1	3.1	4.9	7.4	8.0	
Mn	NM			10.3	**72.4**	12.5		
Mg	NM			26.3	**68.3**	3.6	1.9	
Ni	NM			46.2	**53.9**			
Pb	NM			**57.4**	32.6	1.2		
^223^Ra	1.5			0.5	**60.7**	36.6		
Zn	NM			43.4	**56.5**	2.8		
**Lanthanides**								
Ce	NM	NM	6.6	4.8	13.3	17.0	**46.0**	12.6
Dy	NM	NM	10.1	3.9	9.0	10.7	**37.1**	13.8
Er	NM	NM	10.8	4.5	10.9	12.8	**44.6**	16.7
Eu	NM	NM	2.6	3.8	8.1	8.8	**15.5**	4.3
Gd	NM	NM	12.1	4.3	10.3	11.4	**44.0**	17.9
Ho	NM	NM	3.8	3.3	7.0	8.0	**22.0**	6.1
La	NM	NM	4.3	5.1	5.1	5.9	31.2	**48.5**
Lu	NM	NM	12.0	3.2	8.6	10.3	**45.7**	20.2
Nd	NM	NM		3.8	8.2	**10.2**	8.3	
Pr	NM	NM	4.6	3.1	4.1	4.7	27.6	**42.1**
Y	NM	NM	10.1	4.8	10.9	12.9	**45.2**	16.5
Yb	NM	NM	11.9	3.1	8.4	10.4	**45.6**	20.6
^227^Th	**65.5**	27.7	1.3			1.3		3.0
^225^Ac						5.8	**83.0**	10.3

**Table 2:** Percentage of eluted elements in the various steps of the separation using the following rinse sequence: 3 mL of 1 M citric acid pH 2, 3 mL of water, 3 mL of 2 M HCl, 3 mL of 3 M HCl, 3 mL of water, 2 × 3 mL of 2.5 M nitric acid and 2 × 10 mL of 6 M nitric acid. Bolded values are the highest value for the elution of the element, and empty boxes represent samples that were <1% or not detected. NM: not measured.

	Load	Citric	Water	2 M HCl	3 M HCl	Water	2.5 M HNO_3_	2.5 M HNO_3_	6 M HNO_3_	6 M HNO_3_
Al	NM	NM		**89.94**						
Ba	NM	NM			1.0	1.8	10.3	**15.9**	7.5	
Be	NM	NM	8.7	**48.0**	12.0	1.2	2.4	3.1	13.4	5.0
Cd	NM	NM		**37.3**						
Co	NM	NM		20.8	**23.7**					
Cr	NM	NM		9.2	**48.8**	4.4	2.1			
Fe	NM	NM	12.6	**14.8**	14.3	1.9	2.4	1.6	2.0	1.9
Mn	NM	NM		25.3	**28.7**					
Mg	NM	NM		46.4	**46.6**		1.1		2.1	1.8
Ni	NM	NM		**41.2**	34.6					
Pb	NM	NM	2.2	**54.1**	3.4	2.4	2.7	3.0	8.4	7.9
^223^Ra	4.45	1.68					11	33	**48.2**	
Zn	NM	NM		**46.4**	3.4					
**Lanthanides**										
Ce	NM	NM			1.7	3.8	14.5	**22.2**	13.0	
Dy	NM	NM	1.1	10.0	**33.8**	11.0	13.9	6.5	2.5	
Eu	NM	NM		5.4	**25.9**	9.9	16.2	7.6		
Gd	NM	NM	0.8	7.0	**32.7**	10.3	17.3	11.3	4.0	1.0
Ho	NM	NM	5.8	11.7	**38.1**	10.9	13.9	7.3	10.6	4.2
La	NM	NM	7.9				8.7	24.3	**46.9**	6.3
Lu	NM	NM	1.8	11.7	**33.6**	5.7	6.4	2.4	3.3	2.3
Nd	NM	NM	1.3	12.2	**32.1**	7.8	7.6	1.7		
Pr	NM	NM	1.4	1.6	13.3	7.6	**16.9**	15.0	10.3	
Tm	NM	NM	1.3	11.4	**30.4**	7.4	7.8	2.3	1.0	
Y	NM	NM		6.9	**41.2**	12.0	14.5	4.3		
Yb	NM	NM		11.7	**29.2**	6.3	6.4	1.8		
^227^Th	**58**	23	3.7	0.6	1.1		1	1.3	8.1	2.5
^225^Ac							1.7	7.8	**80.4**	10

## Supplementary Materials

The following are available online, Figures S1–S154: Elution profiles of various elements in various conditions; Table S1: Table of elution profiles of elements for a rinse sequence; Tables S2–S5: Quantification limits for different ICP-OES methods used for the analysis.
