# Supplementary material for: Optimization of Cation Exchange for the Separation of Actinium-225 from Radioactive Thorium, Radium-223 and Other Metals
Source: Molecules. 2019 May 18;24(10):1921. doi: 10.3390/molecules24101921 (PMC6571705; doi:10.3390/molecules24101921)
Supplement: Supplementary file 1 [file molecules-24-01921-s001.pdf]

Supplemental information

Development of elution profile of La/Ac on a Cation column (AG50X8)

Methods

Studies with 50+ elements

Large mixed metal studies with 55 elements: Used 150 µg of Ag, Al, As, B, Ba, Be, Bi, Ca, Cd, Ce, Co, Cr, Cu, Dy, Er, Eu, Fe, Ga, Gd, Ge, Ho, In, K, La, Li, Lu, Mg, Mn, Mo, Nb, Nd, Ni, Pb, Pr, Rb, Re, Rh, Ru, S, Sc, Se, Si, Sm, Sr, Ta, Tb, Th, Ti, Tl, Tm, U, V, W, Zn, Y, Yb, Zr.

55 element Study 1: Examine influence of initial acid on the retention of metals and evaluate retention of lanthanides with multiple rinses of 2.5 M nitric acid. Evaporated metal to a residue, added 25 mL of Conc HCl evaporated. Dissolved residue with 1 M Citric acid pH 2, loaded on AG50 column, rinsed with 3 BV of 1 M Citric acid, 3 BV of 2 M HCl, 3 BV of 3 M HCl, 3 BV of 3 M HCl, 3 BV of 2.5 M HNO_3, 3 BV of 2.5 M HNO3, 3 BV of 2.5 M HNO_3, 3 BV of 2.5 M HNO_3, 10 BV of 6 M NO_3, 10 BV of 6 M HNO_3. Repeated study with 25 ml of Coc Nitric acid. (SF47-100)

55 element Study 2 – Optimized elution profile

Evaporated metal, added 25 mL of Nitric acid evaporated. Dissolved residue with 1 M Citric acid pH 2, loaded on AG50 column, rinsed with 3 BV of 1 M Citric acid, 3 BV of 2 M HCl, 3 BV of 3 M HCl, 3 BV of 2.5 M HNO_3, 3 BV of 2.5 M HNO_3, 3 BV of 2.5 M HNO_3, 10 BV of 6 M HNO_3, 10 BV of 6 M HNO_3. (SF100-55)

Analysis: In initial studies ICP-OES was used to evaluate the metals present in the different rinse and elution steps, and graphs were prepared for each steps. To simply the analysis the study involving 55 elements each element was graphed and the elution steps were on the X axis.

Results

Citric acid studies

Purpose: Determine optial pH of Load solution.

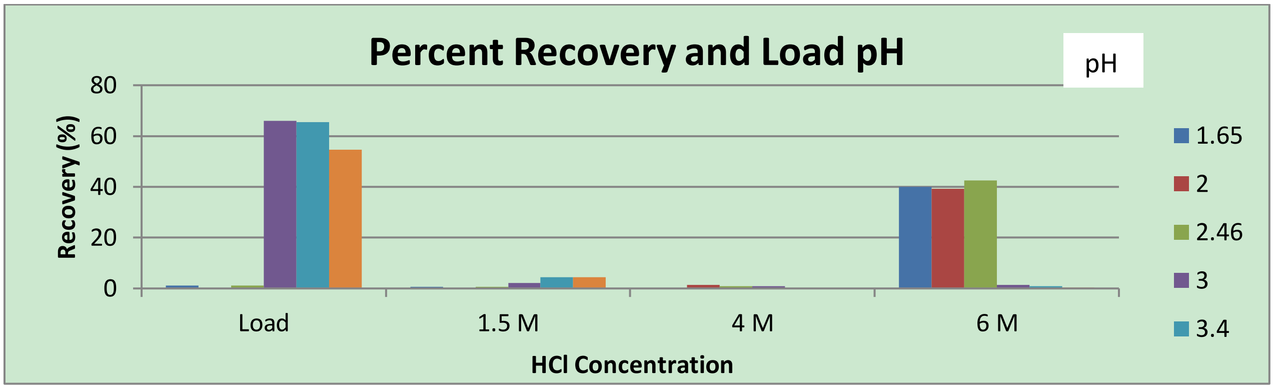

Figure s1. Retention of La on cation exchange resin at different pH values in citric acid.

- Results: Studies with La only solutions indicate a pH from 1.5-2.5 is optimal to retain La, and Load pH values greater than or equal to 3 result in less retention on AG50 resin.

Purpose: Determine optial pH of Load solution with mixed metals and examine elution profile with HCl.

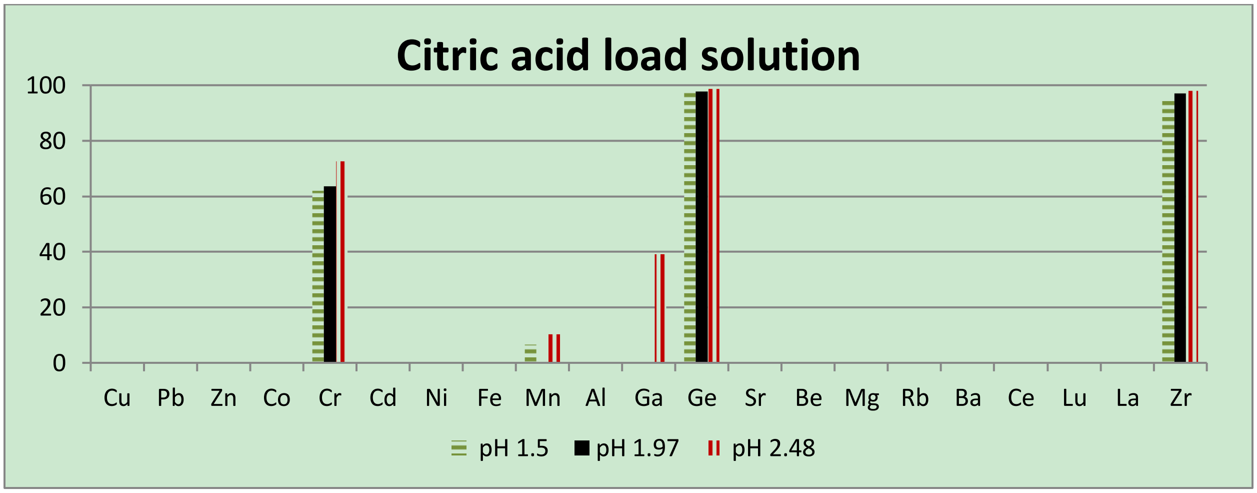

Figure s2. Percent elution of metals in the eluted citric acid solution from a cation column.

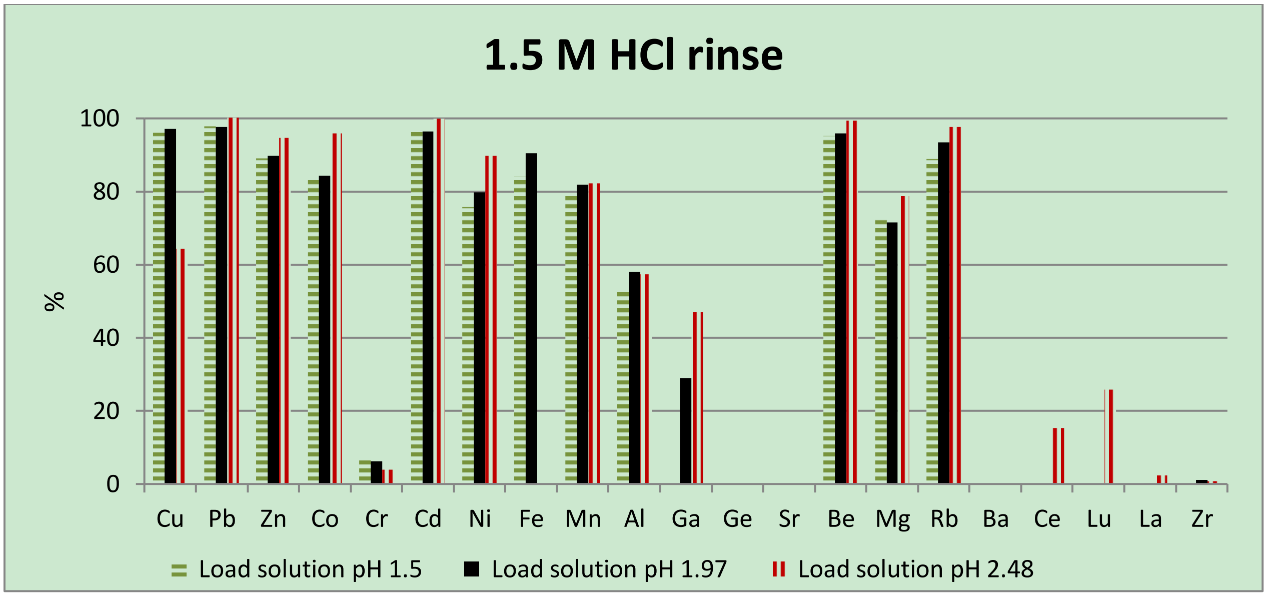

Figure s3. Percent elution of metals in the 1.5 M HCl rinse step from a cation column.

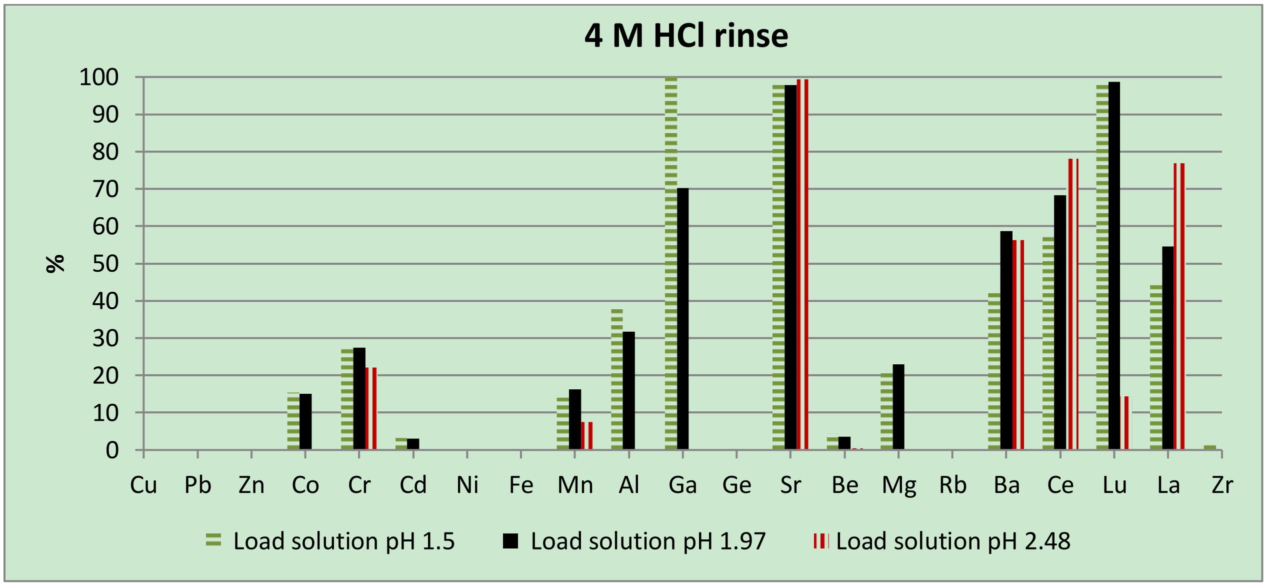

Figure s4. Percent elution of metals in the 4 M HCl rinse step from a cation column.

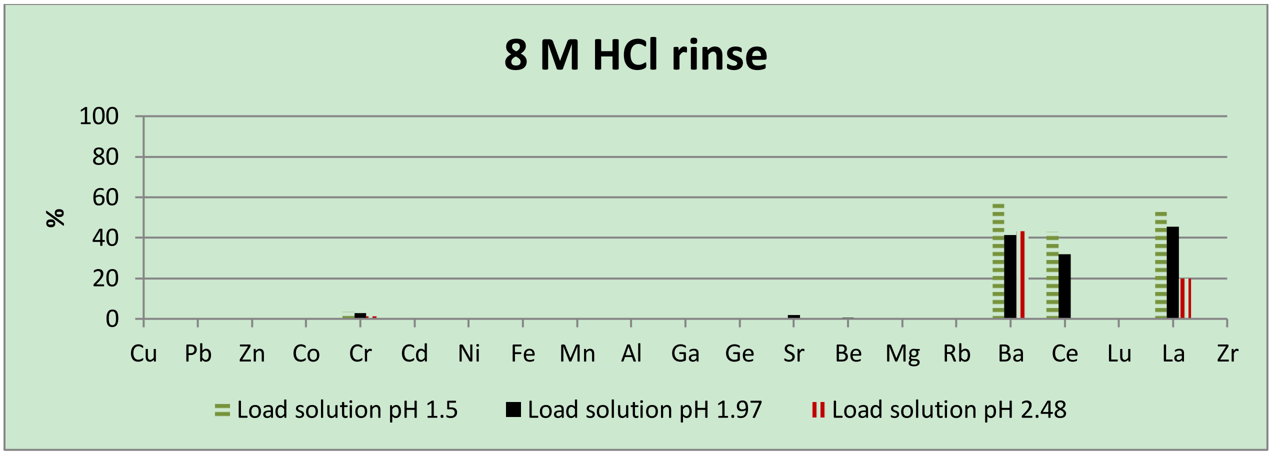

Figure s5. Percent elution of metals in the 8 M HCl rinse step from a cation column.

- Results: The pH of the load solution was best at pH values of 1.5 and 2, and pH 2.5 a small quantity; 2.3% of La was eluted. Many elements were eluted in the 1.5 M HCl rinse, and La was present in the 4 M HCl rinse.

<u>Purpose</u>: Evaluate various rinse solutions. Examined the retention of different elements with varying concentrations of HCl, HNO_3, and H_2SO_4 in the rinse steps. A load solution of citric acid at pH 2 was used for subsequent studies.

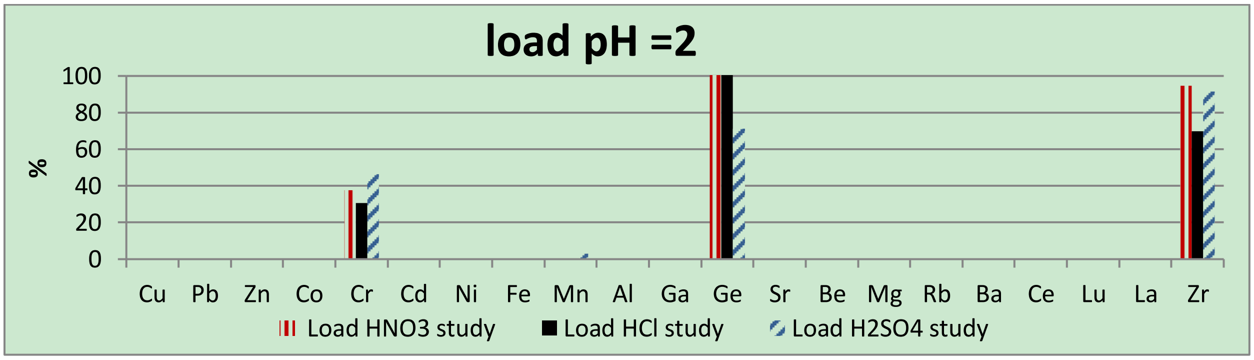

Figure s6. Percent elution of metals in the eluted citric acid solution from a cation column.

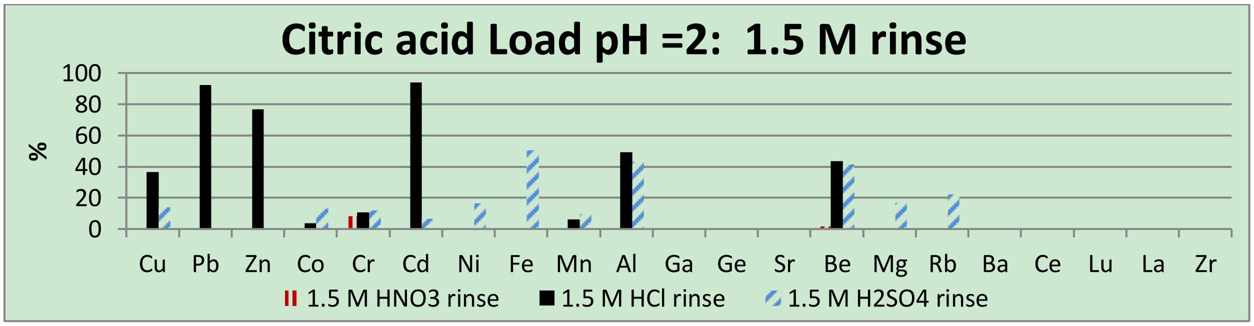

Figure s7. Percent elution of metals in the 1.5 M acid rinse step from a cation column.

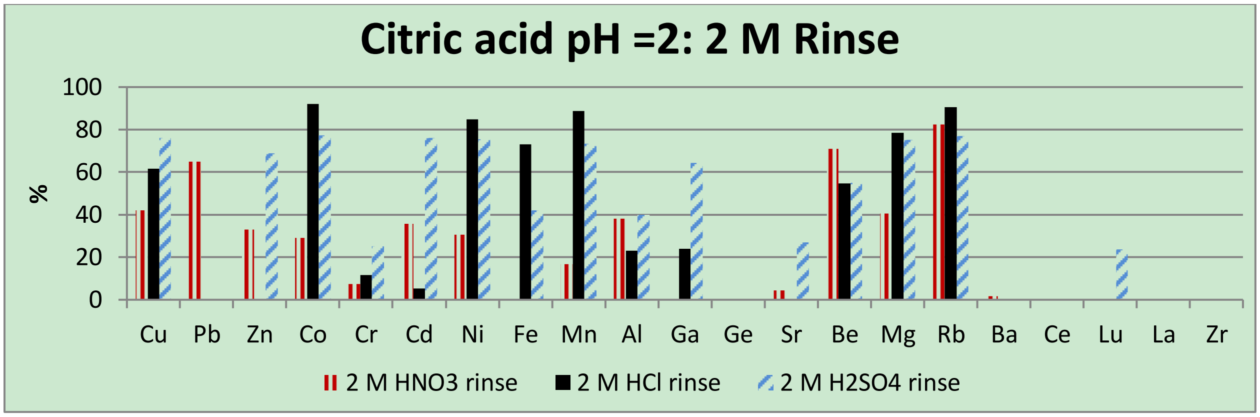

Figure s8. Percent elution of metals in the 2 M acid rinse step from a cation column.

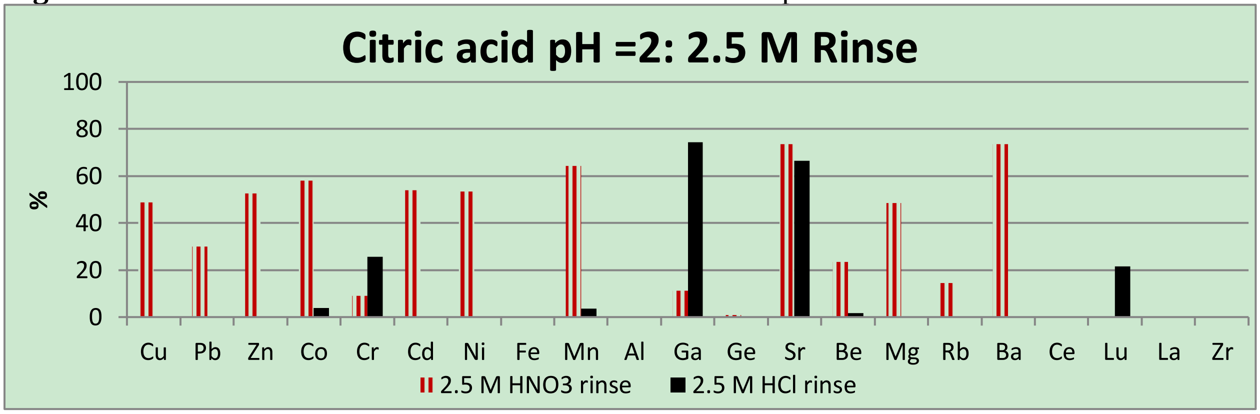

Figure s9. Percent elution of metals in the 2.5 M acid rinse step from a cation column.

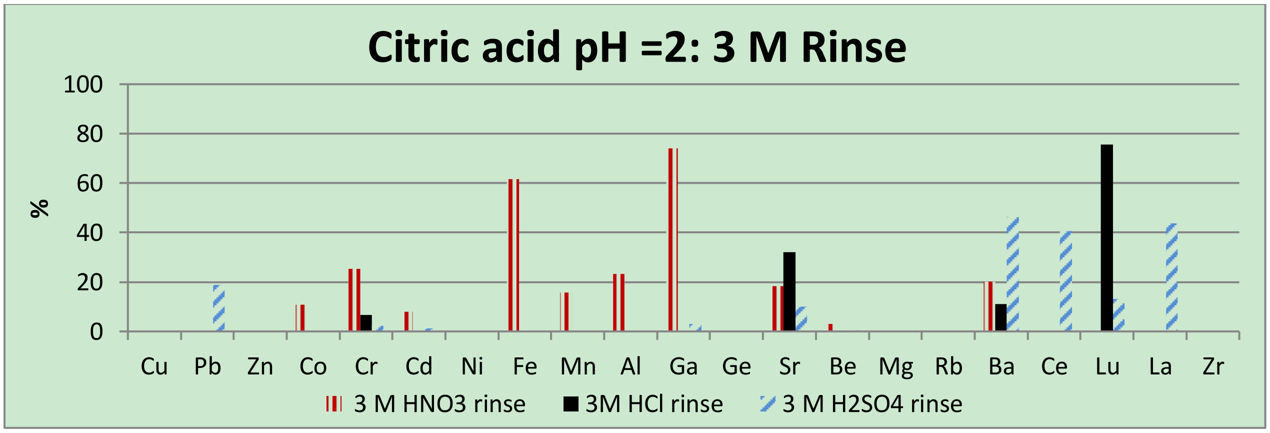

Figure s10. Percent elution of metals in the 3 M acid rinse step from a cation column.

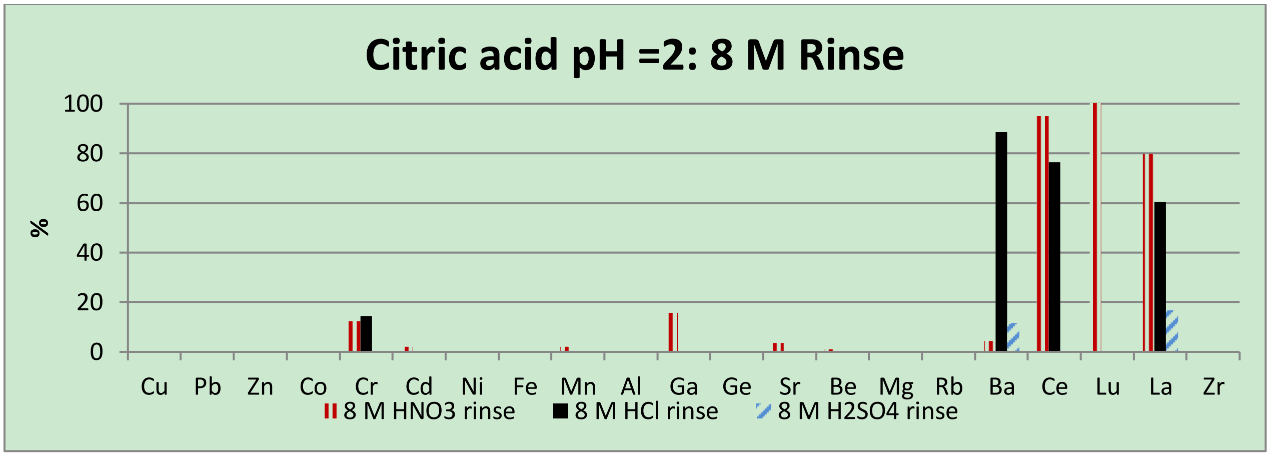

Figure s11. Percent elution of metals in the 8 M acid rinse step from a cation column.

- **Results**: The data indicates rinse solutions with HCl elute the elements at lower molarities than with similar concentrations of HNO_3. Lu can be eluted with 3 M HCl and Ba can be eluted starting with 2.5 & 3 M HNO_3. This set of data indicates a combination of HCl and HNO_3 should be used to elute the elements. La does not elute at concentrations up to 3 M acid.

Tartaric acid testing

Similar testing was performed with tartaric acid. The following graphs summarize the data.

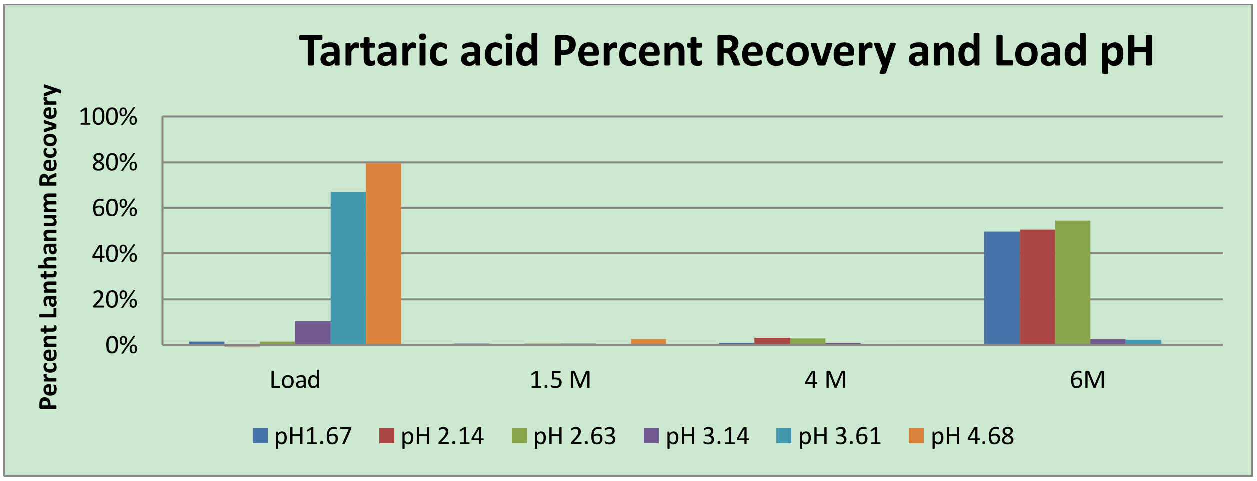

Figure s12. Retention of La on cation exchange resin at different pH values in tartaric acid.

- Results: Studies with La only solutions indicate a pH from 1.5-2.5 is optimal to retain La, and Load pH values greater than or equal to 3 result in less retention on AG50 resin.

Initial Testing Load pH with mixed metals

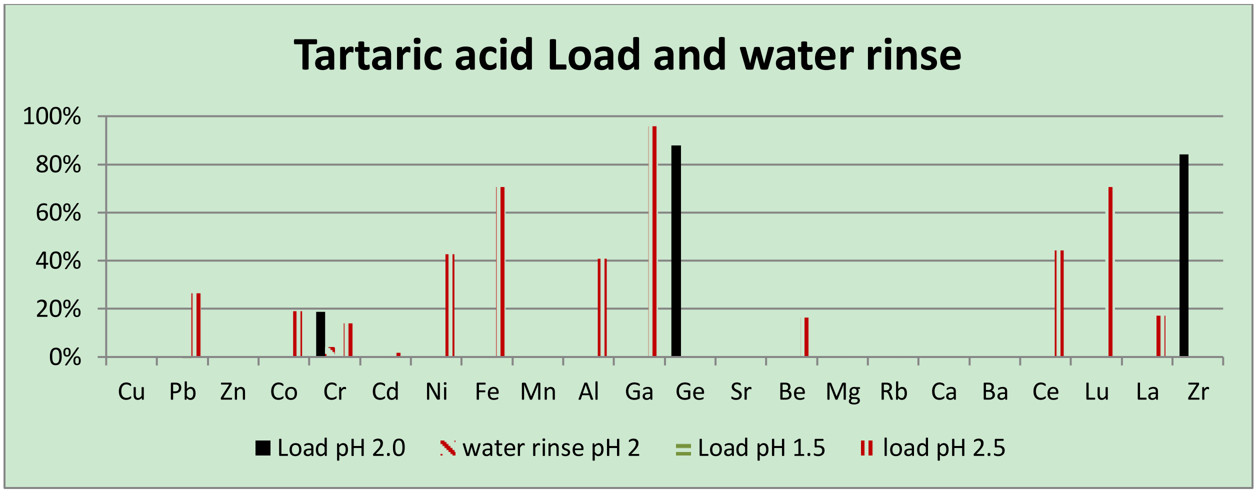

Figure s13. Percent elution of metals in the eluted tartaric acid and water rinse step from a cation column.

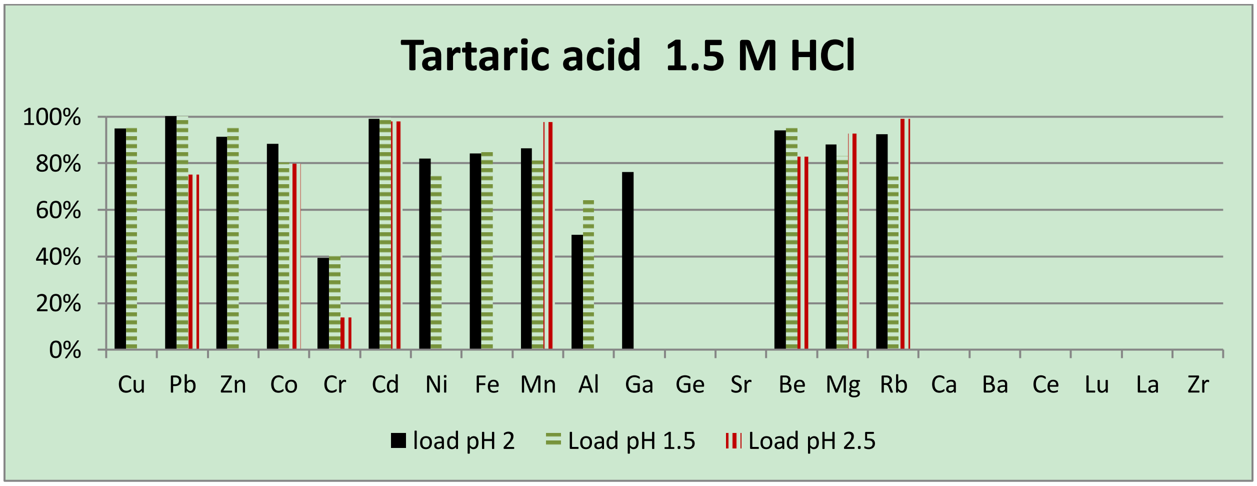

Figure s14. Percent elution of metals in the 1.5 M acid rinse step from a cation column.

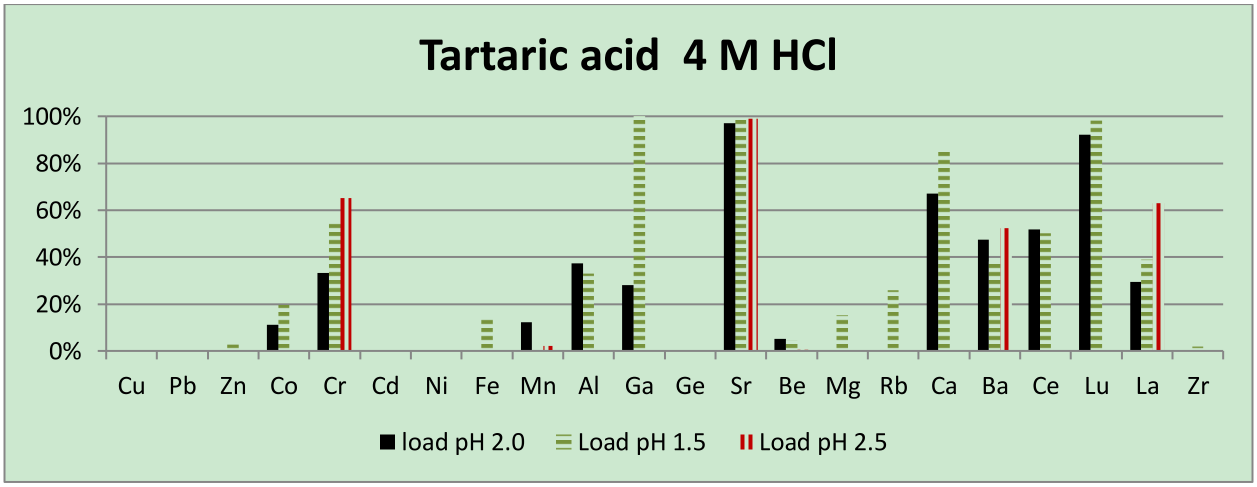

Figure s15. Percent elution of metals in the 4 M acid rinse step from a cation column.

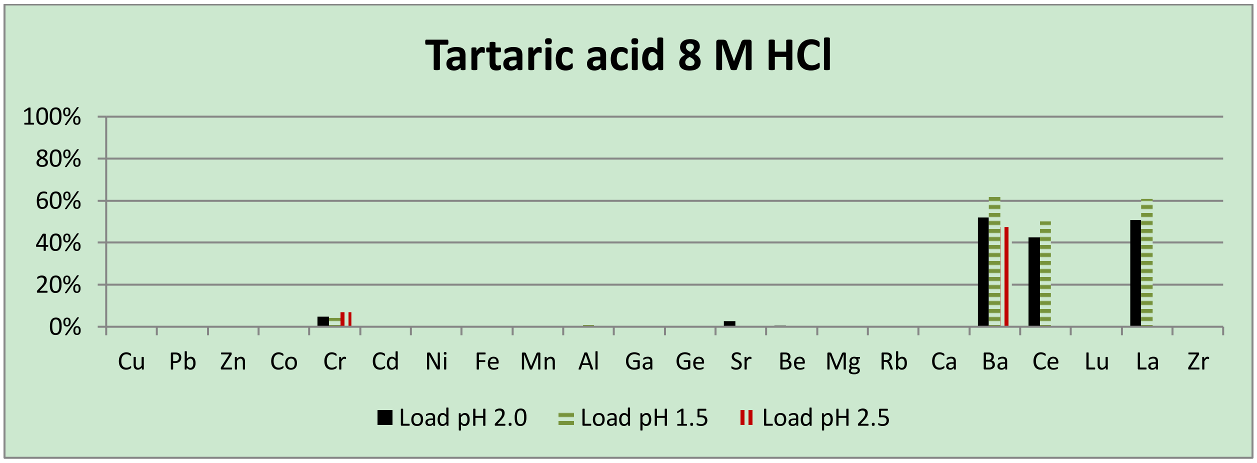

Figure s16. Percent elution of metals in the 8 M HCl acid rinse step from a cation column.

- Results: The pH of the load solution was best at pH values of 1.5 and 2, and at pH 2.5 17% of La was eluted in the load solution. Many elements were eluted in the 1.5 M HCl rinse, and La was present in the 4 M HCl rinse.

<u>Purpose</u>: Evaluate_rinse solutions. Examined the retention of different elements with varying concentrations of HCl, HNO_3, and H_2SO_4 in the rinse steps. A load solution of citric acid at pH 2 was used for subsequent studies. Elutions were performed with 5 bed volumes of rinse solutions**.**

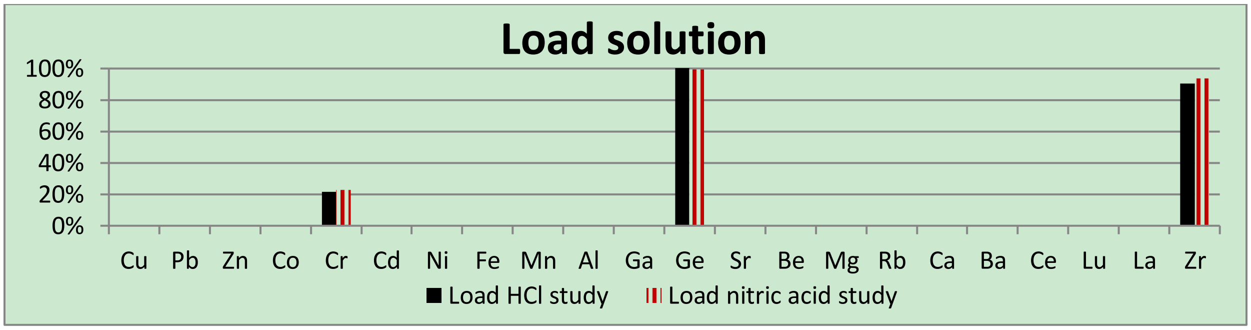

Figure s17. Percent elution of metals in the eluted tartaric acid solution from a cation column.

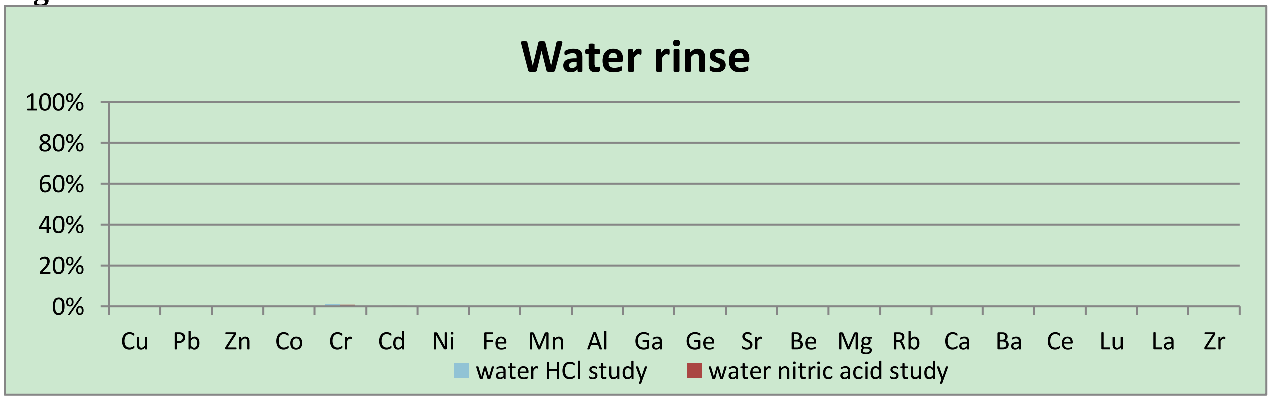

Figure s18. Percent elution of metals in the water rinse step from a cation column

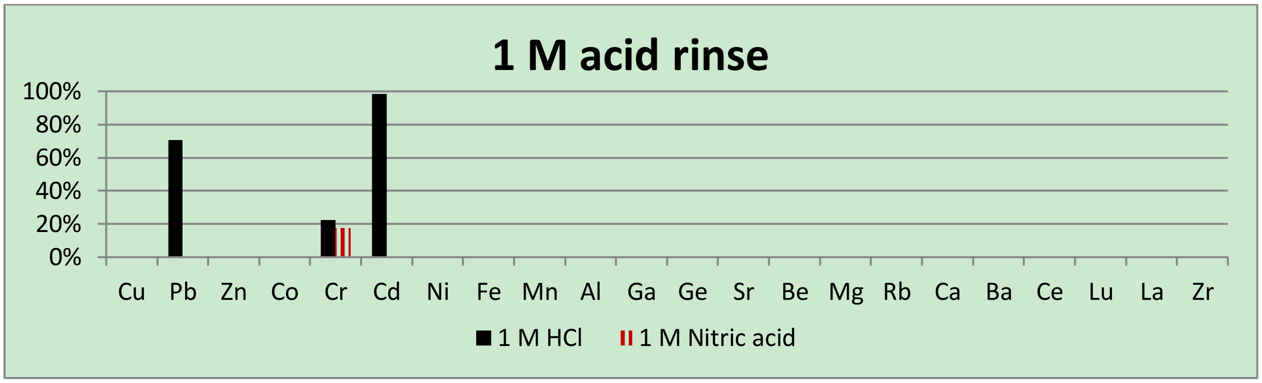

Figure s19. Percent elution of metals in the 1 M acid rinse step from a cation column.

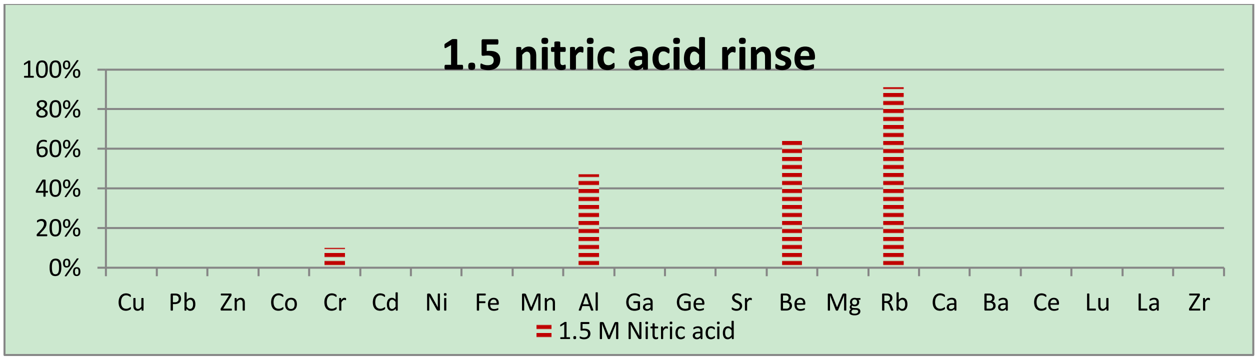

Figure s20. Percent elution of metals in the 1.5 M nitric acid rinse step from a cation column.

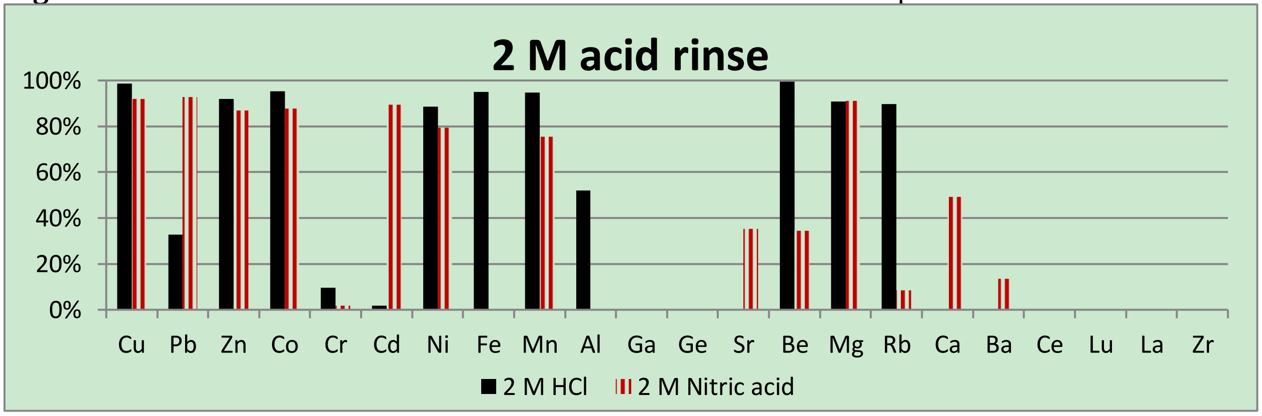

Figure s21. Percent elution of metals in the 2 M acid rinse step from a cation column.

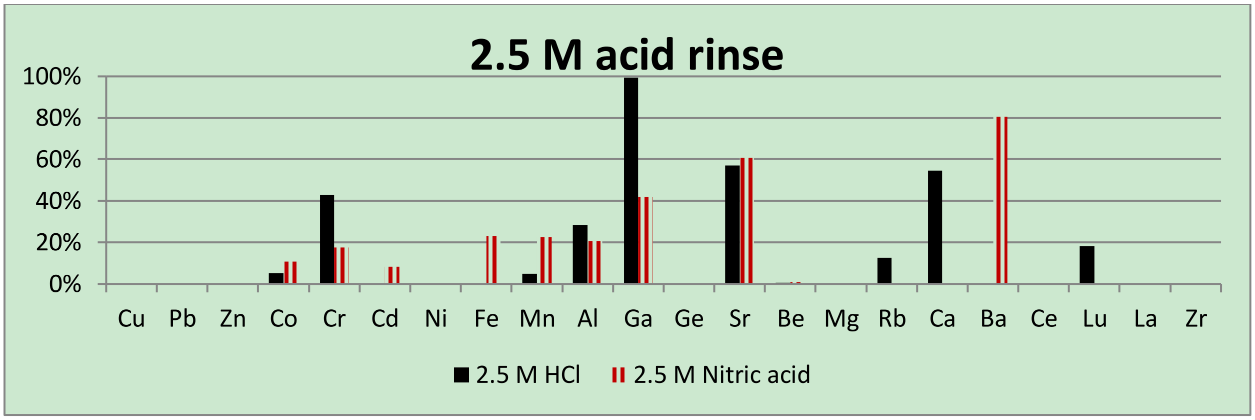

Figure s22. Percent elution of metals in the 2.5 M acid rinse step from a cation column.

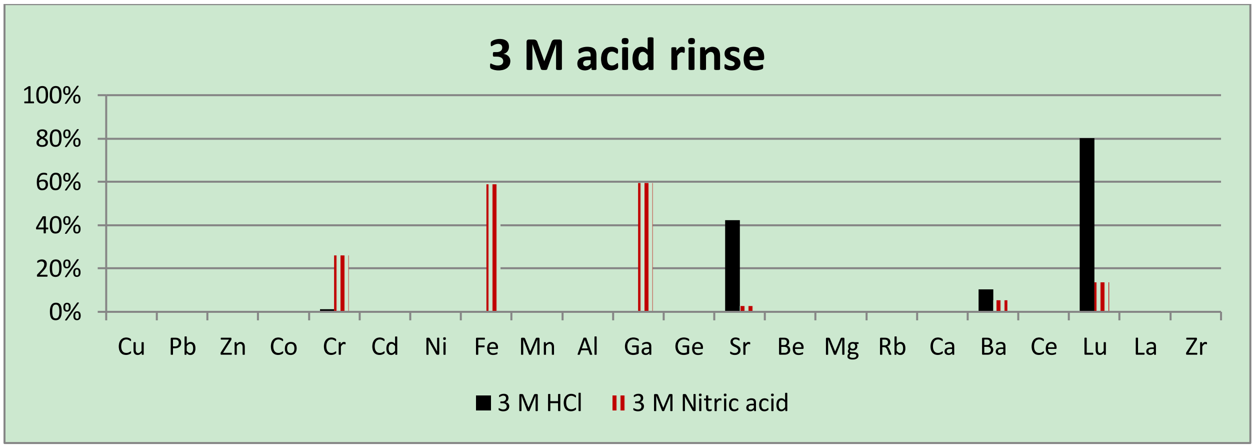

Figure s23. Percent elution of metals in the 3 M acid rinse step from a cation column.

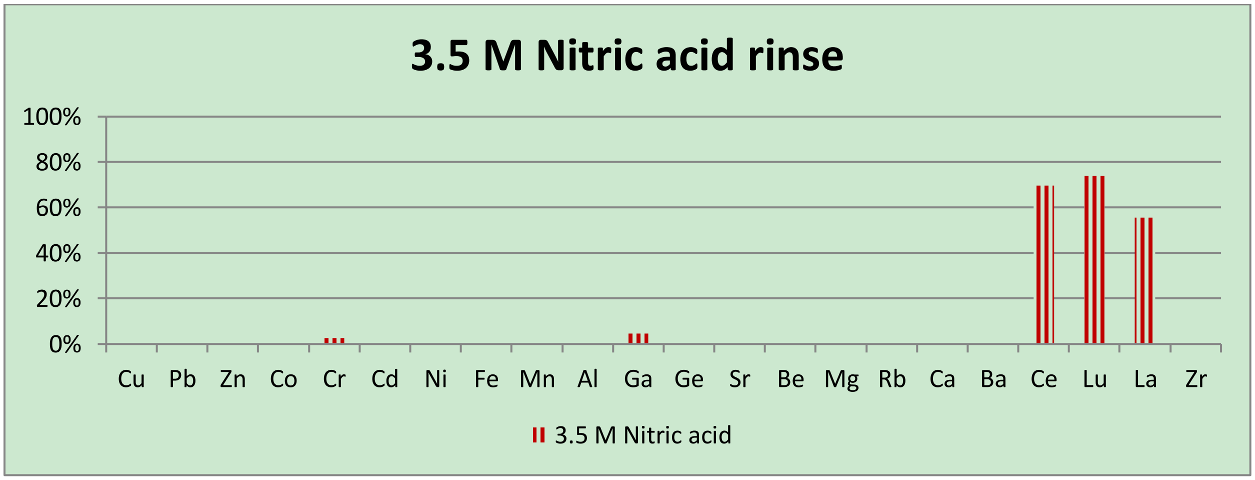

Figure s24. Percent elution of metals in the 3.5 M acid rinse step from a cation column.

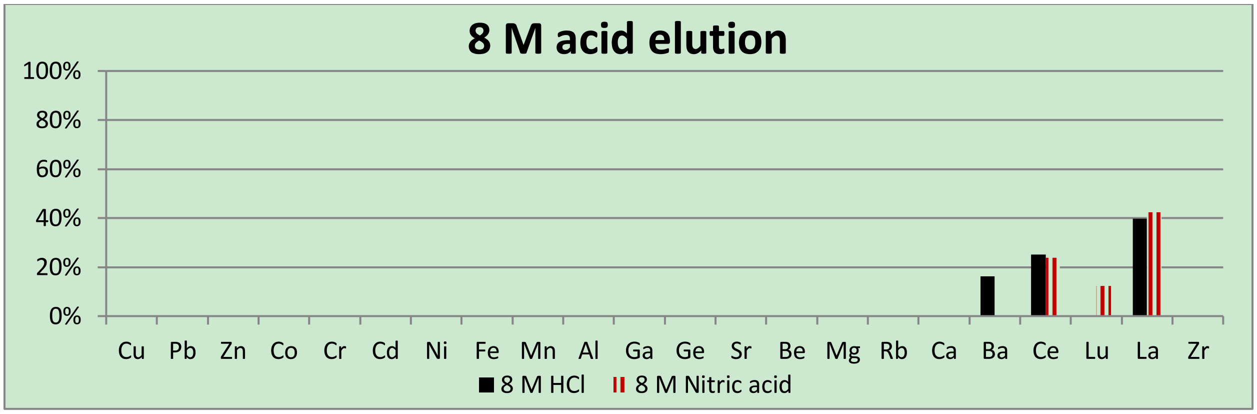

Figure s25. Percent elution of metals in the 8 M acid rinse step from a cation column.

- **Results**: The data indicates rinse solutions with HCl elute the elements at lower molarities than with similar concentrations of HNO_3. Lu can be eluted with 3 M HCl and Ba can be eluted starting with 2.5 & 3 M HNO_3. This set of data indicates a combination of HCl and HNO_3 should be used to elute the elements. La does not elute at concentrations up to 3 M acid.

<u>**Purpose**</u>**:** Testing AG50 versus MP50 with tartaric acid load solution and the rinse sequence HCl then HNO_3.

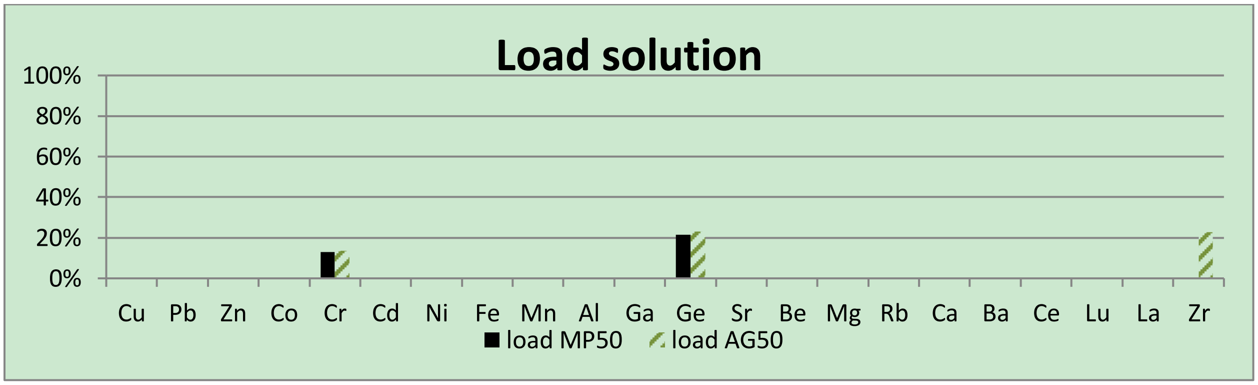

Figure s26. Percent elution of metals in the eluted tartaric acid solution from a cation column.

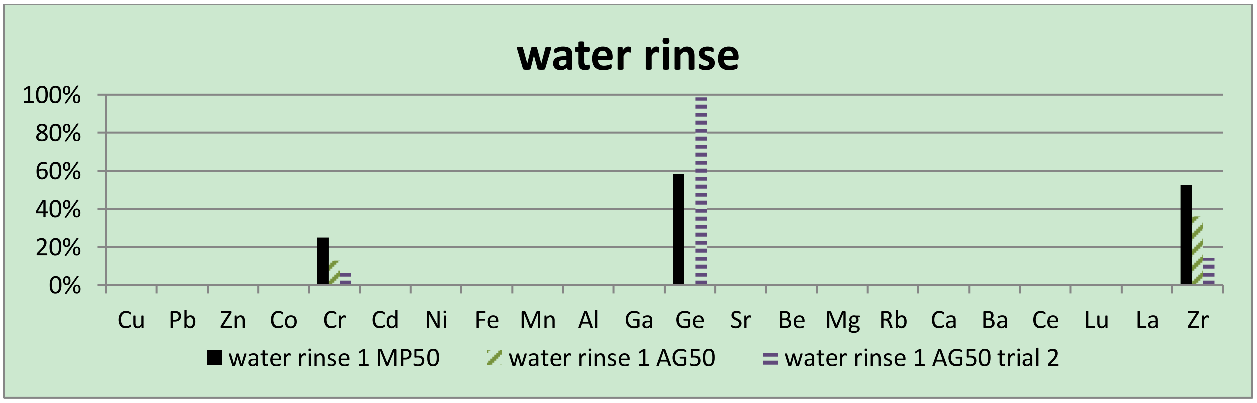

Figure s27. Percent elution of metals in the water rinse step from a cation column.

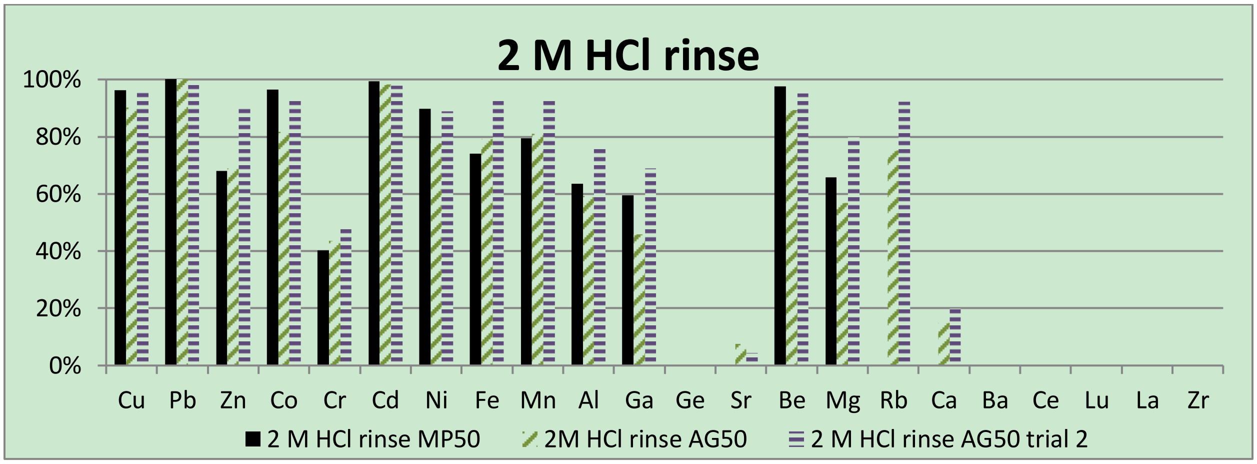

Figure s28. Percent elution of metals in the 2 M HCl acid rinse step from a cation column.

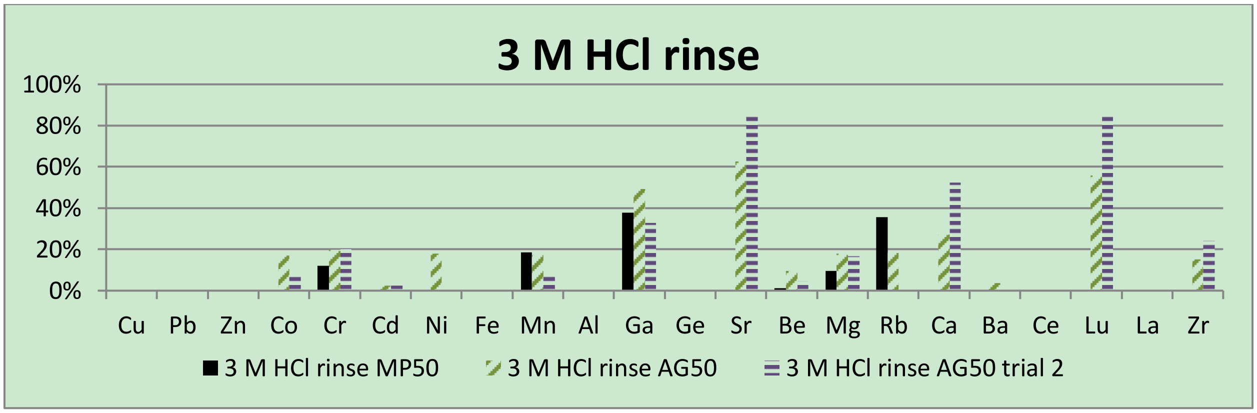

Figure s29. Percent elution of metals in the 3 M HCl acid rinse step from a cation column.

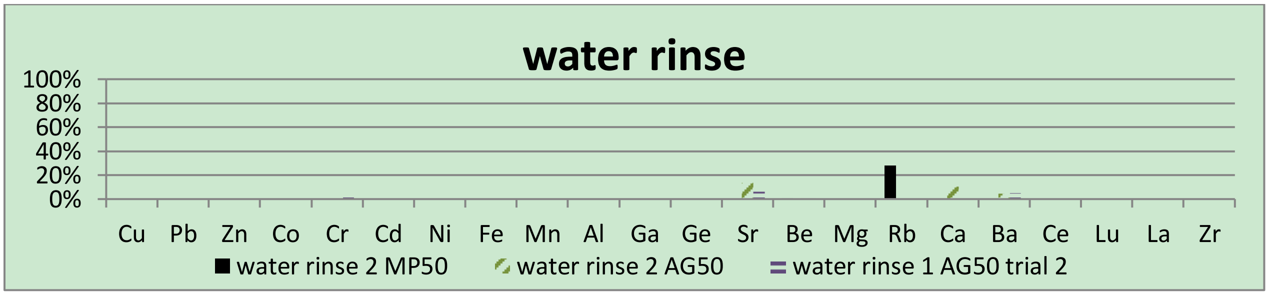

Figure s30. Percent elution of metals in the water rinse step from a cation column.

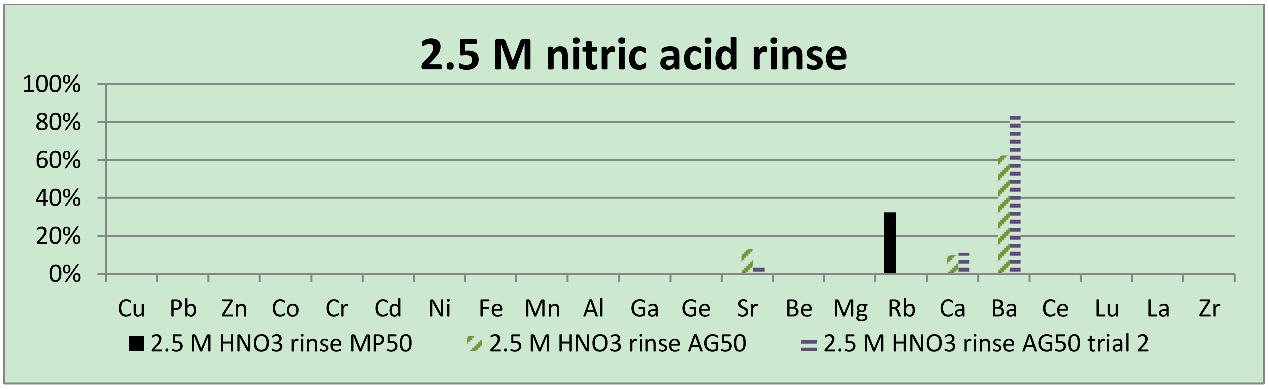

Figure s31. Percent elution of metals in the 2.5 M nitric acid rinse step from a cation column.

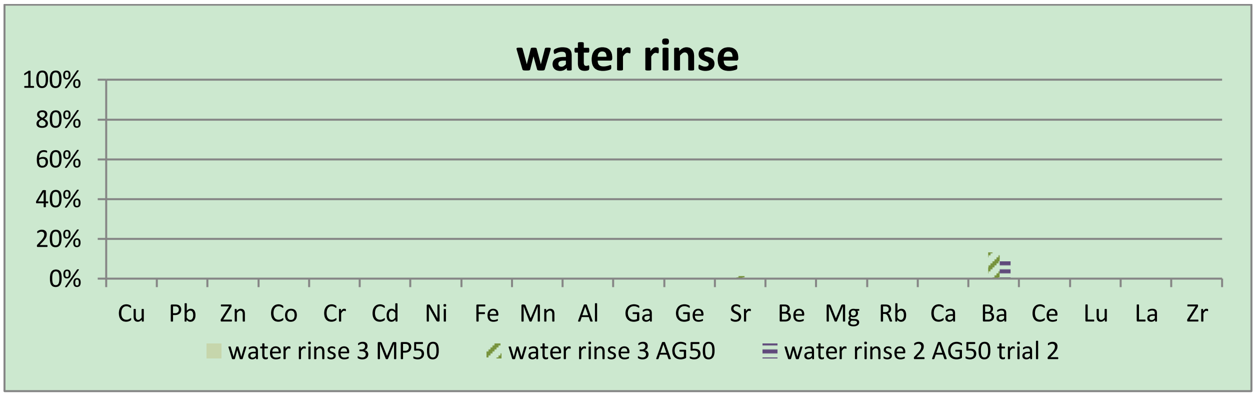

Figure s32. Percent elution of metals in the water rinse step from a cation column.

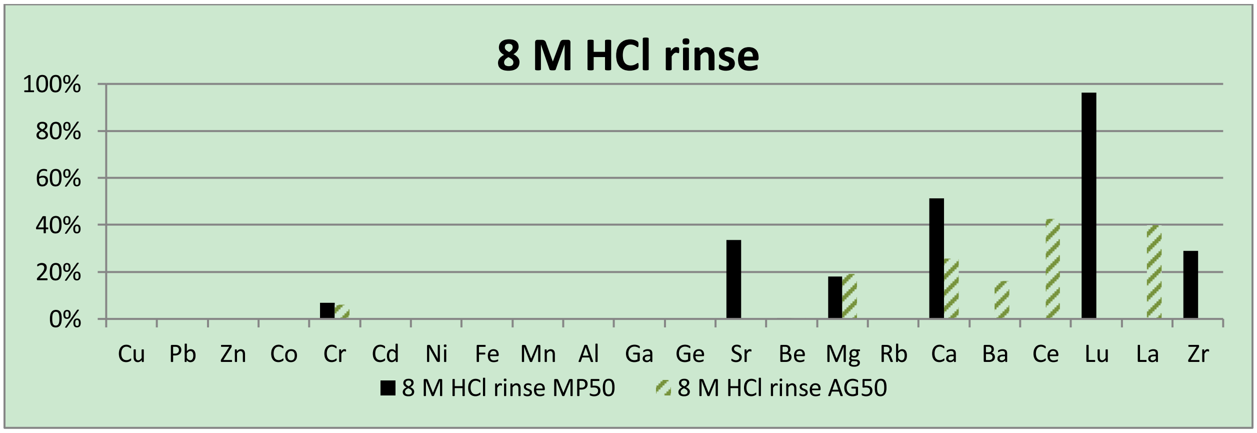

Figure s33. Percent elution of metals in the 8 M HCl acid rinse step from a cation column.

- **Results:** The MP50 resin performed differently then the AG50 resin. Further testing will use AG50.

STUDIES with tartaric and citric acid

Purpose: Testing Elution profile: HCl then HNO_3. The study was setup with a load solution at pH2.0, and the rinse sequence was 3 bed volumes of: 2, 3M HCl then 2.5 M HNO_3 then 8 M HCl.

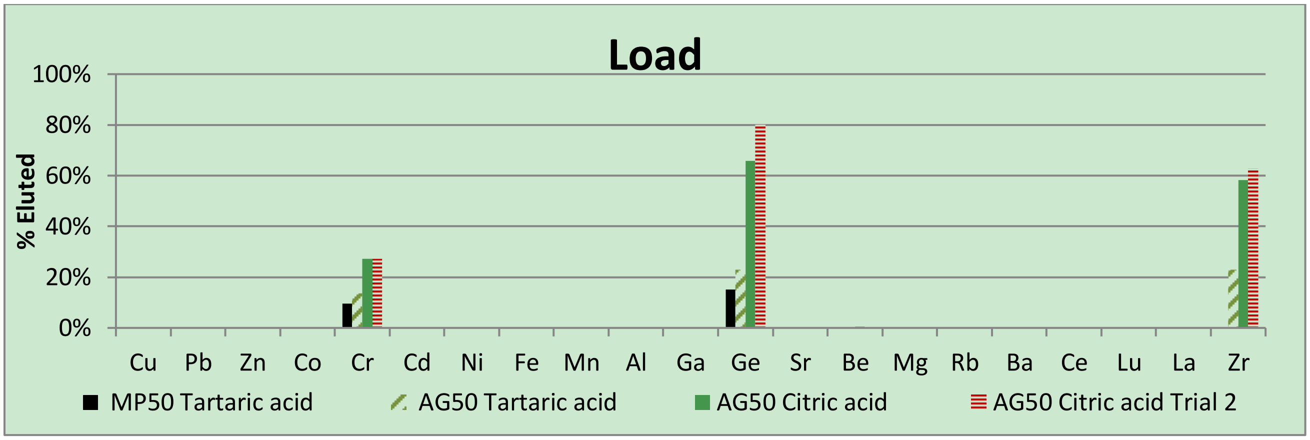

Figure s34. Percent elution of metals in the eluted acid solution from a cation column.

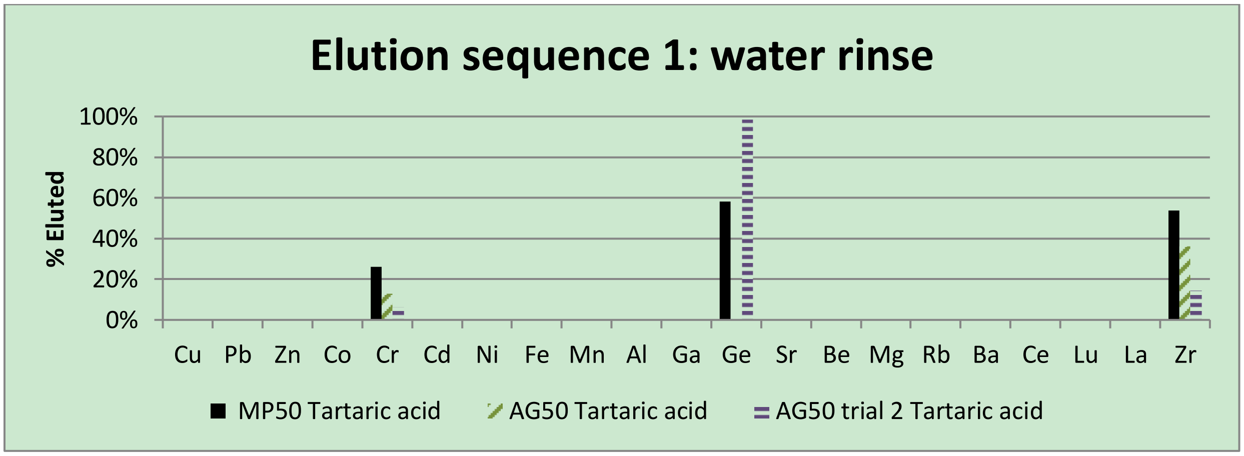

Figure s35. Percent elution of metals in the water rinse step from a cation column.

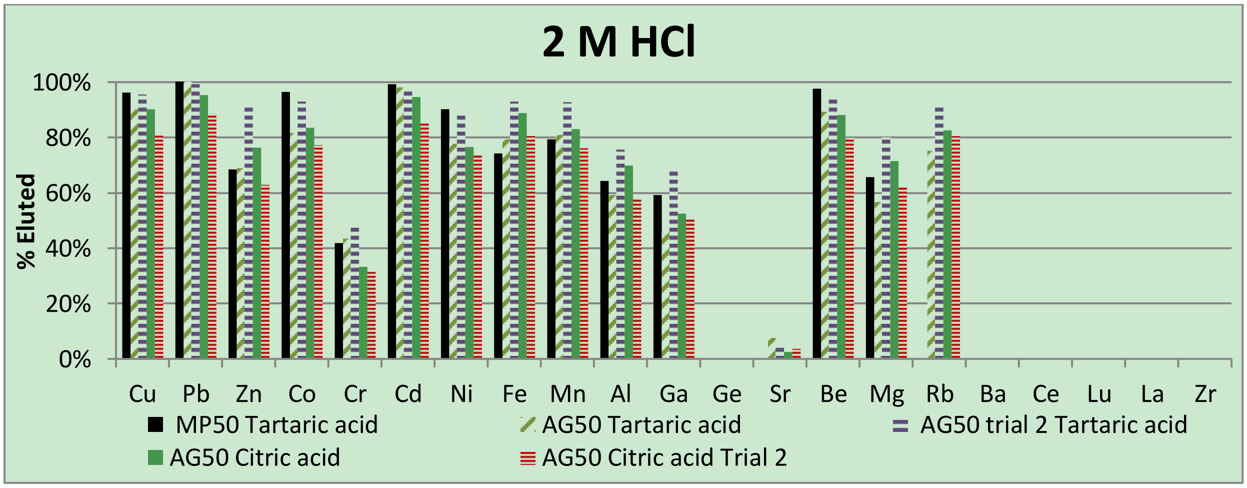

Figure s36. Percent elution of metals in the 2 M HCl acid rinse step from a cation column.

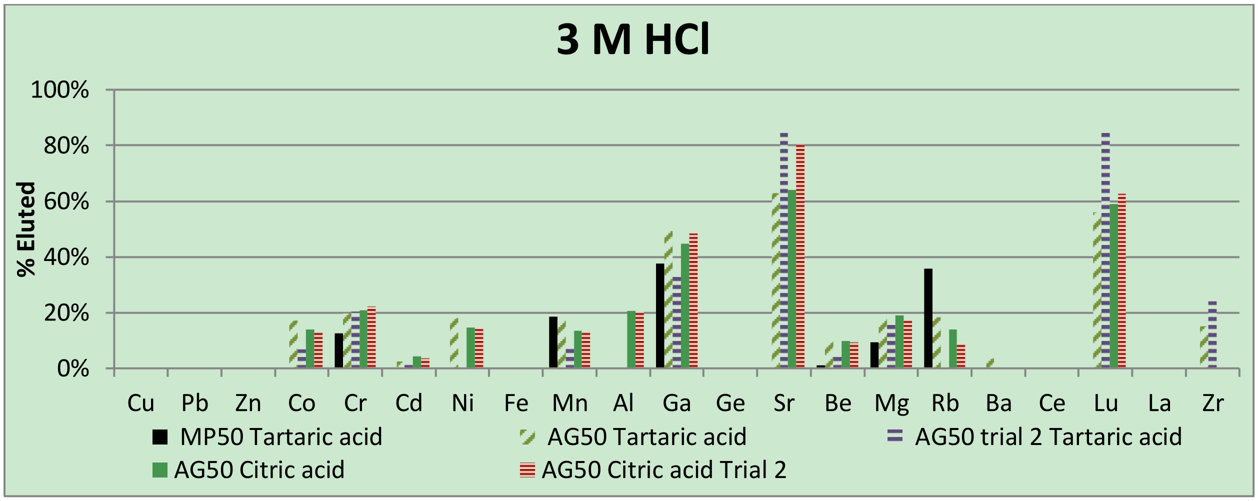

Figure s37. Percent elution of metals in the 3 M HCl acid rinse step from a cation column.

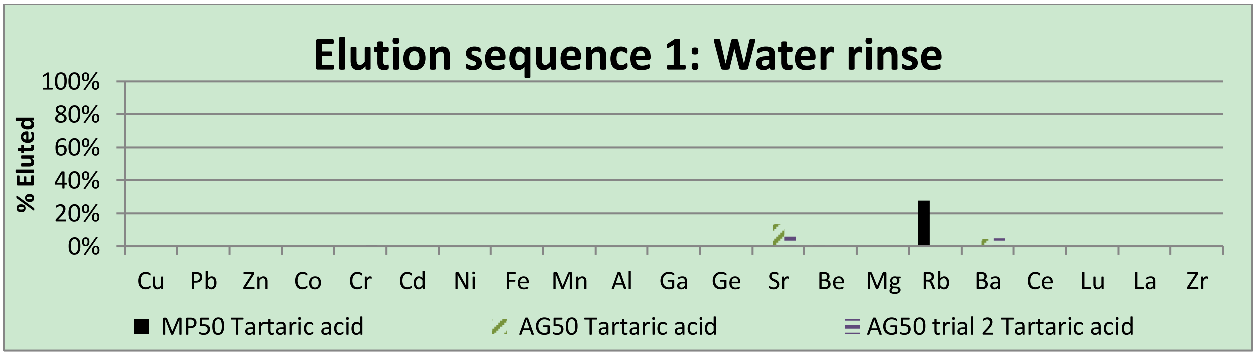

Figure s38. Percent elution of metals in the water rinse step from a cation column.

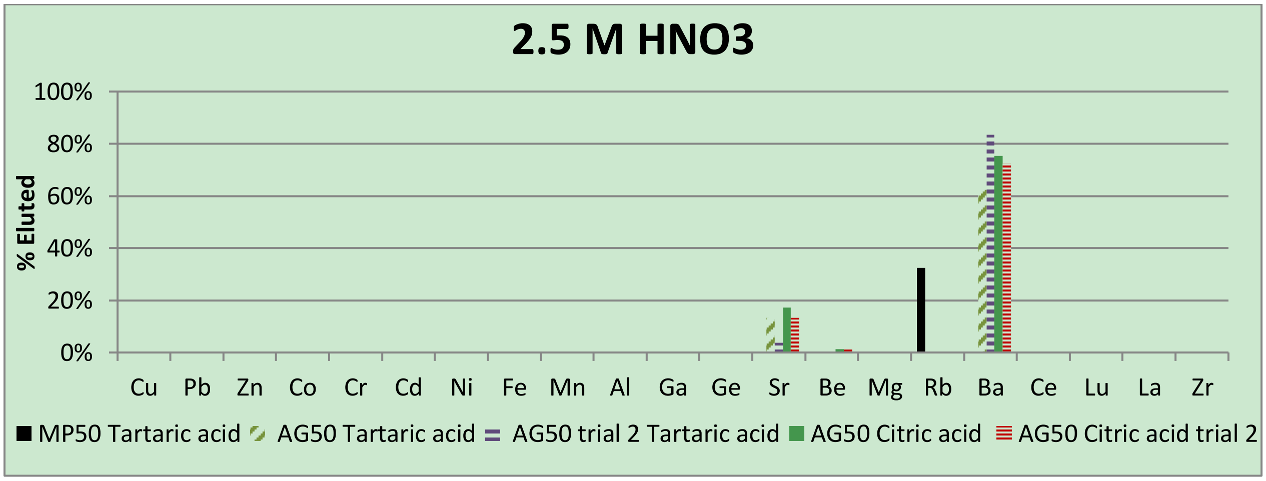

Figure s39. Percent elution of metals in the 2.5 M nitric acid rinse step from a cation column.

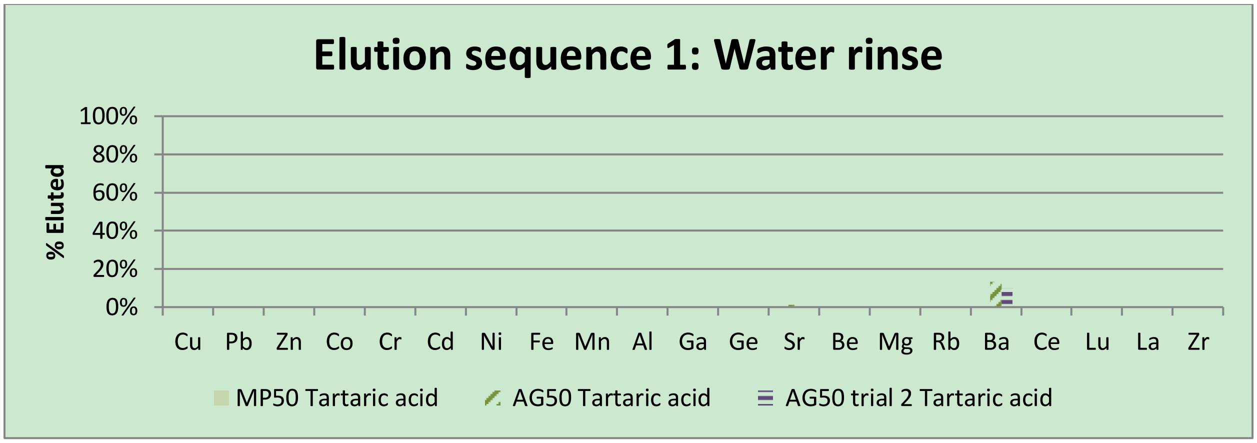

Figure s40. Percent elution of metals in the water rinse step from a cation column.

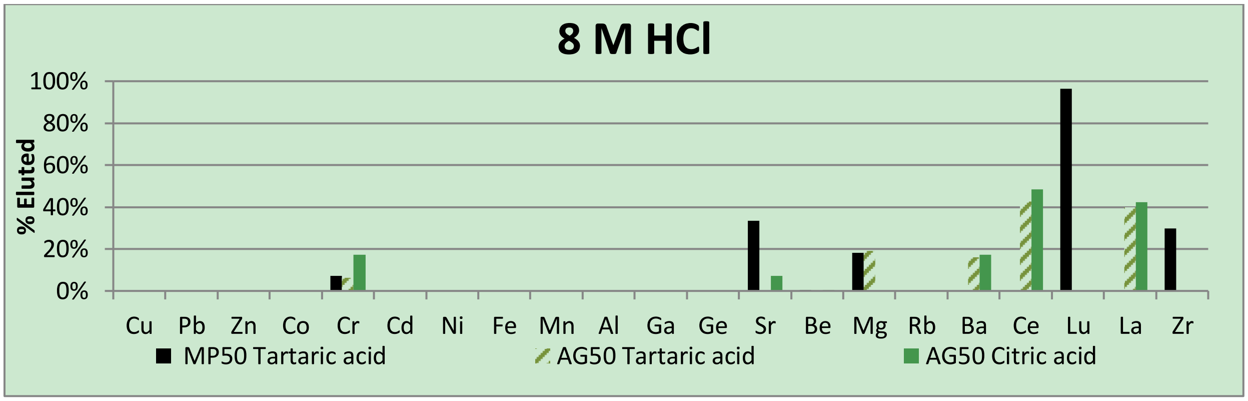

Figure s41. Percent elution of metals in the 8 M HCl acid rinse step from a cation column.

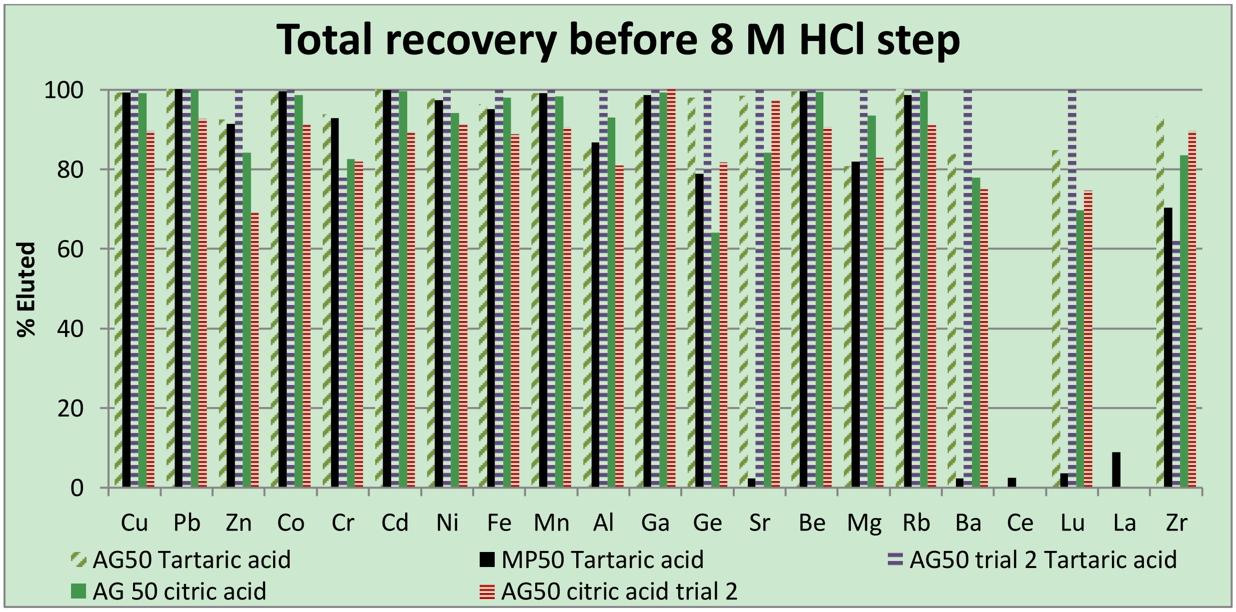

Figure s42. Total Percent elution of metals up to 8 M HCl acid rinse step from a cation column.

- **Results:** The elution profile of 2 & 3 M HCl followed by 2.5 M HNO_3 was able to remove many of the elements that were used in the study while La was retained on the AG50 resin. Performance of Citric and Tartaric acid were similar. The water rinse steps added nothing to the elution profile and can be eliminated. Subsequent studies with 6 M HNO_3 were performed and >10 bed volumes were needed to elute La. Studies examining the elution profile 2.5 M HNO_3 followed by 2 & 3 M HCl have been performed and the data is be graphed.

Citric acid combination nitric then HCl rinse sequence

Comparison of rinse sequence for total removal of metals

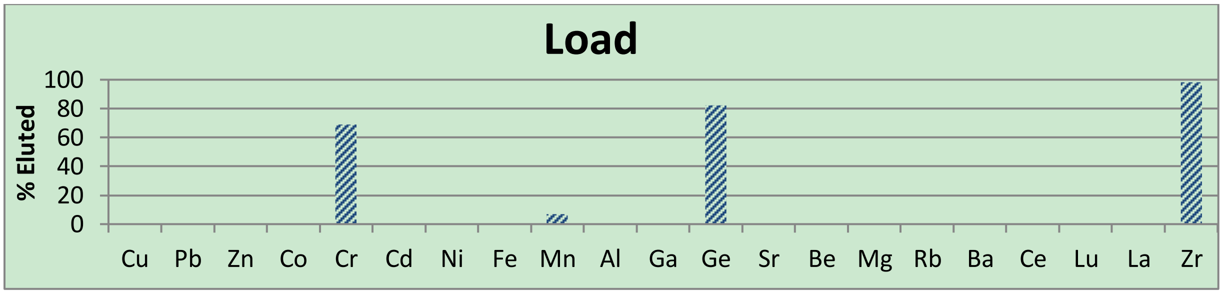

Figure s43. Percent elution of metals in the eluted citric acid rinse step from a cation column.

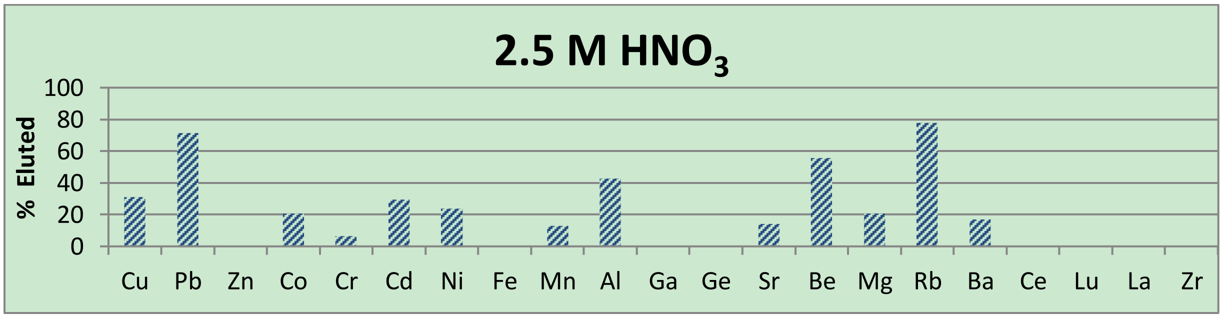

Figure s44. Percent elution of metals in 2.5 M nitric acid rinse step from a cation column.

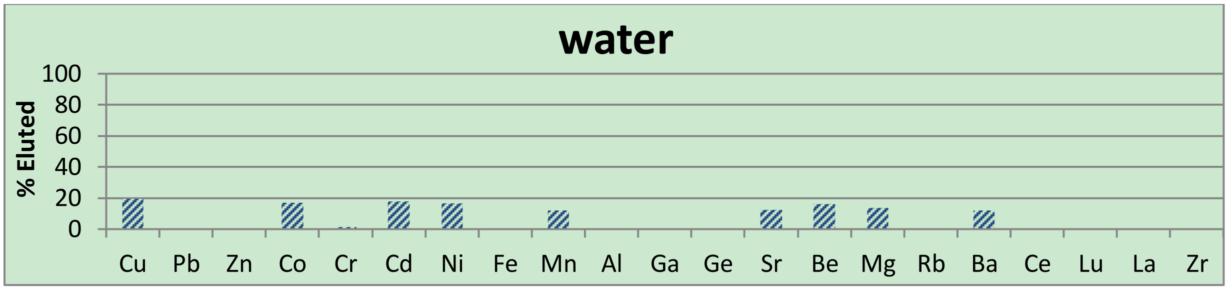

Figure s45. Percent elution of metals in the water rinse step from a cation column.

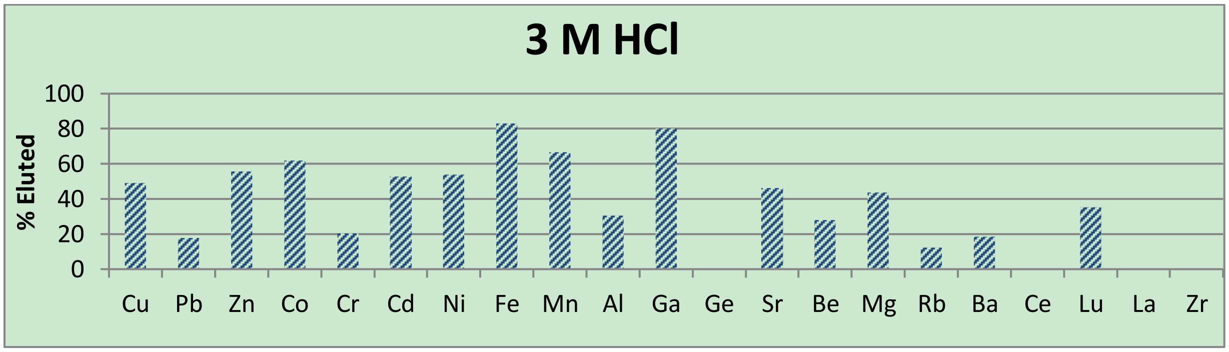

Figure s46. Percent elution of metals in the 3 M HCl acid rinse step from a cation column.

- **Results:** The elution profile of 2.5 M HNO_3 followed by 3 M HCl were able to remove many of the elements that were used in the study while La was retained on the AG50 resin. The water rinse step did elute additional metals, but in less than 20%. The HCl rinses eluted more metals in higher amounts than the nitric rinse step.

Comparison of rinse sequence for total removal of metals

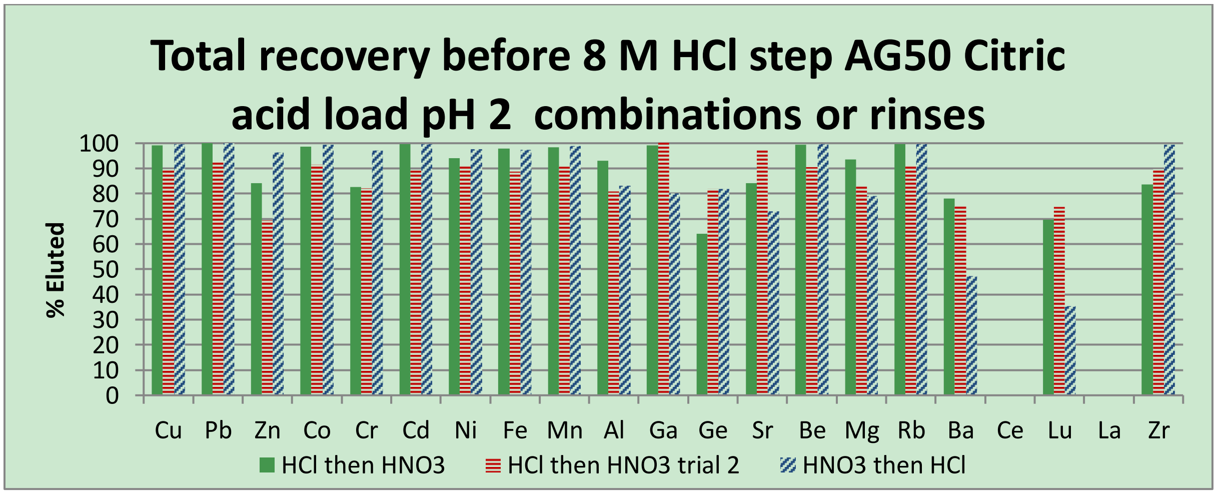

Figure s47. Total Percent elution of metals up to 8 M HCl acid rinse step from a cation column.

- **Results:** The elution profile of 2 & 3 M HCl followed by 2.5 M HNO_3 and 2.5 M HNO_3 followed by 3 M HCl were able to remove many of the elements that were used in the study while La was retained on the AG50 resin. Use the elution sequence 2 & 3 M HCl followed by 2.5 M HNO_3 eluted more Lu and Ba then the elution sequence 2.5 M HNO_3 followed by 3 M HCl. The water rinse steps added nothing to the elution profile and will be eliminated.

Studies of Rh and La on cation ion exchange resin

To a vial was added 50 mL of 1 M citric acid pH 2, 50 µg of La the pH was adjusted to 2 with ammonium hydroxide and loaded onto a one mL AG 50W-X8 column. The column was rinsed with 3 bed volumes of 2 M HCl, 3 BV of 3 M HCl, 3BV of 18MΩ water, 3 BV of 2.5 M HNO_3, 3 BV of 18 MΩ water, and 10 BV of 10 M HNO_3. The study was repeated with 50 µg Rh and La and the elution profile was changed to 3 BV of 2 M HCl, 3BV of 3 M HCl, 3 BV of 18 MΩ water, 3 BV of 2.5 M HNO_3, 10 BV of 3.5 M HCl, 10 BV of 4 M HCl, 10 BV of 5 M HNO_3, and 10 BV of 6 M HNO_3.

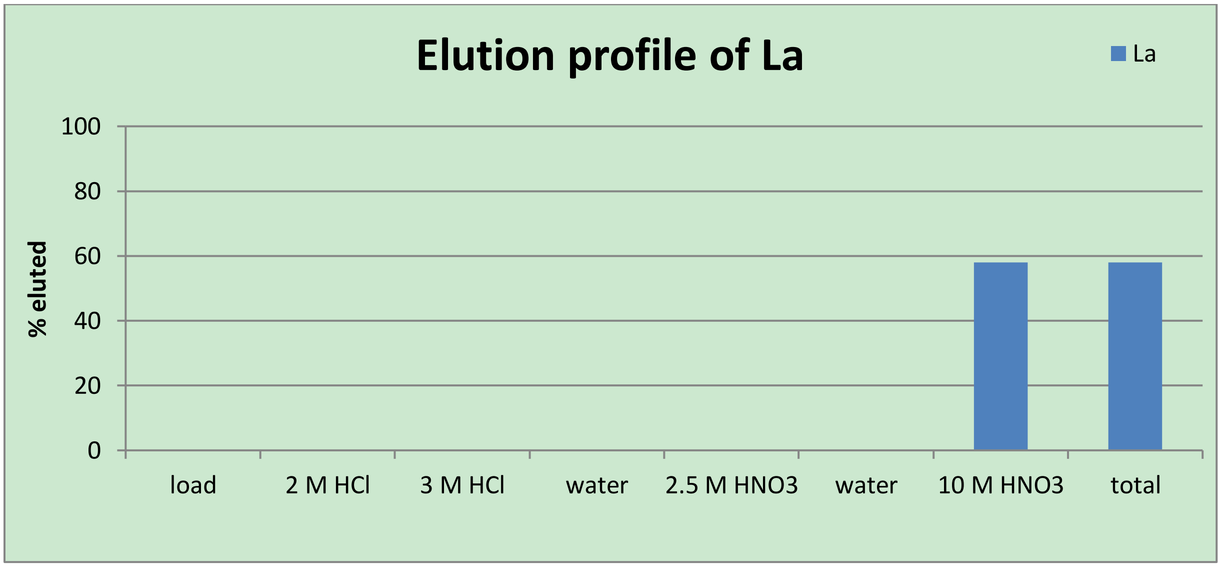

Figure s48. Elution profile of La with 3 BV of 2 M HCl, 3 BV of 3 M HCl, 3 BV of 18 MΩ water, 3 BV of 2.5 M HNO_3, 3 BV of 18 MΩ water, and 10 BV of 10 M HNO_3.

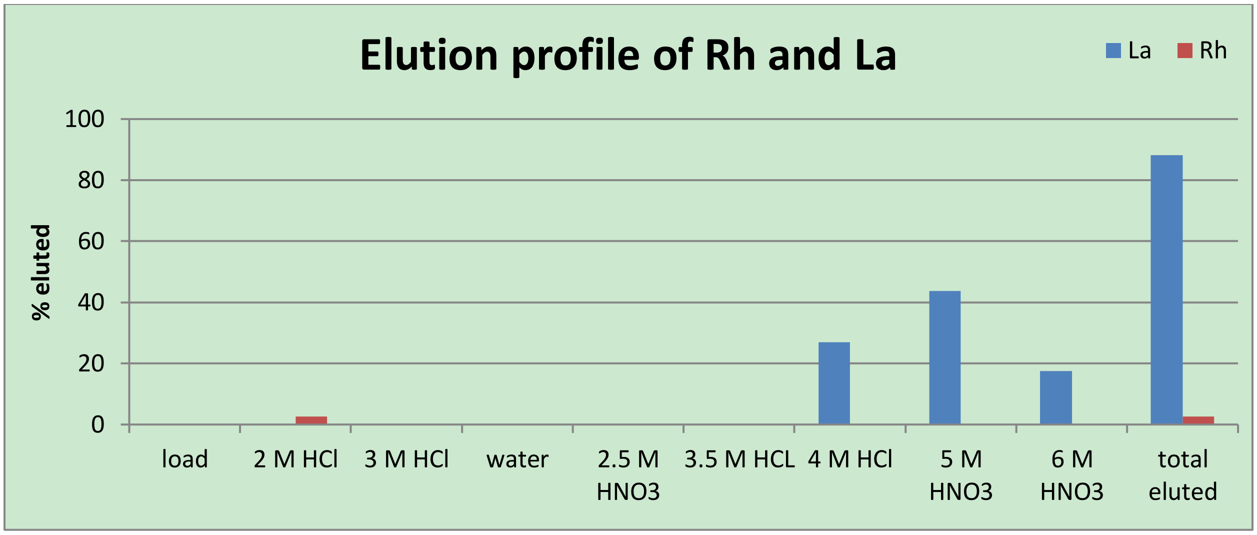

Figure s49. Elution profile of La and Ra with 3 BV of 2 M HCl, 3BV of 3 M HCl, 3BV of 18MΩ water, 3 BV of 2.5 M HNO_3, 10 BV of 3.5 M HCl, 10 BV of 4 M HCl, 10 BV of 5 M HNO_3 HNO_3, and 10 BV of 6 M HNO_3.

Mixed metal studies:

Large mixed metal studies with 55 elements: Used 150 µg of Ag, Al, As, B, Ba, Be, Bi, Ca, Cd, Ce, Co, Cr, Cu, Dy, Er, Eu, Fe, Ga, Gd, Ge, Ho, In, K, La, Li, Lu, Mg, Mn, Mo, Nb, Nd, Ni, Pb, Pr, Rb, Re, Rh, Ru, S, Sc, Se, Si, Sm, Sr, Ta, Tb, Th, Ti, Tl, Tm, U, V, W, Zn, Y, Yb, Zr.

<u>40+ element Study 1</u> Examine influence of initial acid on the retention of metals. Evaporated metal to a residue, added 25 mL of Conc HCl evaporated. Dissolved residue with 1 M Citric acid pH 2, loaded on AG50 column, rinsed with 3 BV of 1 M Citric acid, 3 BV of 2 M HCl, 3 BV of 3 M HCl, 3 BV of 3 M HCl, 3 BV of 2.5 M HNO_3, 3 BV of 2.5 M HNO3, 3 BV of 2.5 M HNO_3, 3 BV of 2.5 M HNO_3, 10 BV of 6 M NO_3, 10 BV of 6 M HNO_3. Repeated study with 25 ml of Coc Nitric acid.

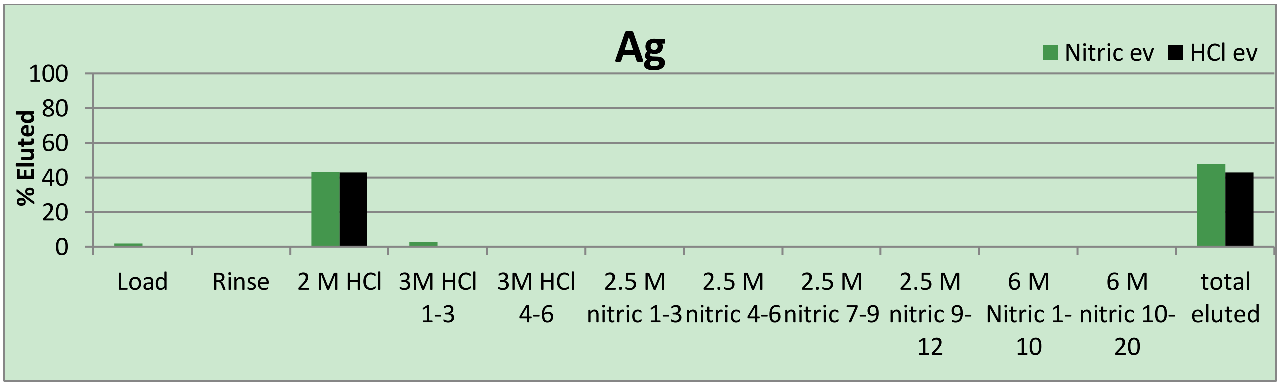

Figure s50. Elution profile of Ag.

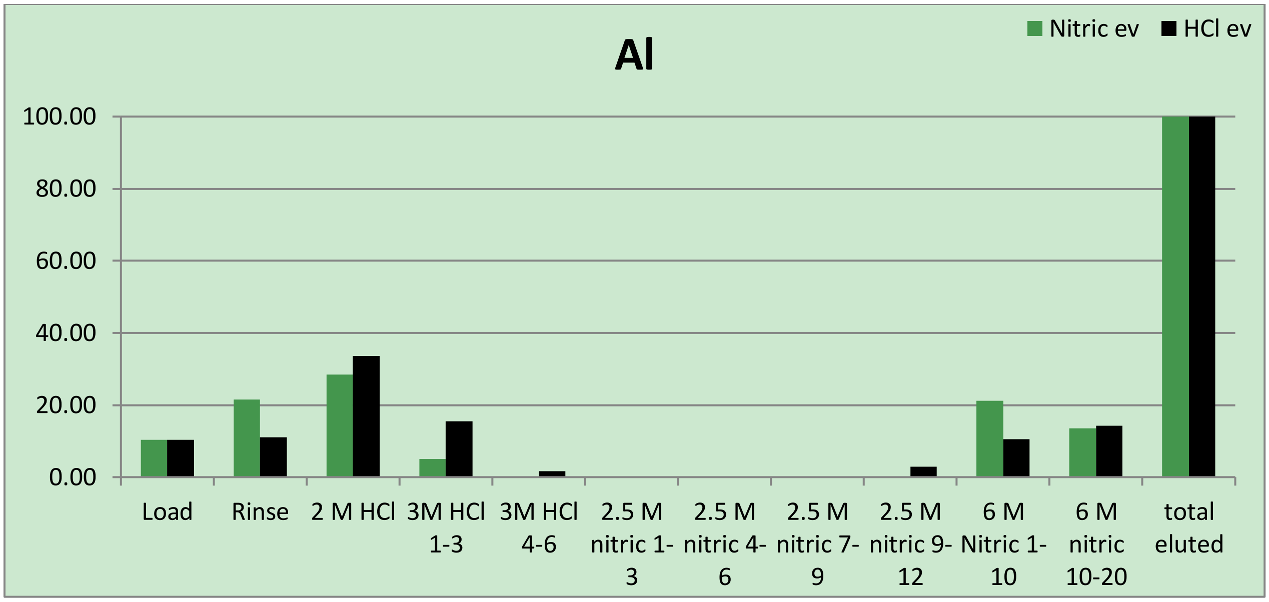

Figure s51. Elution profile of Al.

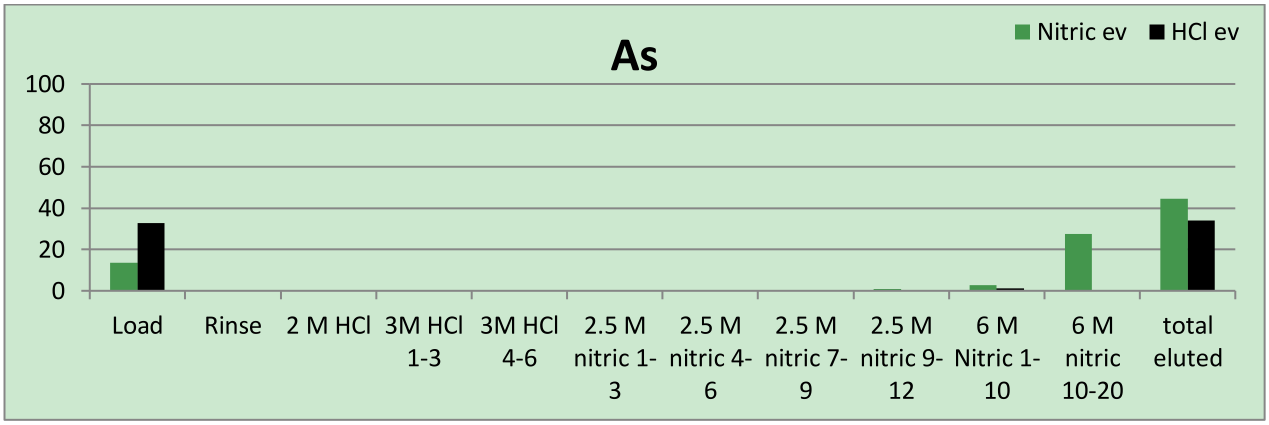

Figure s52. Elution profile of Ag.

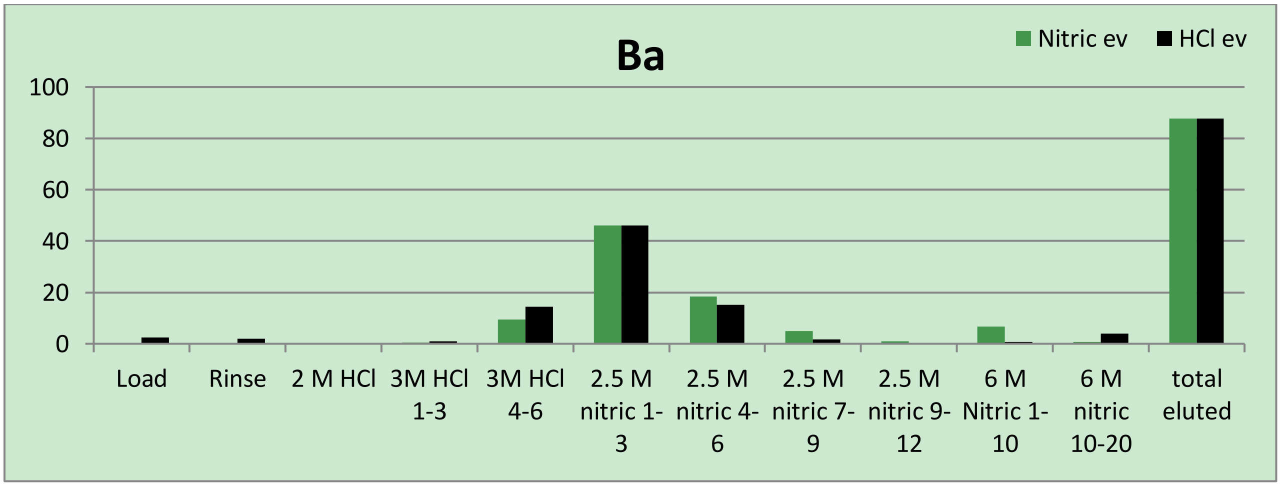

Figure s53. Elution profile of Ba.

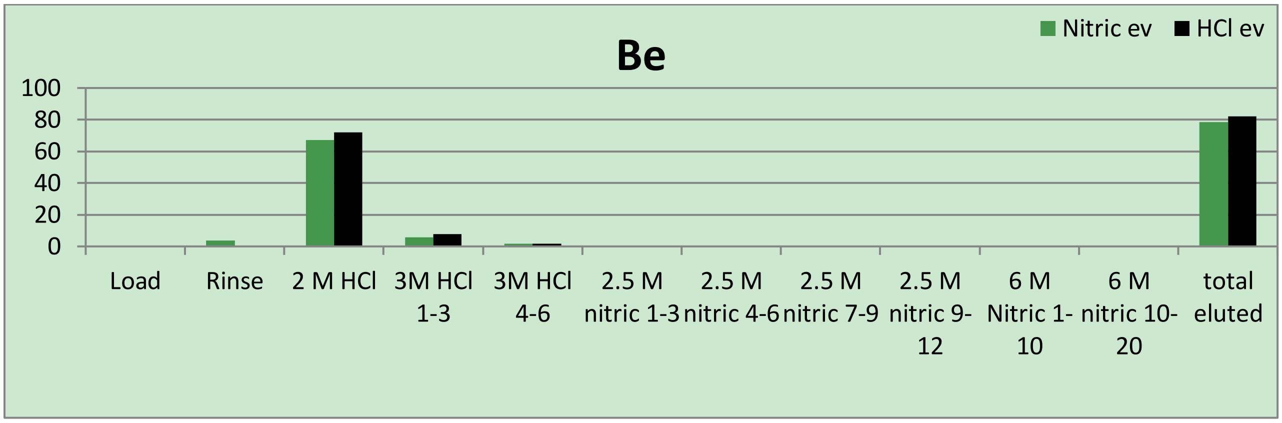

Figure s54. Elution profile of Be.

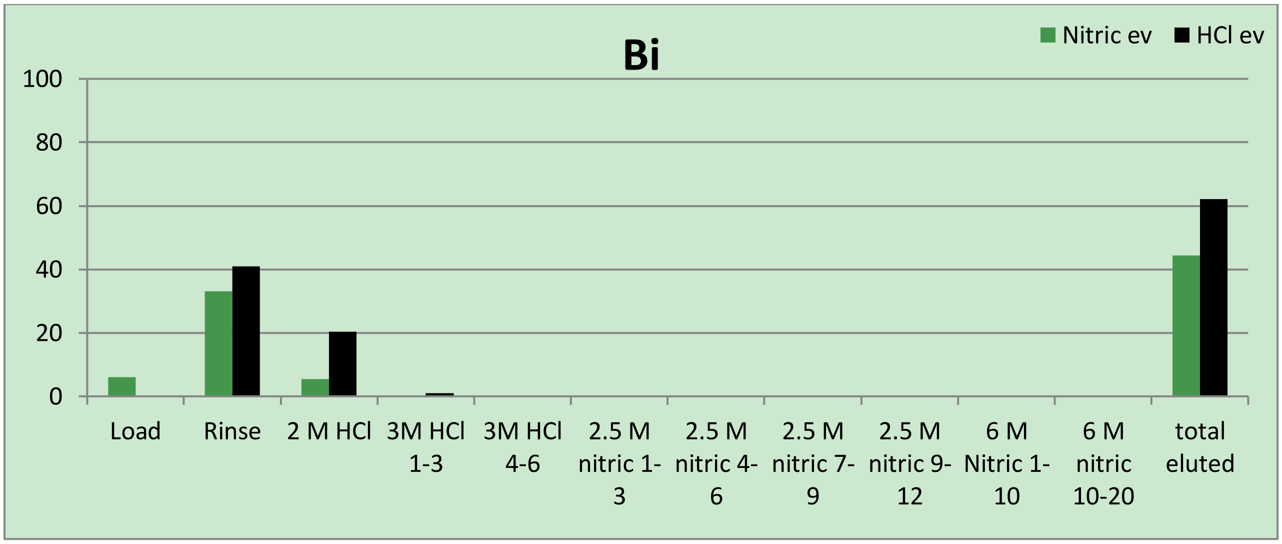

Figure s55. Elution profile of Bi.

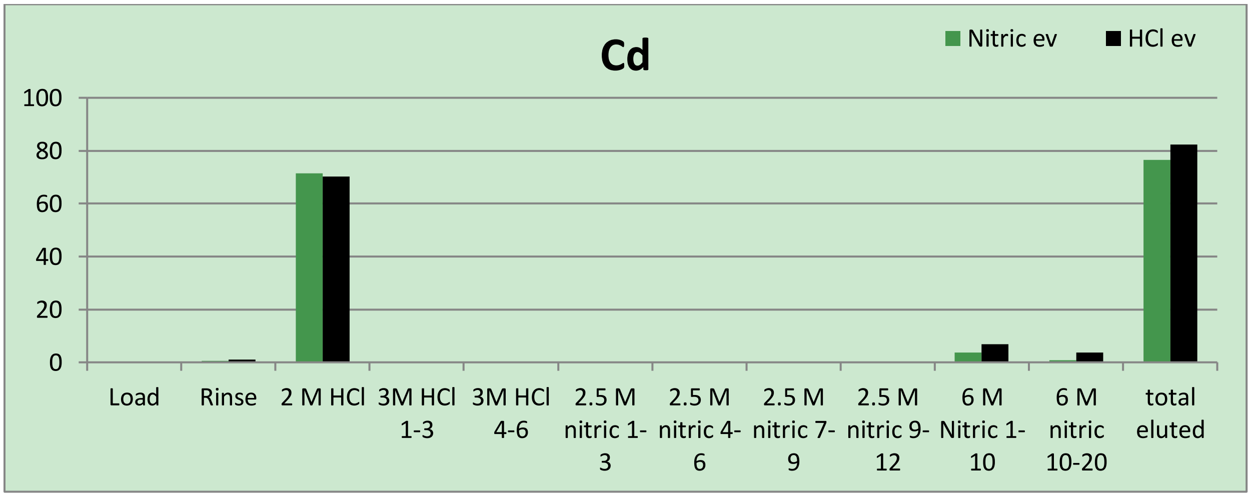

Figure s56. Elution profile of Cd.

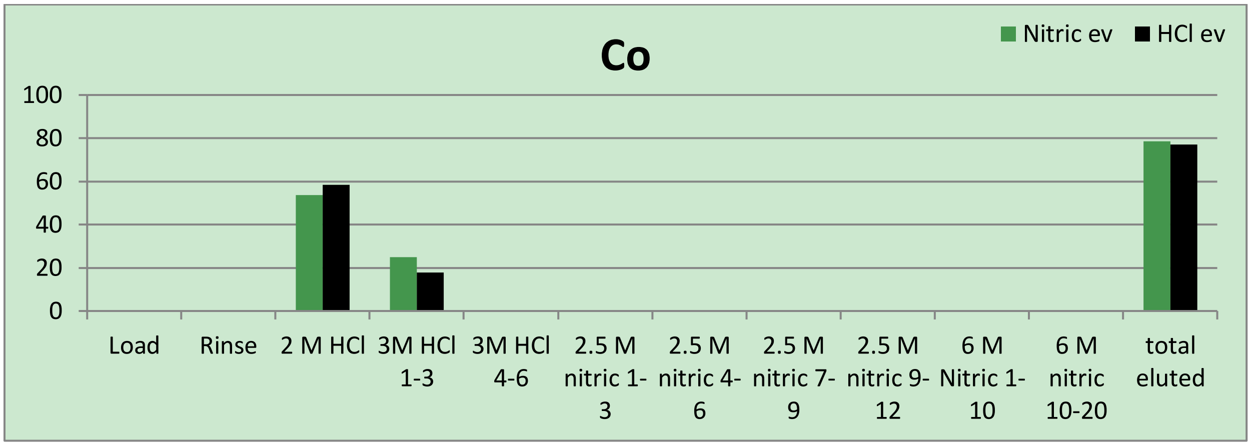

Figure s57. Elution profile of Co.

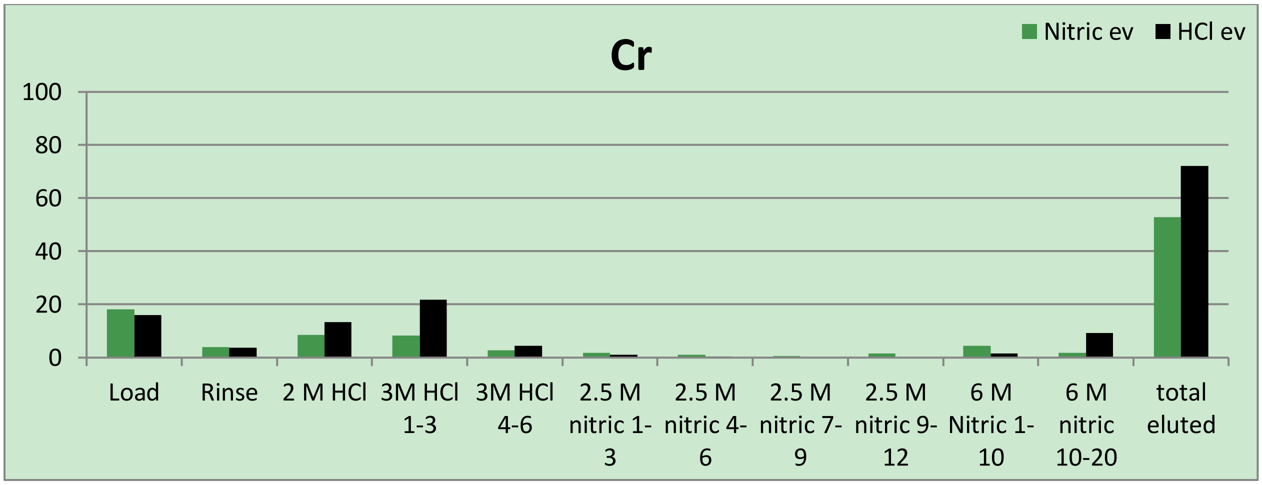

Figure s58. Elution profile of Cr.

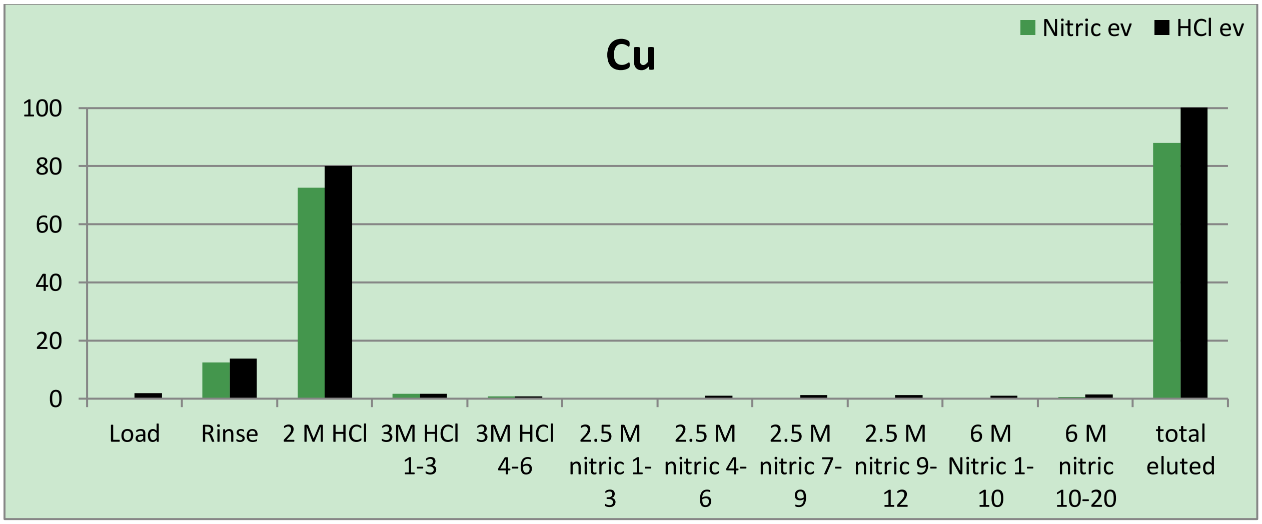

Figure s59. Elution profile of Cu.

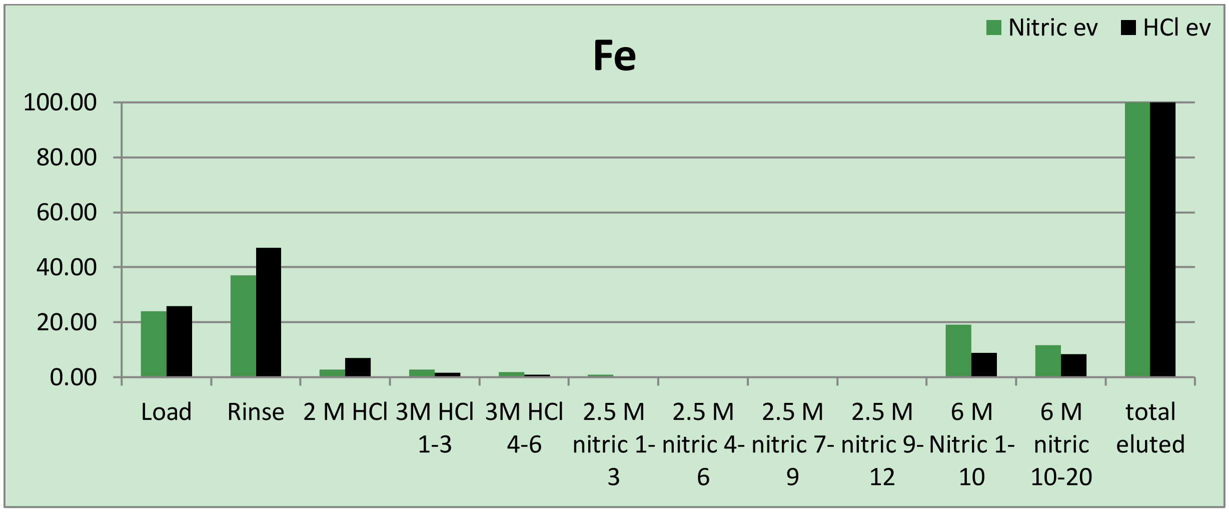

Figure s60. Elution profile of Fe.

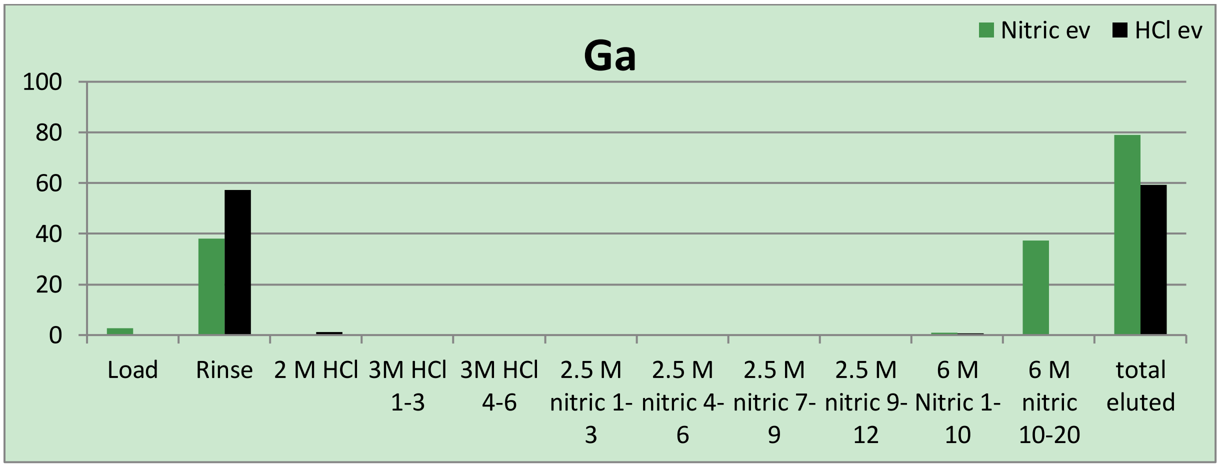

Figure s61. Elution profile of Ge.

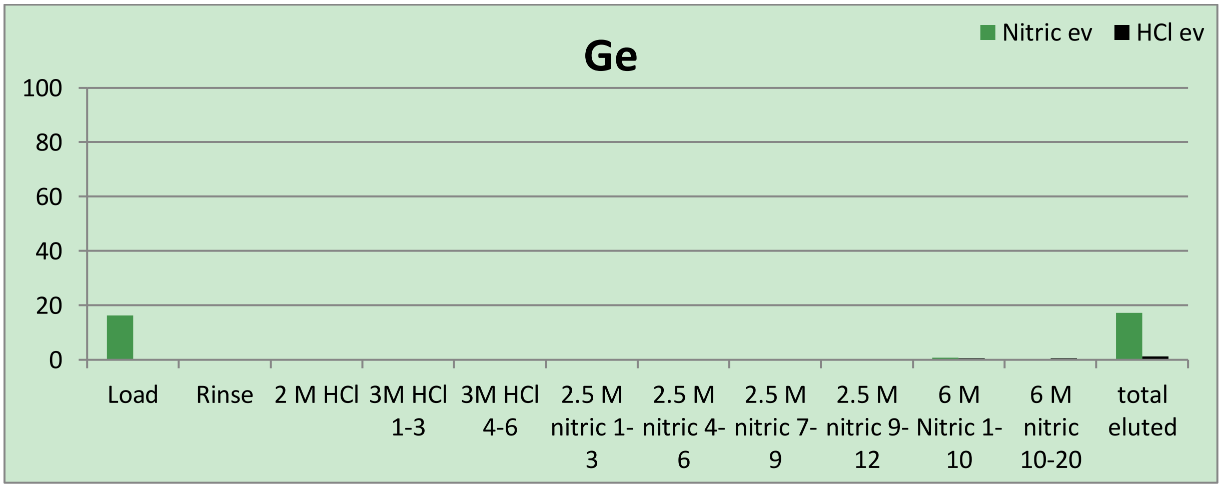

Figure s62. Elution profile of Ge.

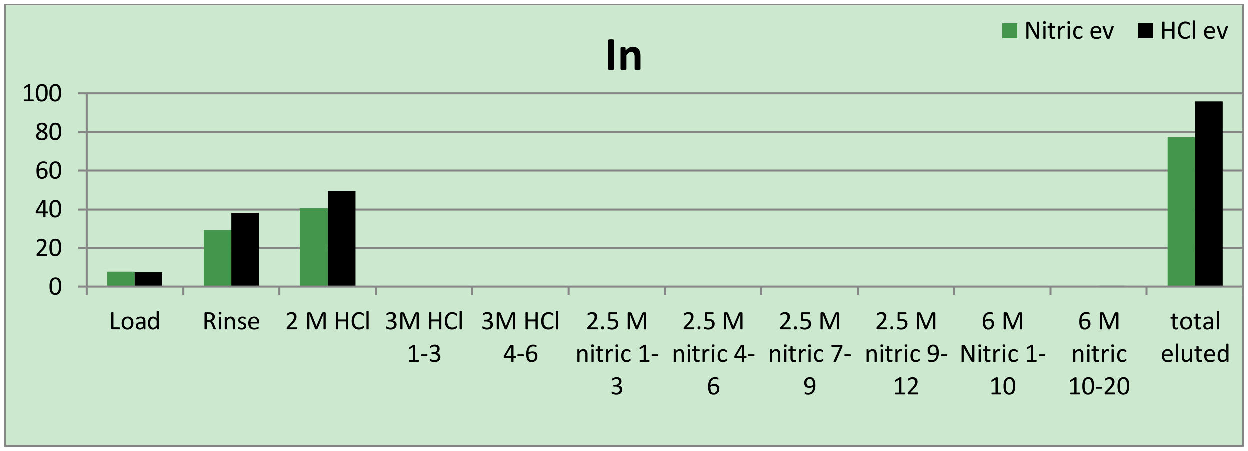

Figure s63. Elution profile of In.

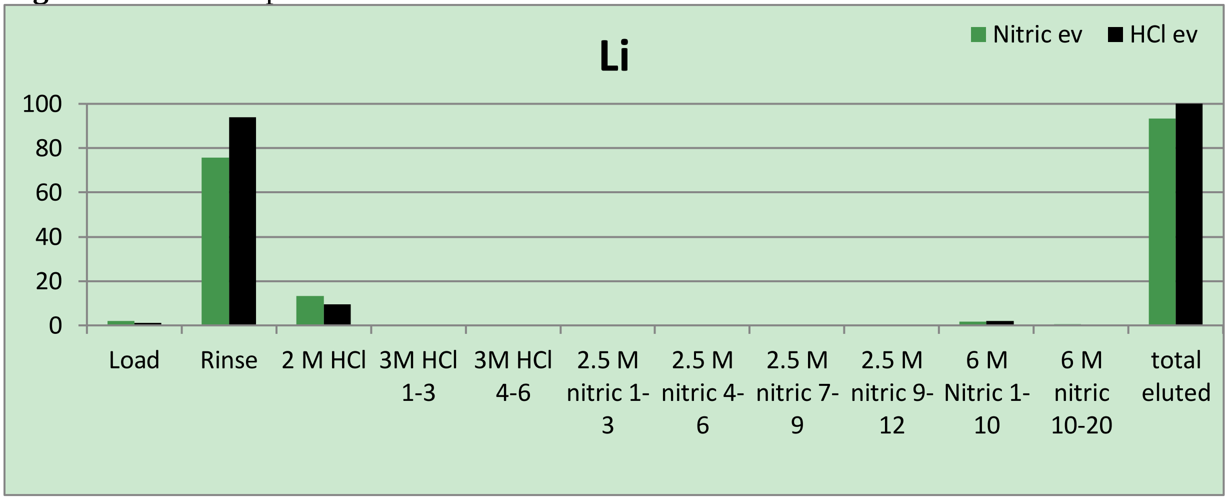

Figure s64. Elution profile of Li.

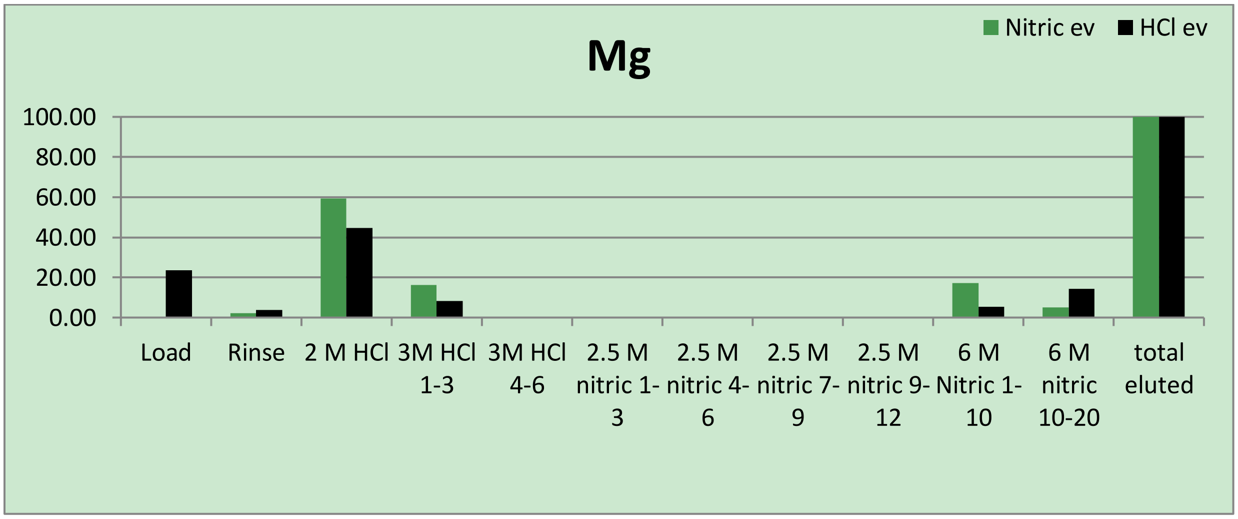

Figure s65. Elution profile of Mg.

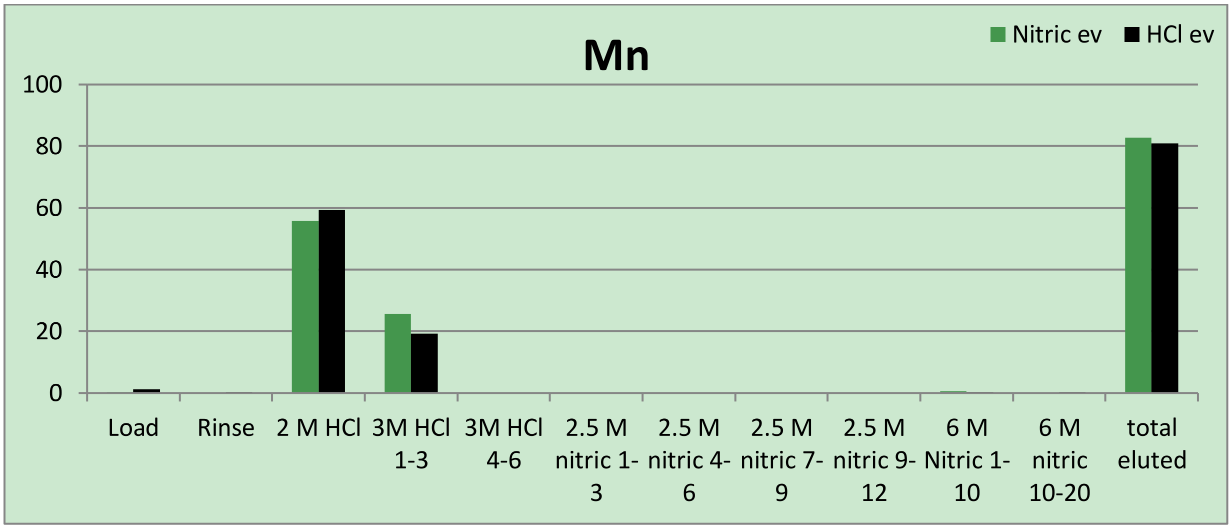

Figure s66. Elution profile of Mn.

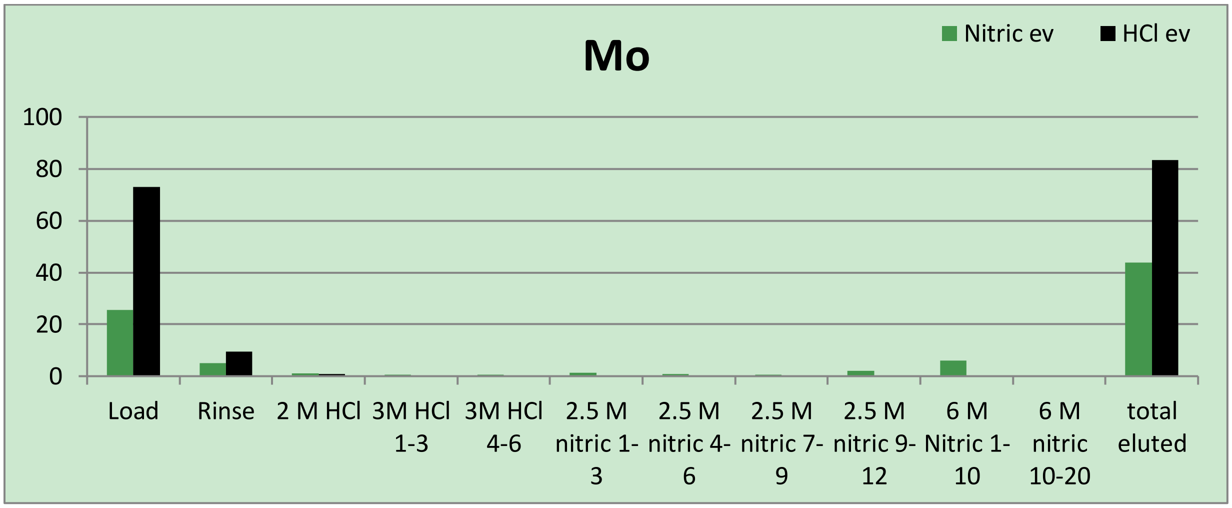

Figure s67. Elution profile of Mo.

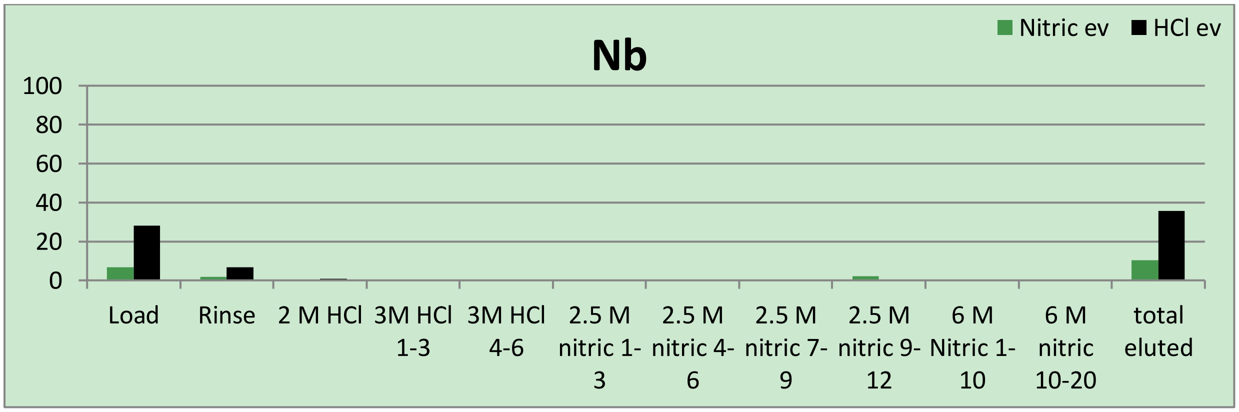

Figure s68. Elution profile of Nb.

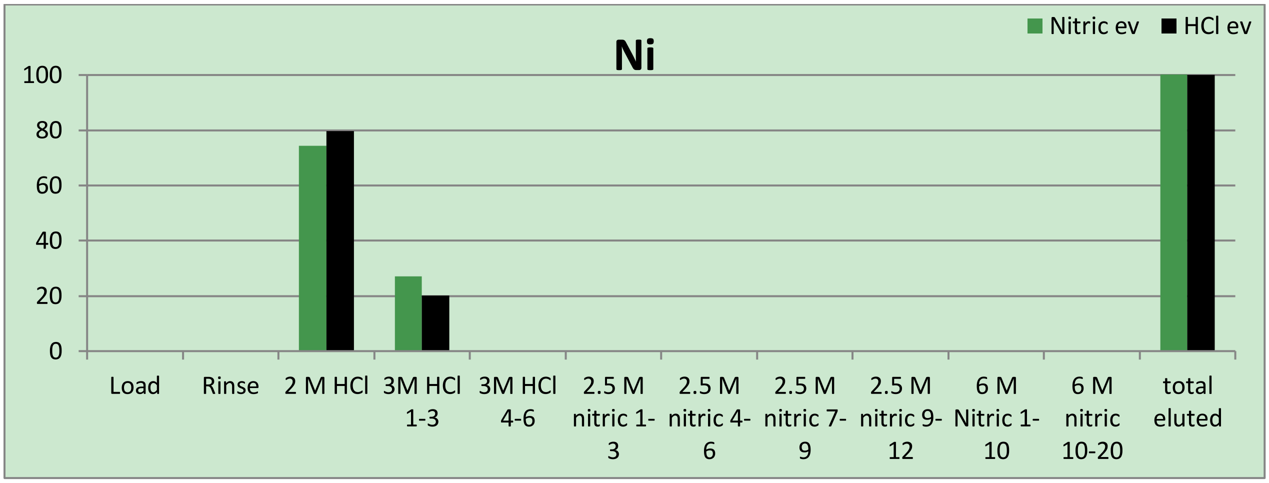

Figure s69. Elution profile of Ni.

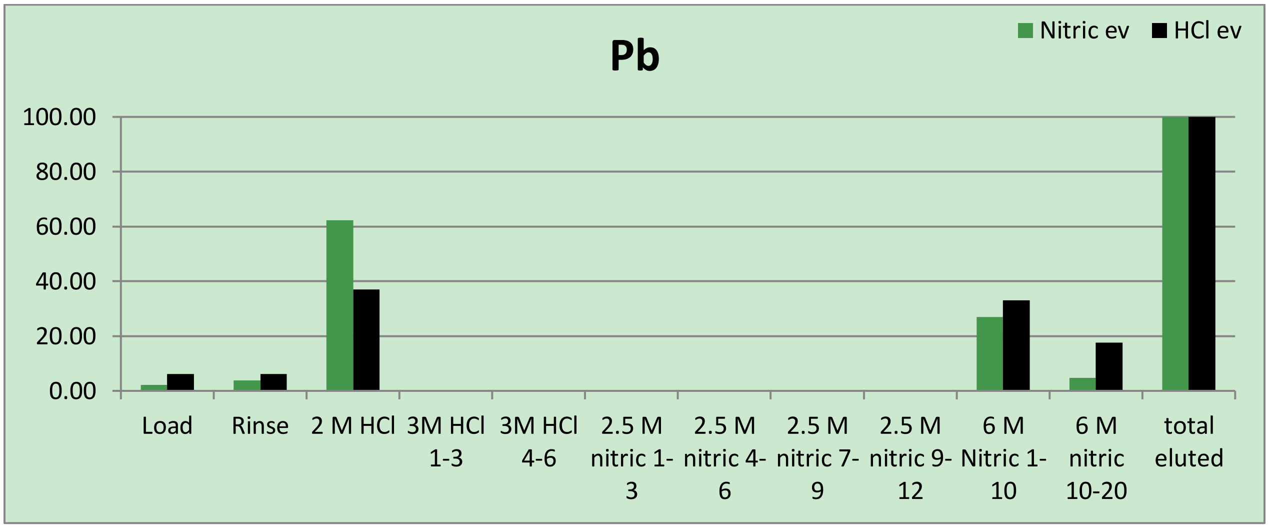

Figure s70. Elution profile of Pb.

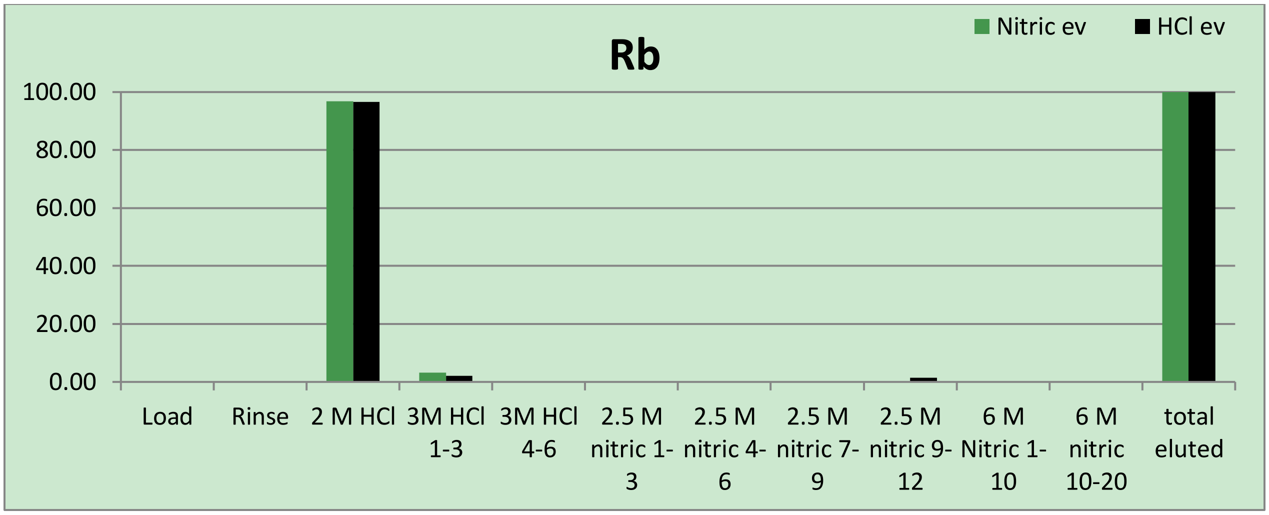

Figure s71. Elution profile of Rb.

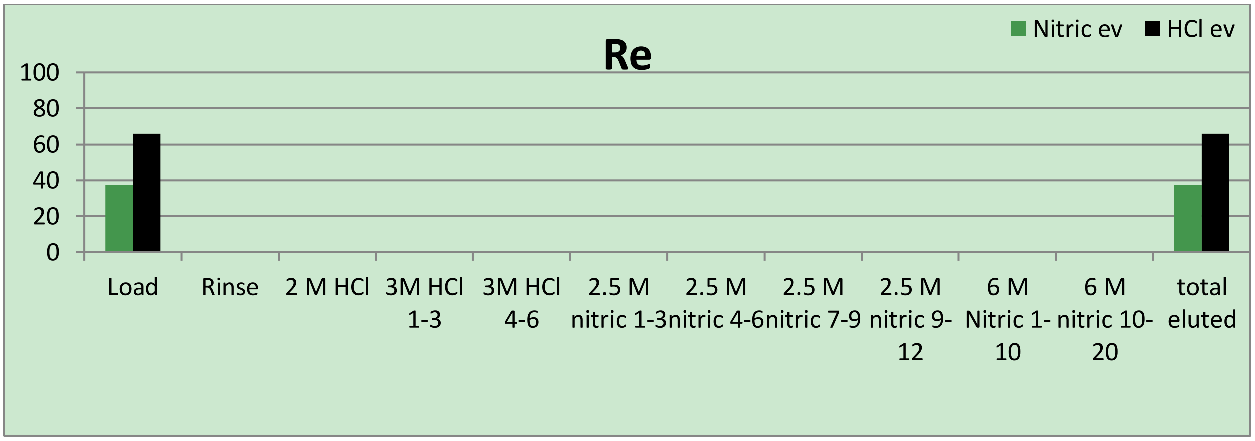

Figure s72. Elution profile of Re.

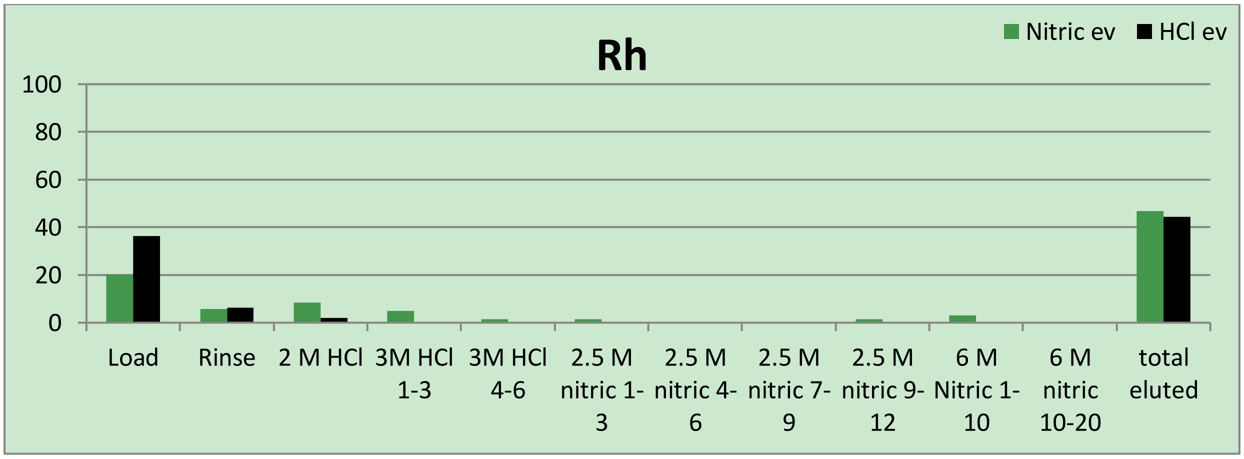

Figure s73. Elution profile of Rh.

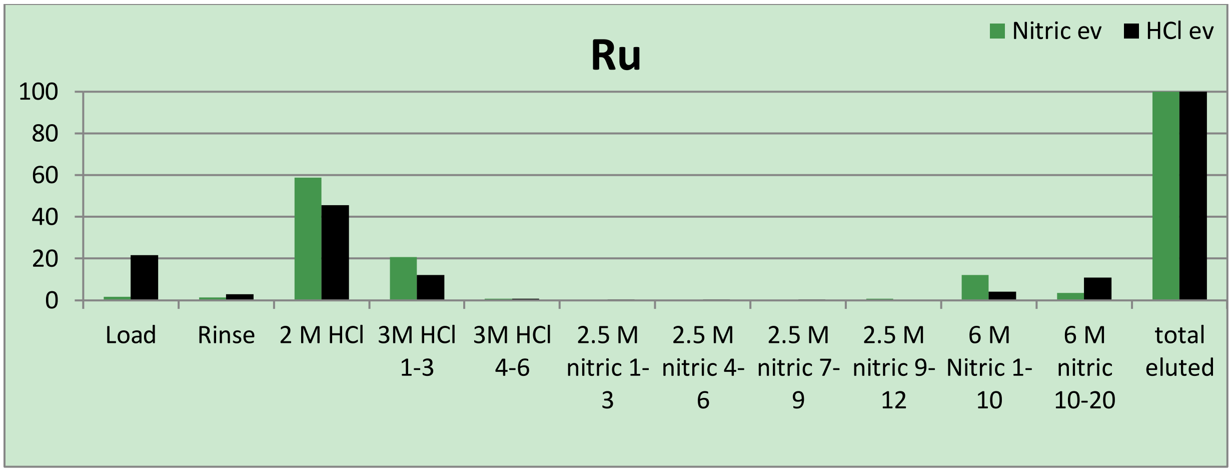

Figure s74. Elution profile of Ru.

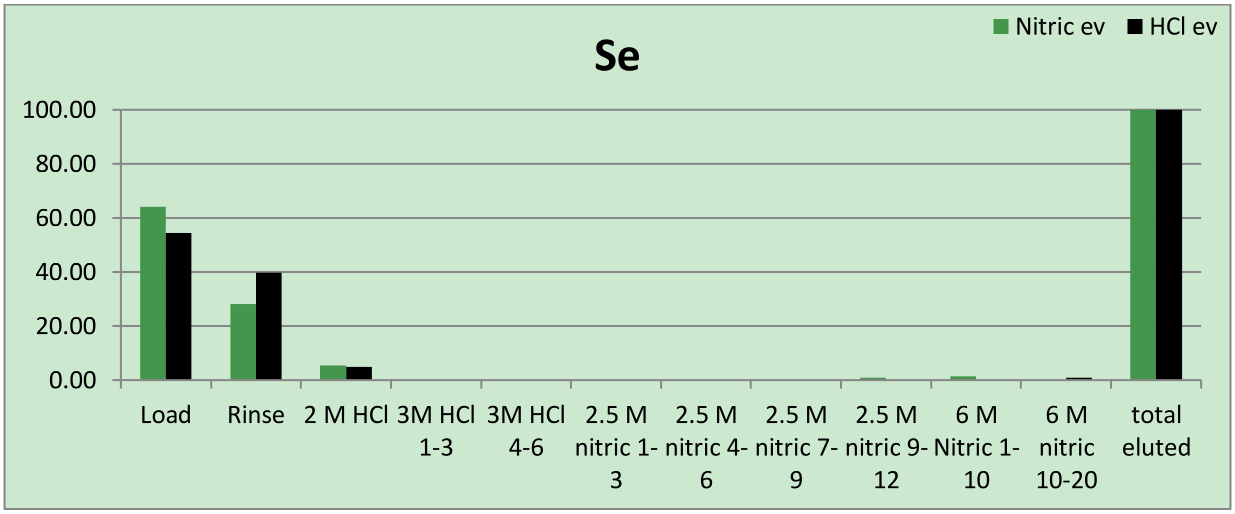

Figure s75. Elution profile of Se.

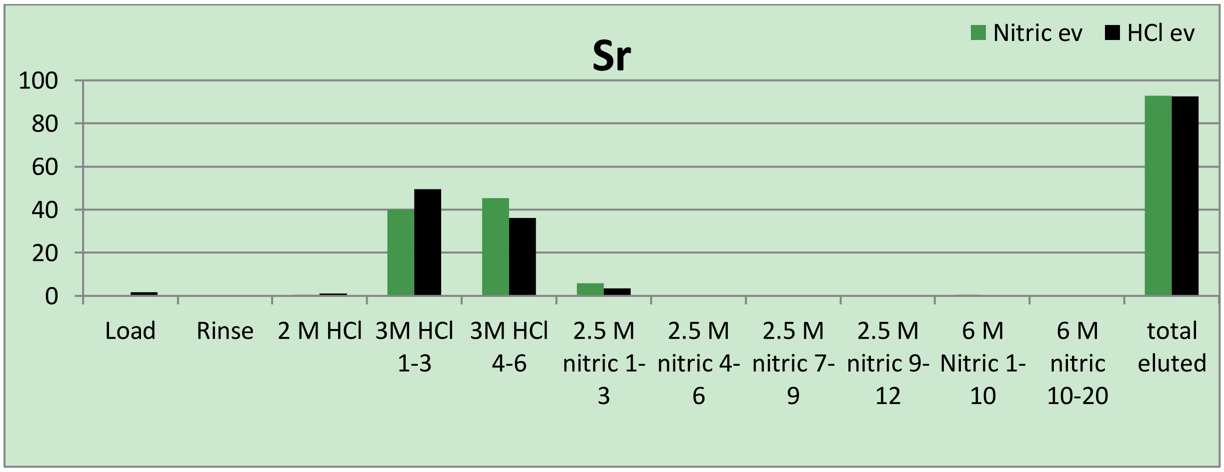

Figure s76. Elution profile of Sr.

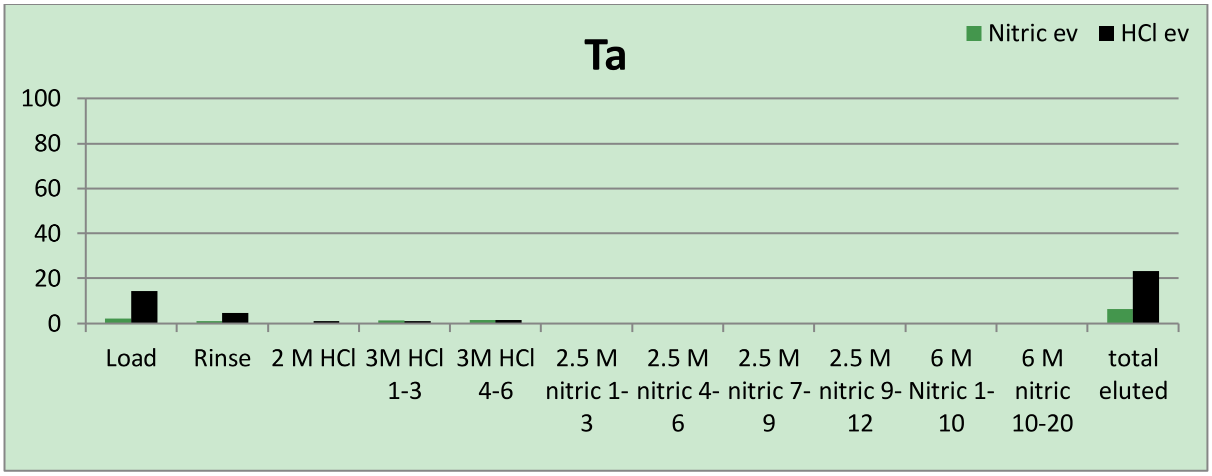

Figure s77. Elution profile of Ta.

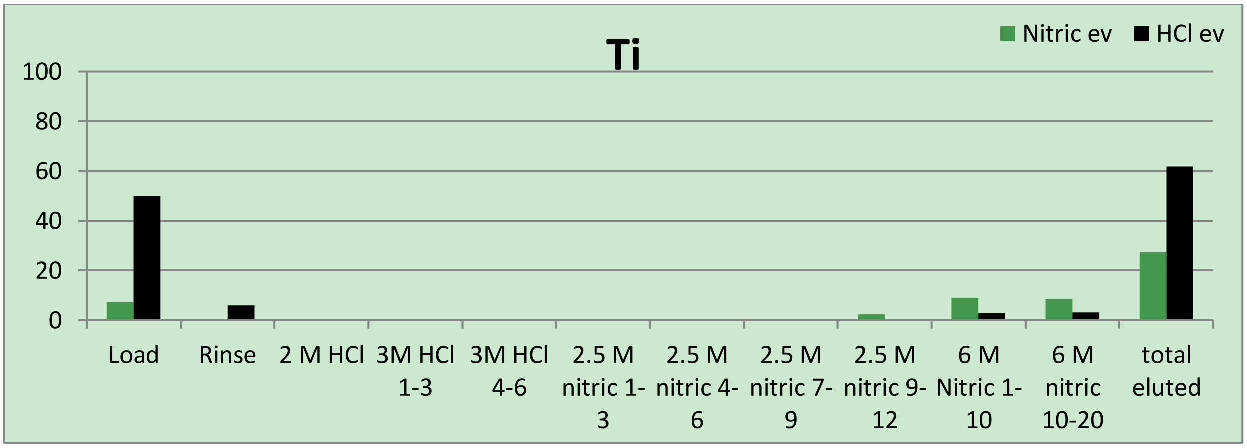

Figure s78. Elution profile of Ti.

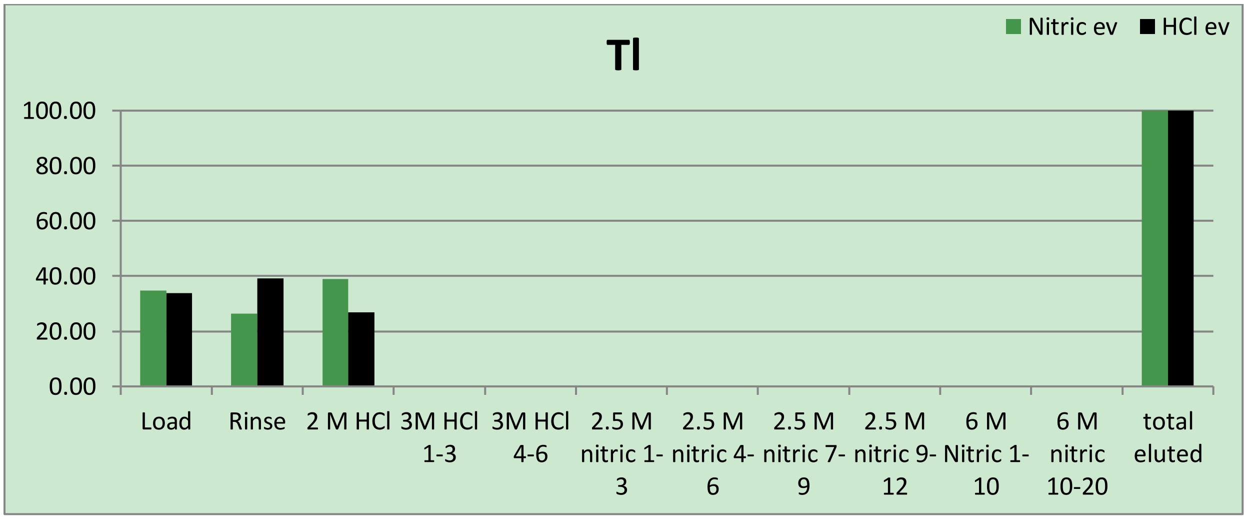

Figure s79. Elution profile of Tl.

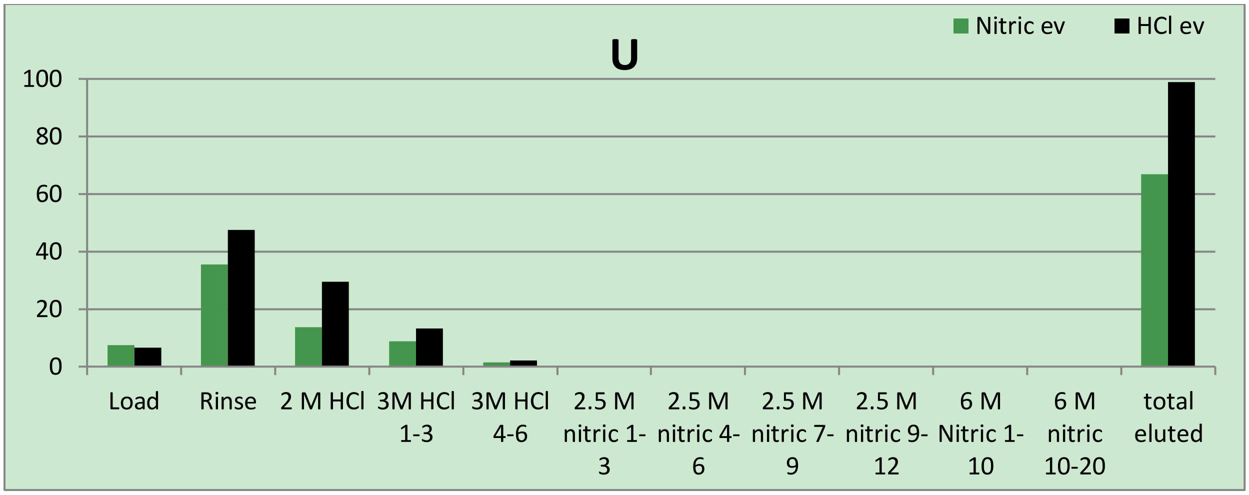

Figure s80. Elution profile of U.

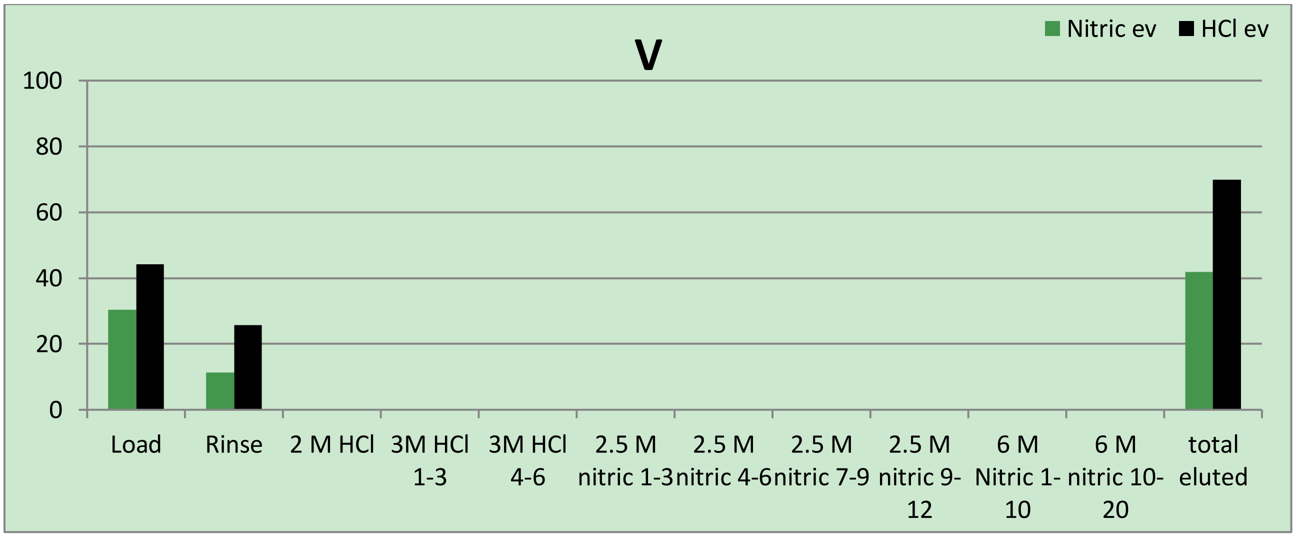

Figure s81. Elution profile of V.

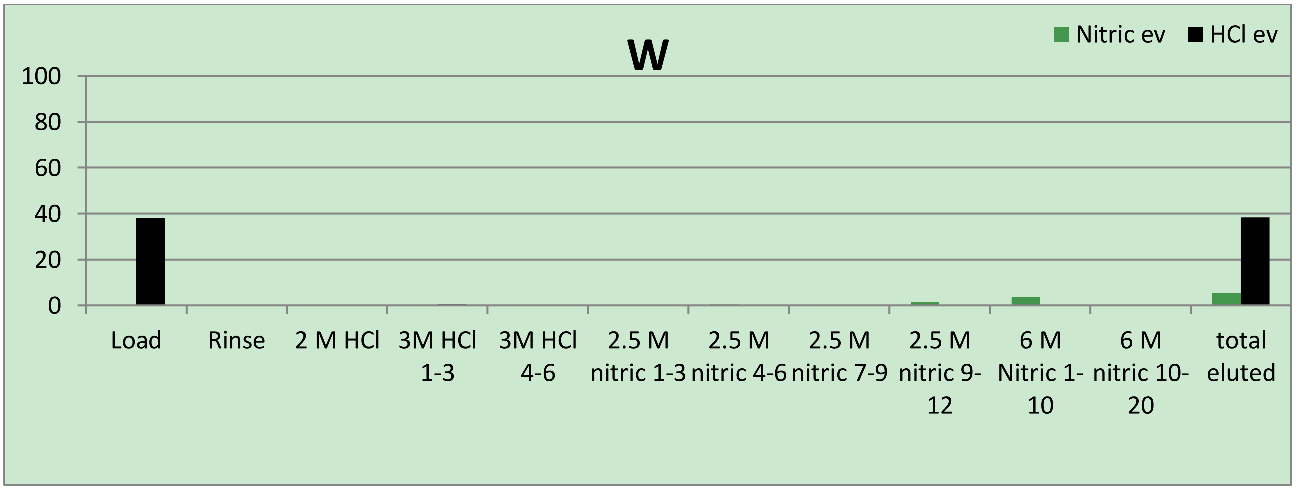

Figure s82. Elution profile of W.

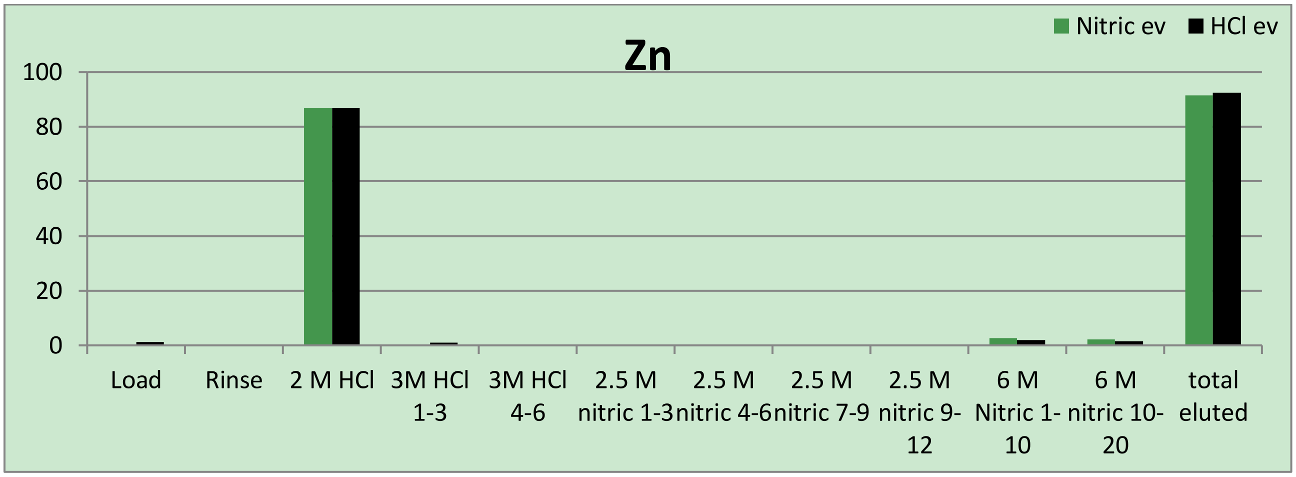

Figure s83. Elution profile of Zn.

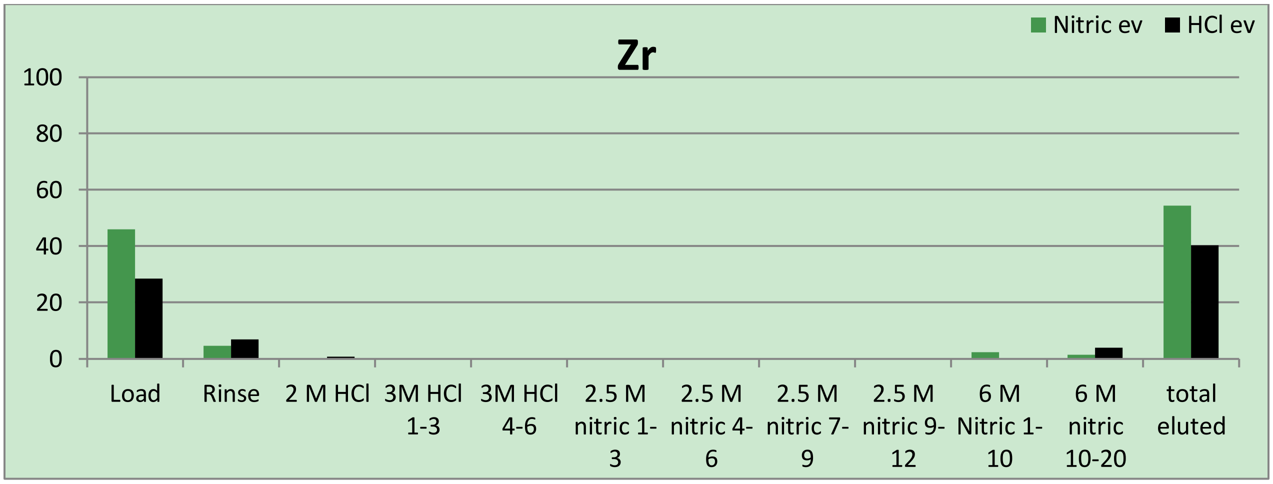

Figure s84. Elution profile of Zr.

Lanthanides and Th

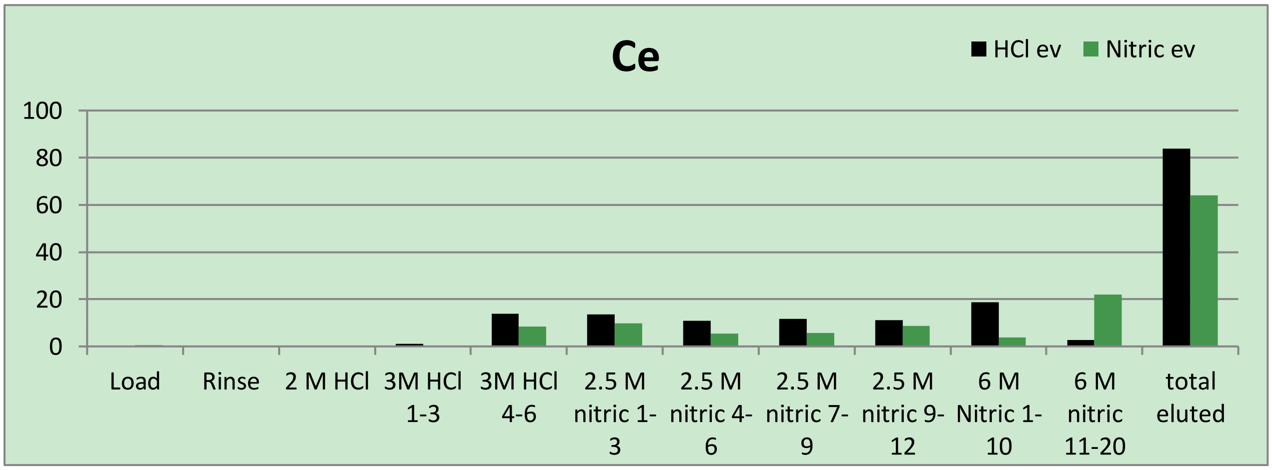

Figure s85. Elution profile of Ce.

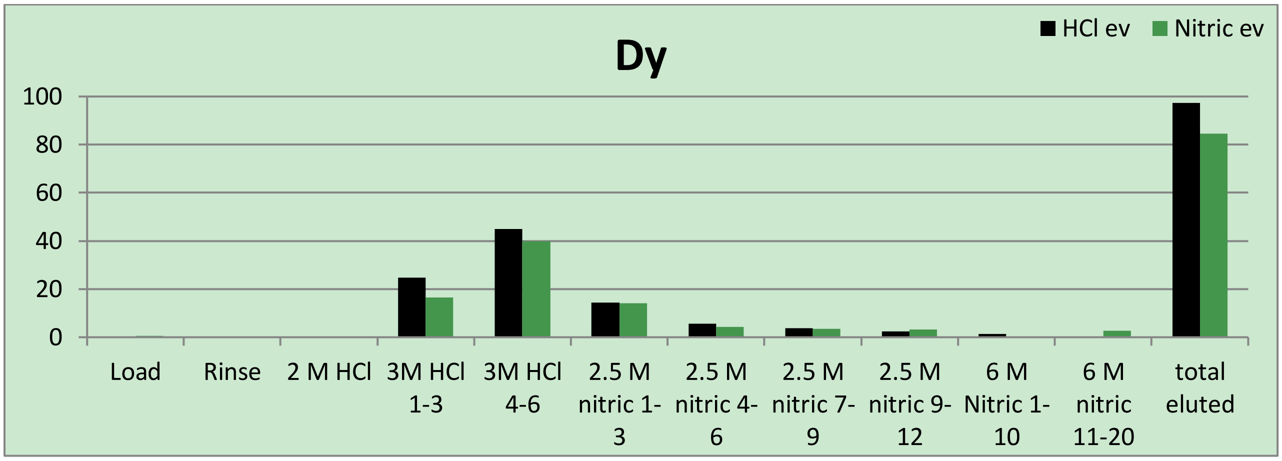

Figure s86. Elution profile of Dy.

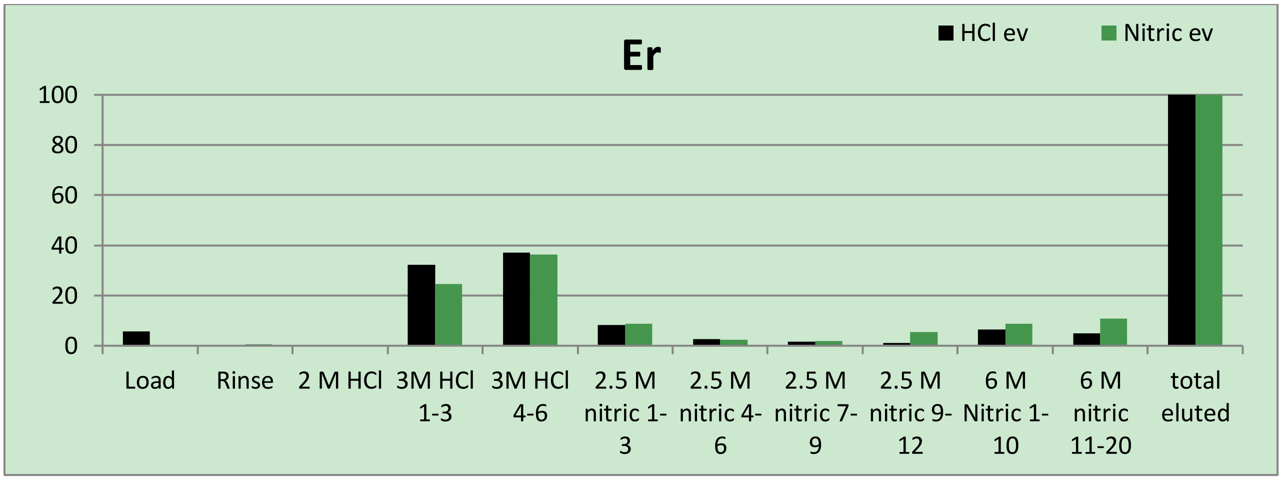

Figure s87. Elution profile of Er.

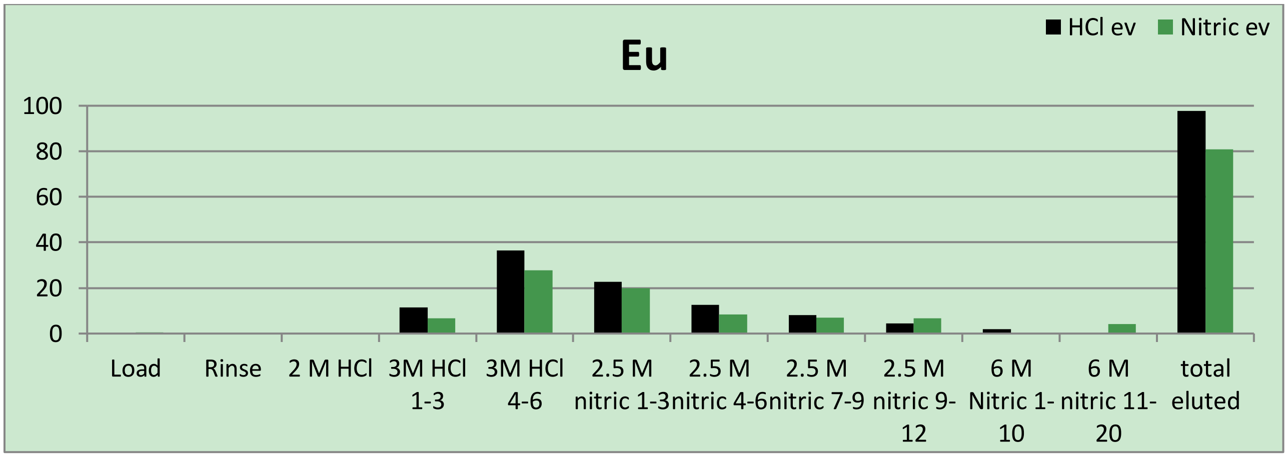

Figure s88. Elution profile of Eu.

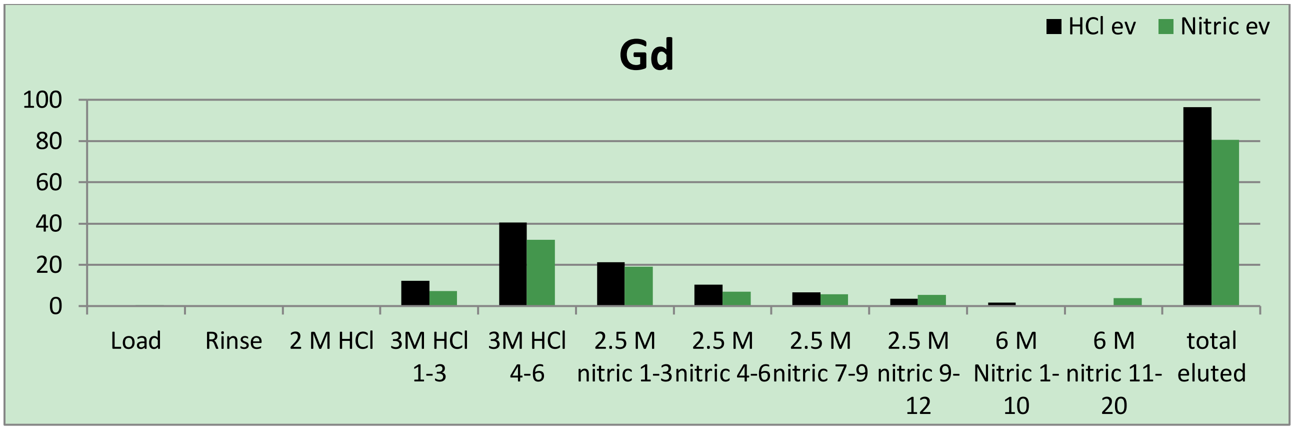

Figure s89. Elution profile of Gd.

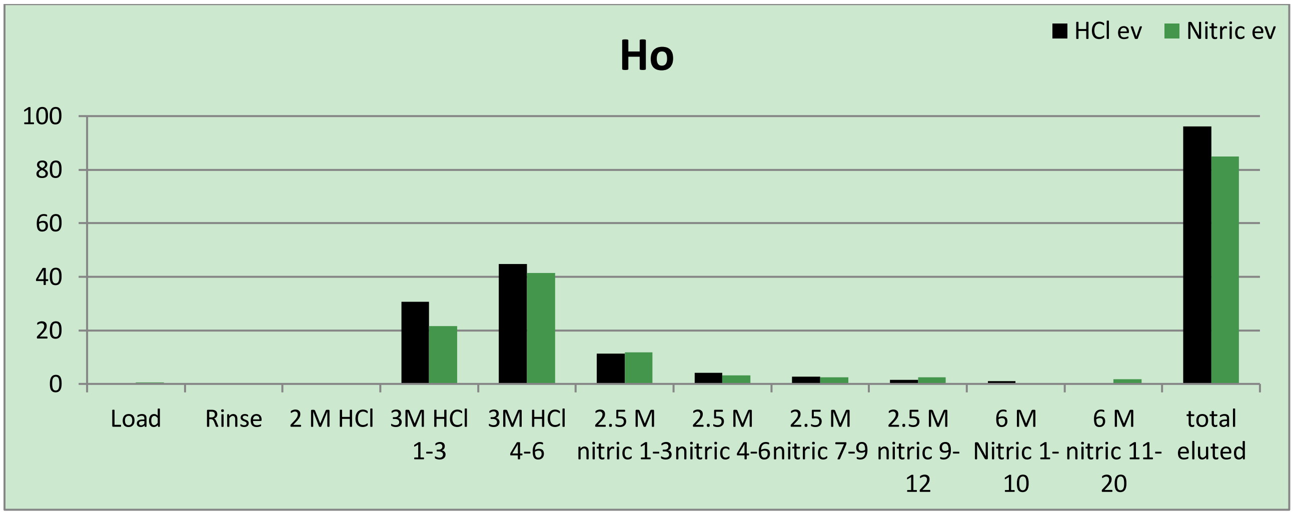

Figure s90. Elution profile of Ho.

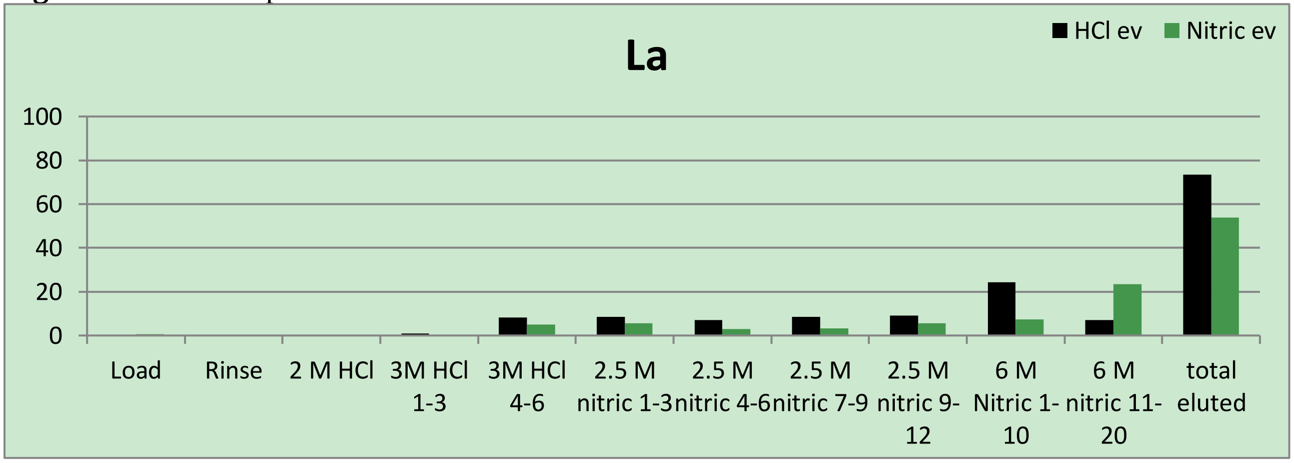

Figure s91. Elution profile of La.

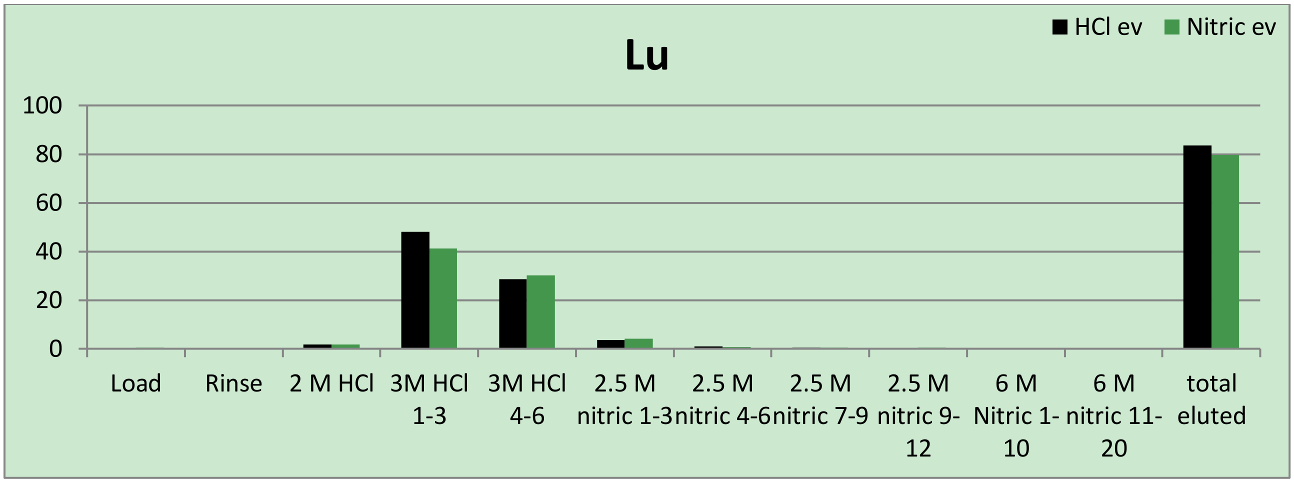

Figure s92. Elution profile of Lu.

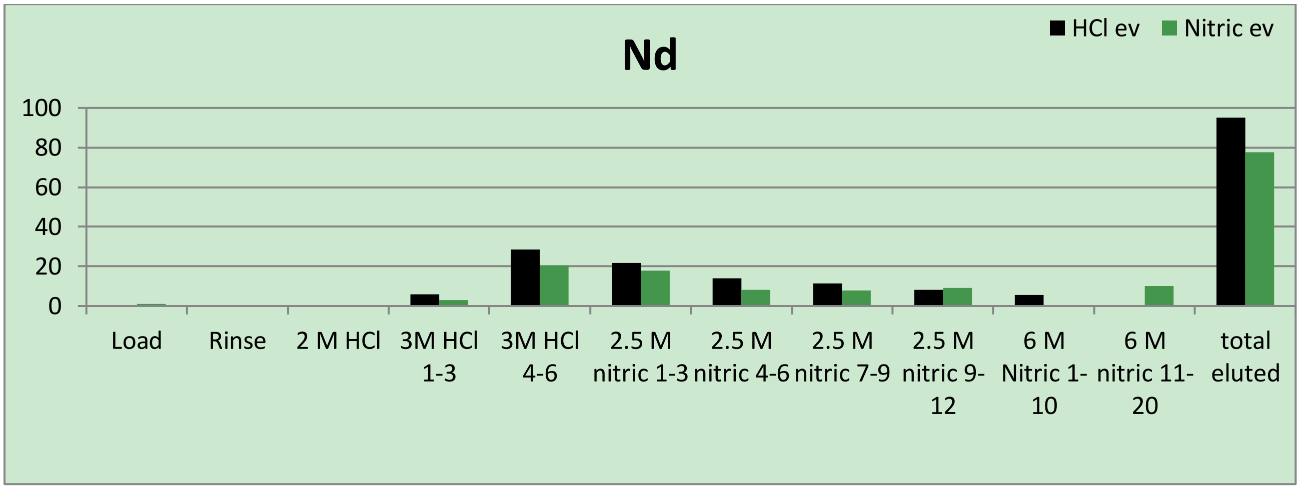

Figure s93. Elution profile of Nd.

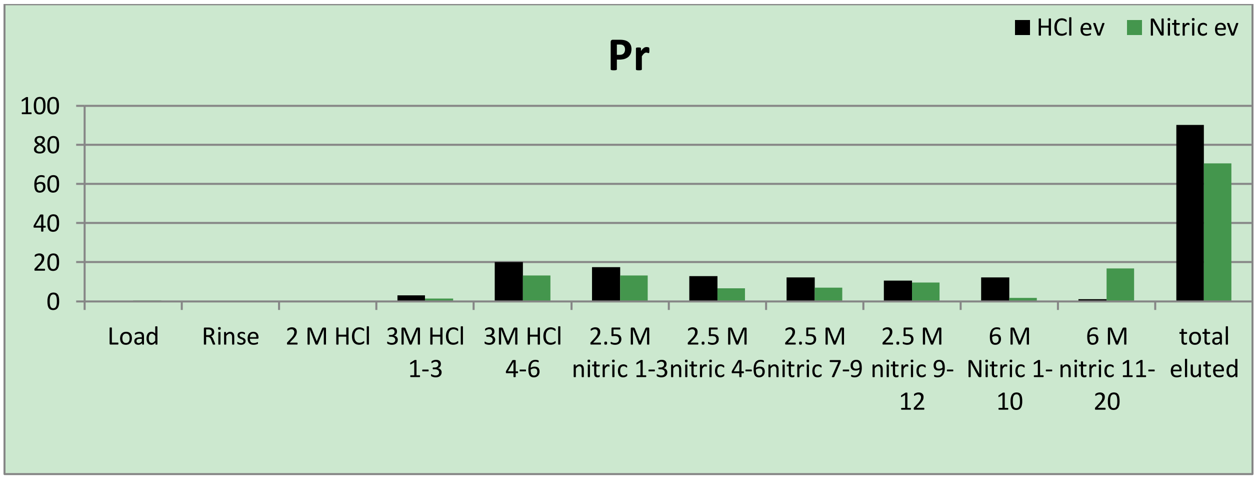

Figure s94. Elution profile of Pr.

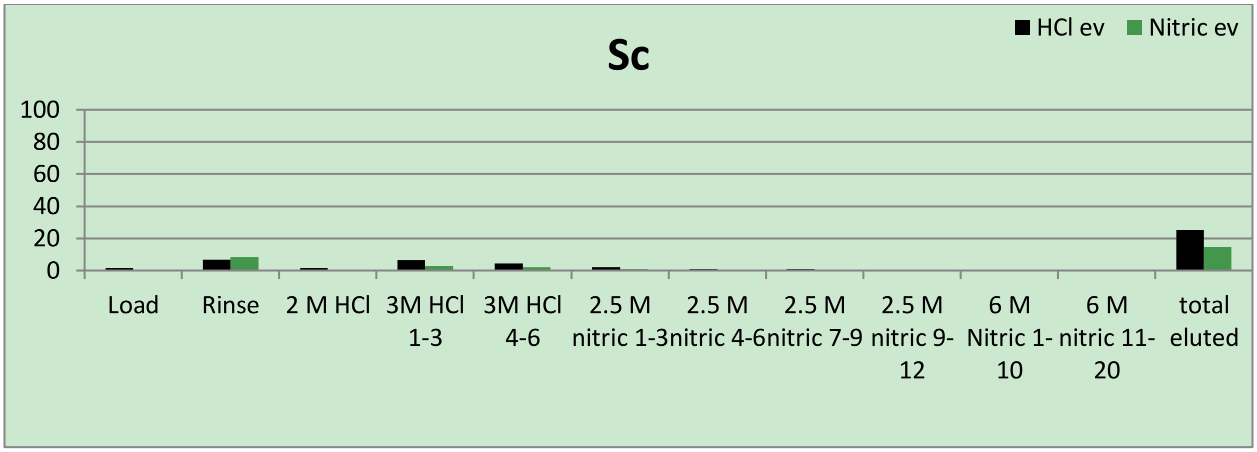

Figure s95. Elution profile of Sc.

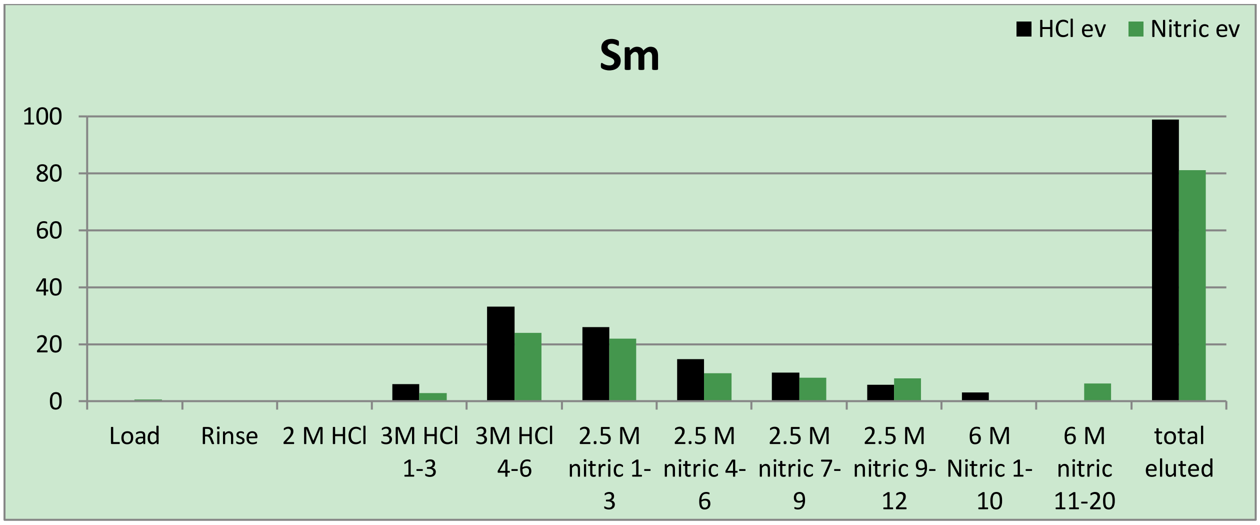

Figure s96. Elution profile of Sm.

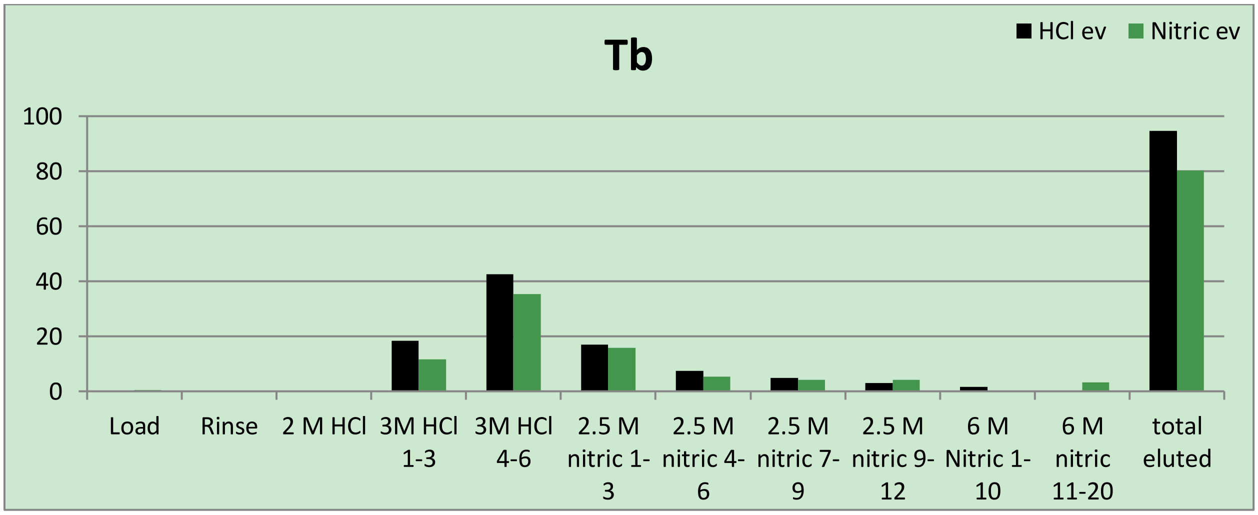

Figure s97. Elution profile of Tb.

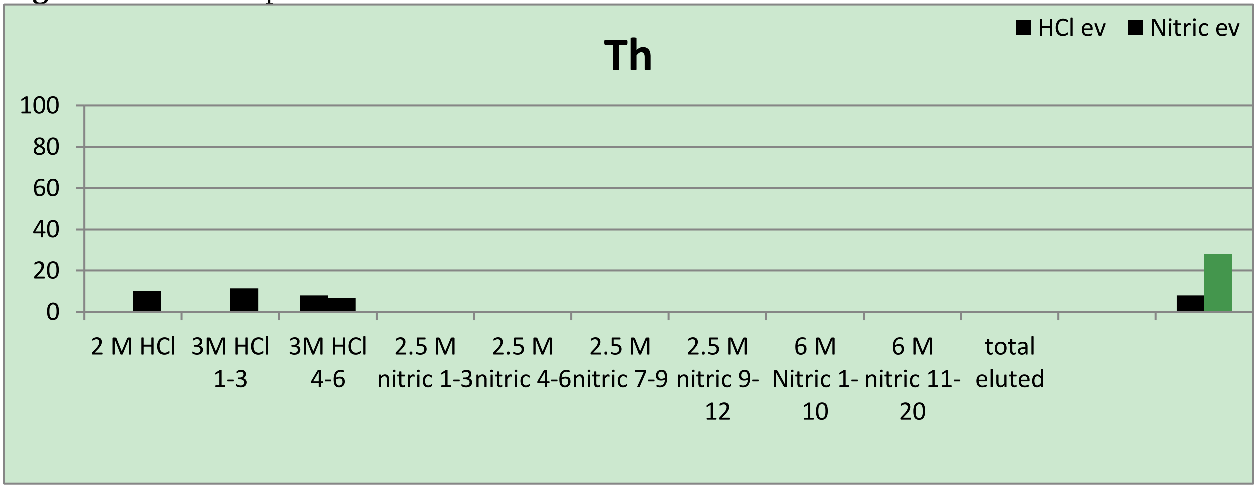

Figure s98. Elution profile of Th.

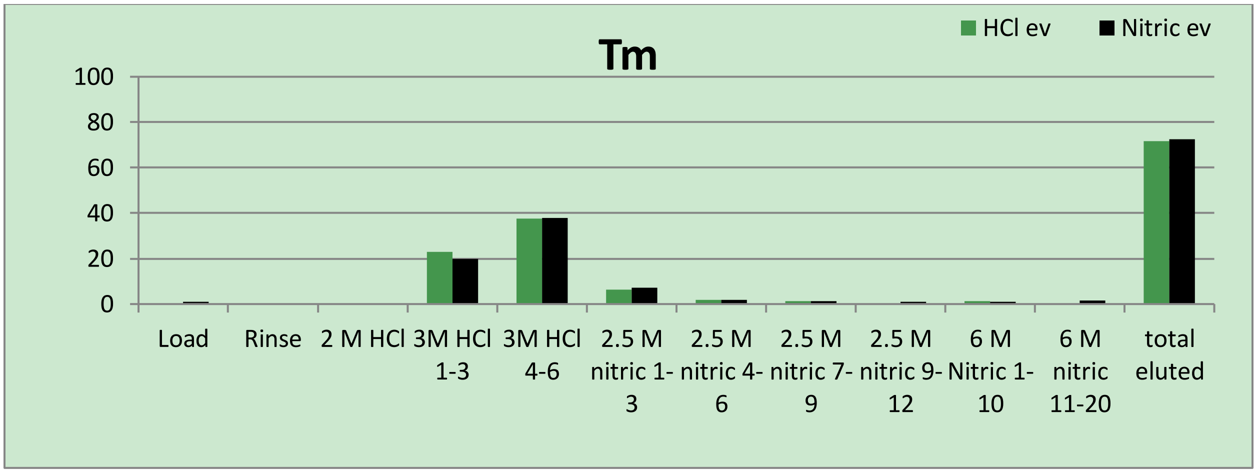

Figure s99. Elution profile of Tm.

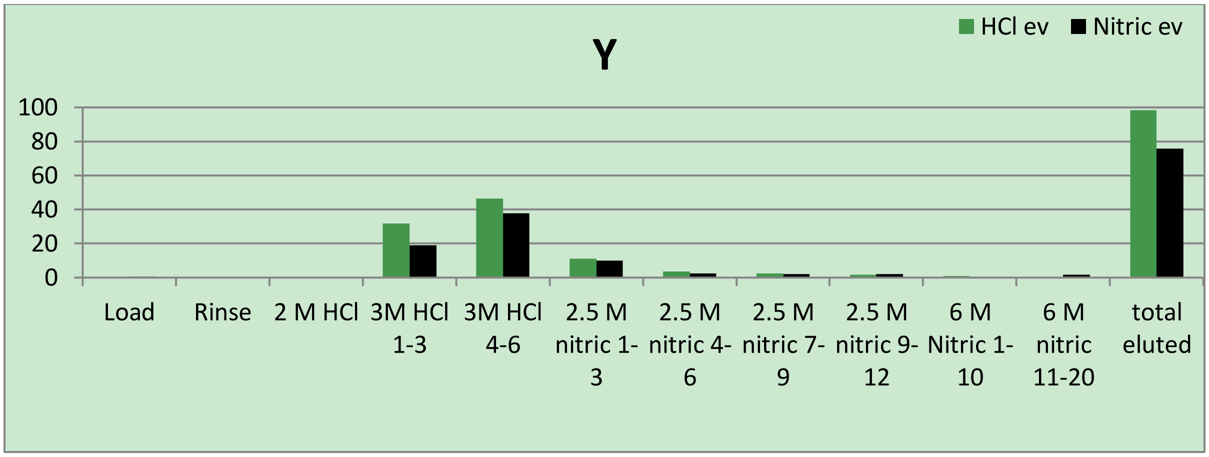

Figure s100. Elution profile of Y.

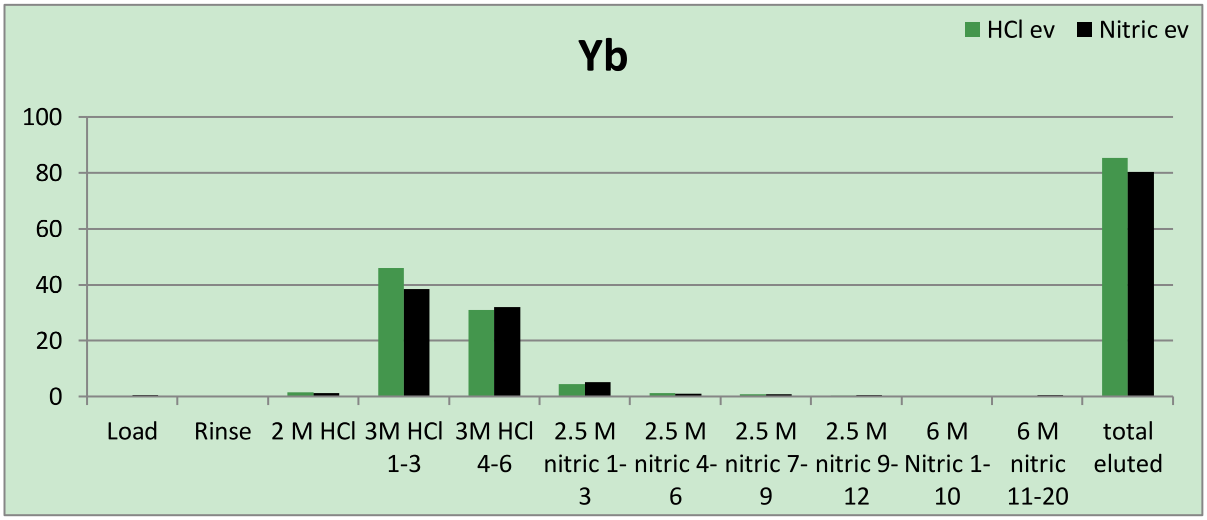

Figure s101. Elution profile of Yb.

Large mixed metal studies with 55 elements: Used 150 µg of Ag, Al, As, B, Ba, Be, Bi, Ca, Cd, Ce, Co, Cr, Cu, Dy, Er, Eu, Fe, Ga, Gd, Ge, Ho, In, K, La, Li, Lu, Mg, Mn, Mo, Nb, Nd, Ni, Pb, Pr, Rb, Re, Rh, Ru, S, Sc, Se, Si, Sm, Sr, Ta, Tb, Th, Ti, Tl, Tm, U, V, W, Zn, Y, Yb, Zr.

55 element Study 2 Changed elution profile

Evaporated metal, added 25 mL of Nitric acid evaporated. Dissolved residue with 1 M Citric acid pH 2, loaded on AG50 column, rinsed with 3 BV of 1 M Citric acid, 3 BV of 2 M HCl, 3 BV of 3 M HCl, 3 BV of 2.5 M HNO_3, 3 BV of 2.5 M HNO3, 3 BV of 2.5 M HNO_3, 10 BV of 6 M NO_3, 10 BV of 6 M HNO_3.

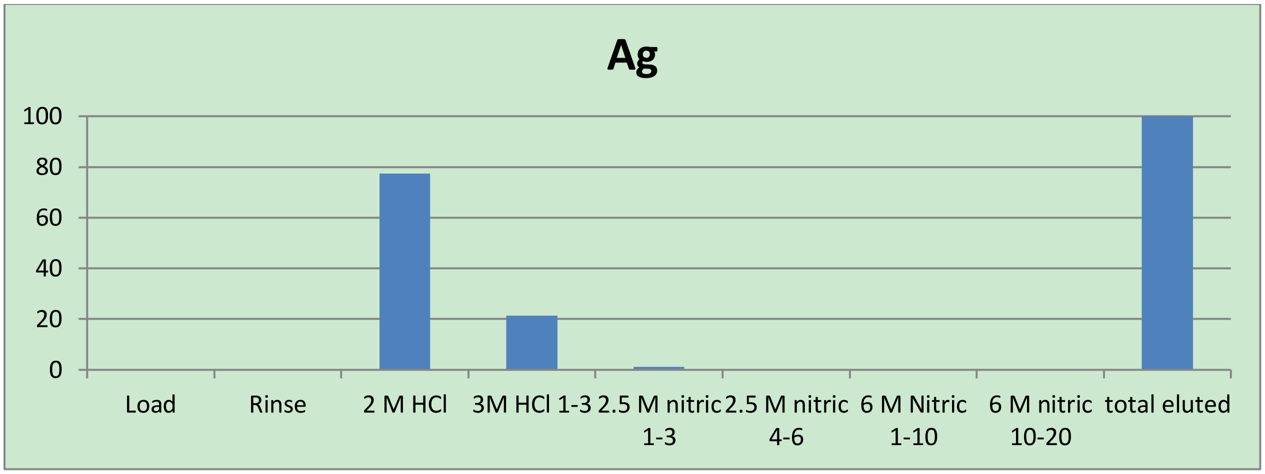

Figure s102. Elution profile of Ag.

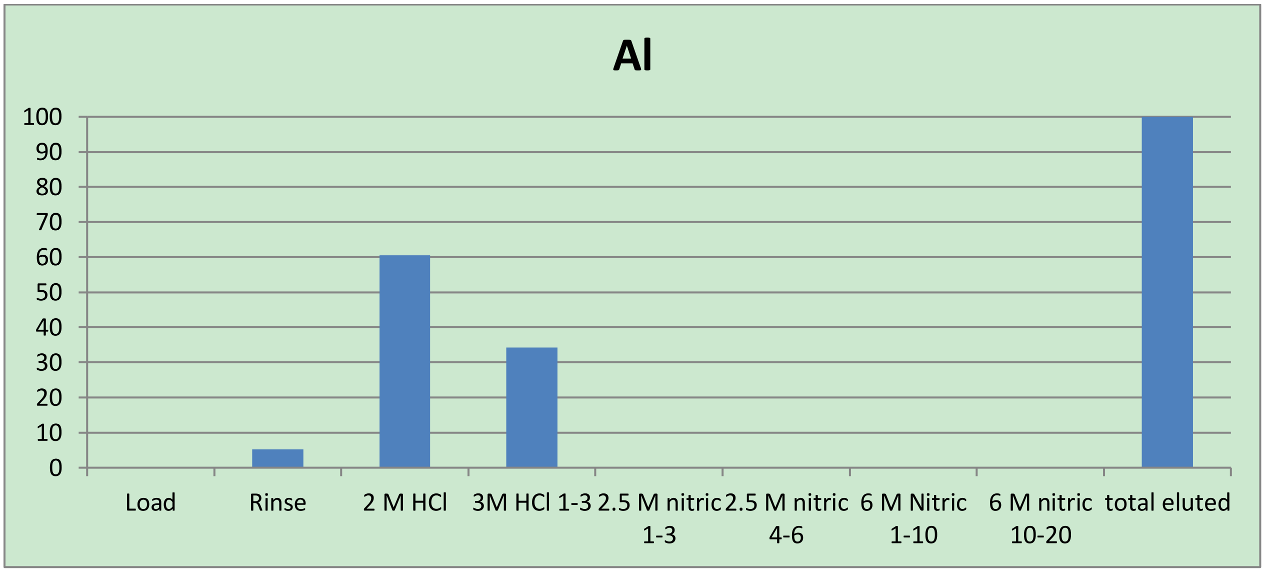

Figure s103. Elution profile of Al.

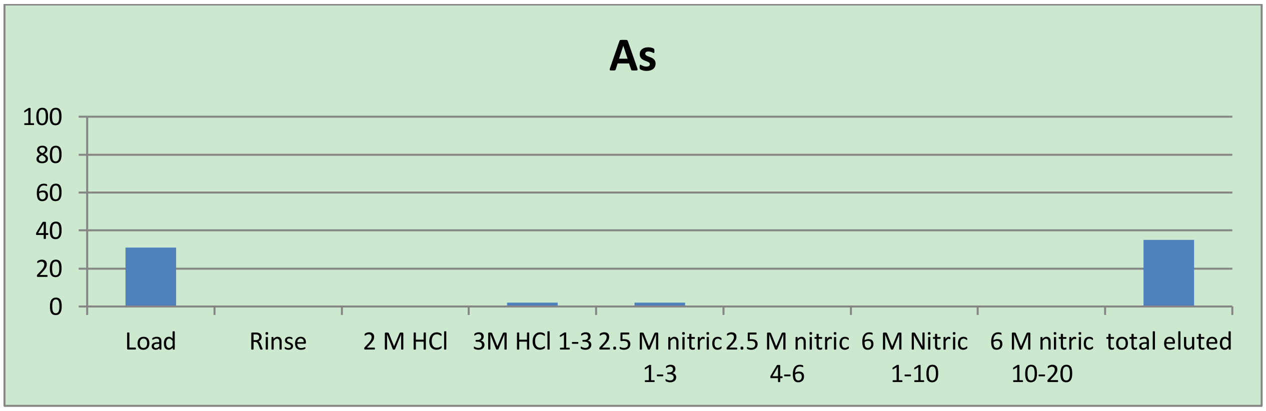

Figure s104. Elution profile of As.

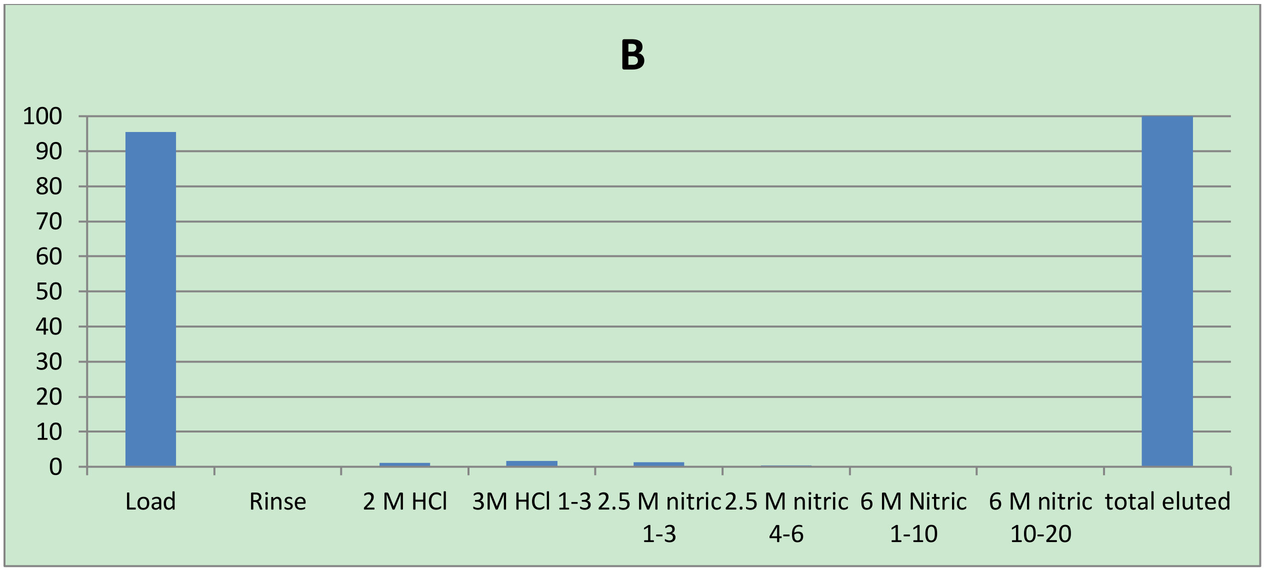

Figure s105. Elution profile of B.

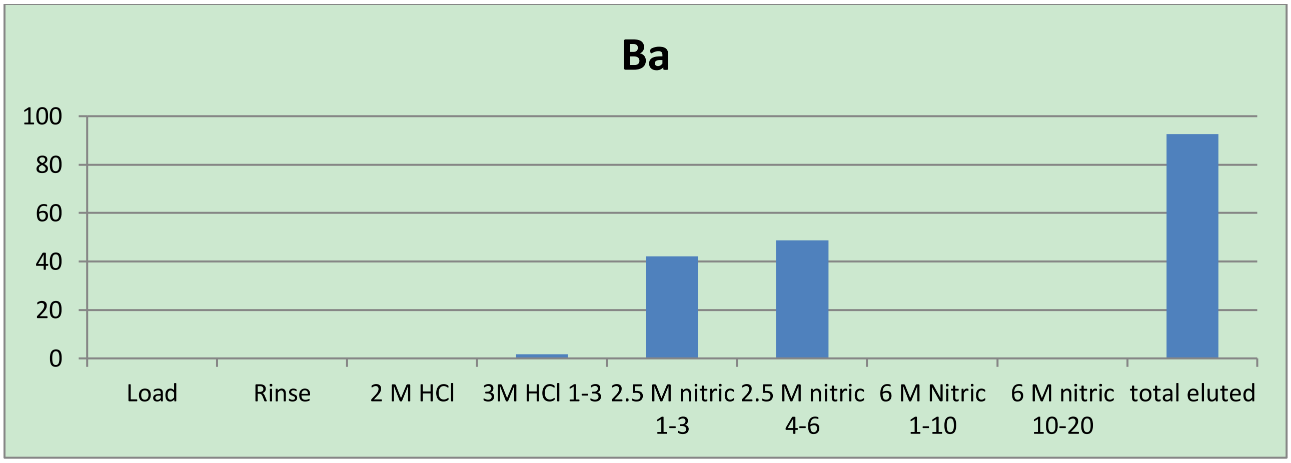

Figure 106. Elution profile of Ba.

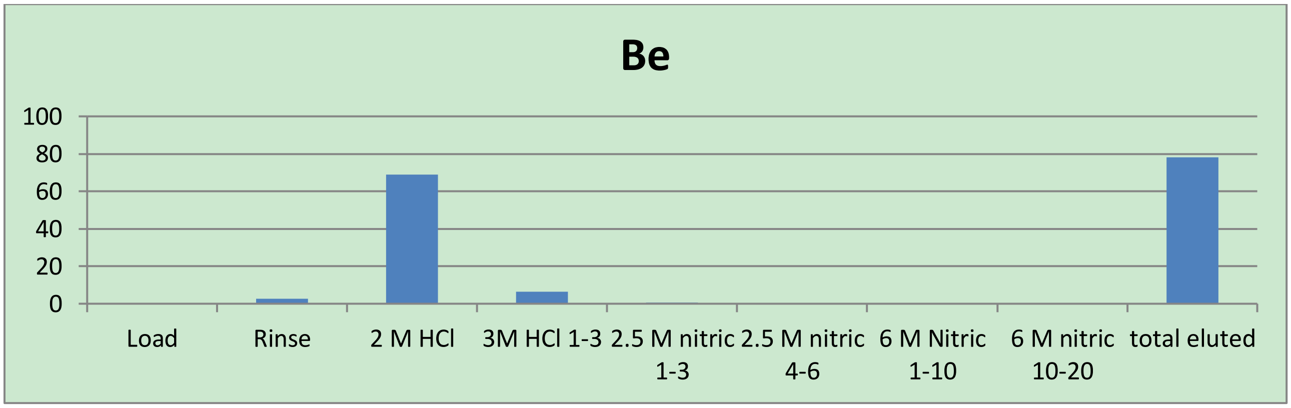

Figure s107. Elution profile of Be.

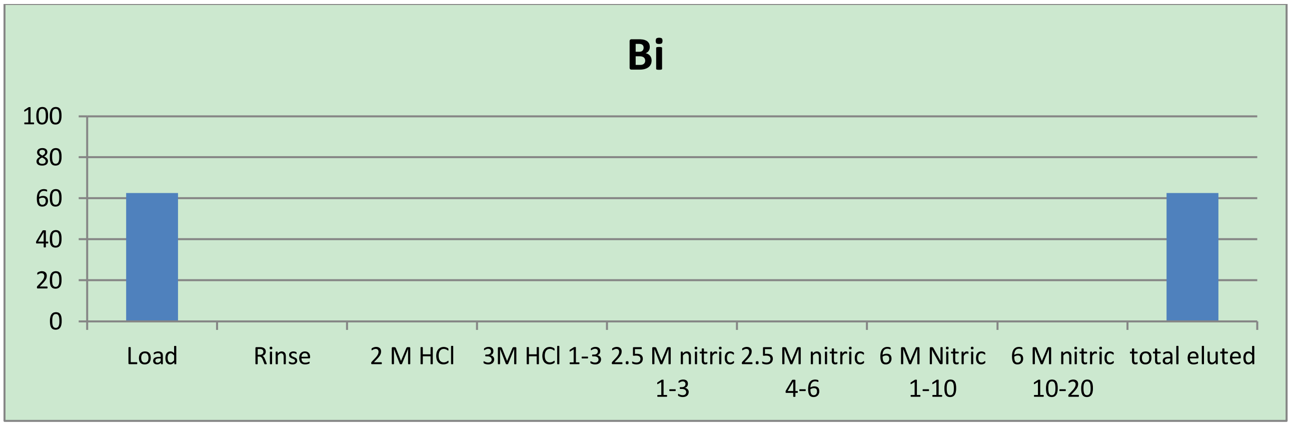

Figure s108. Elution profile of Bi.

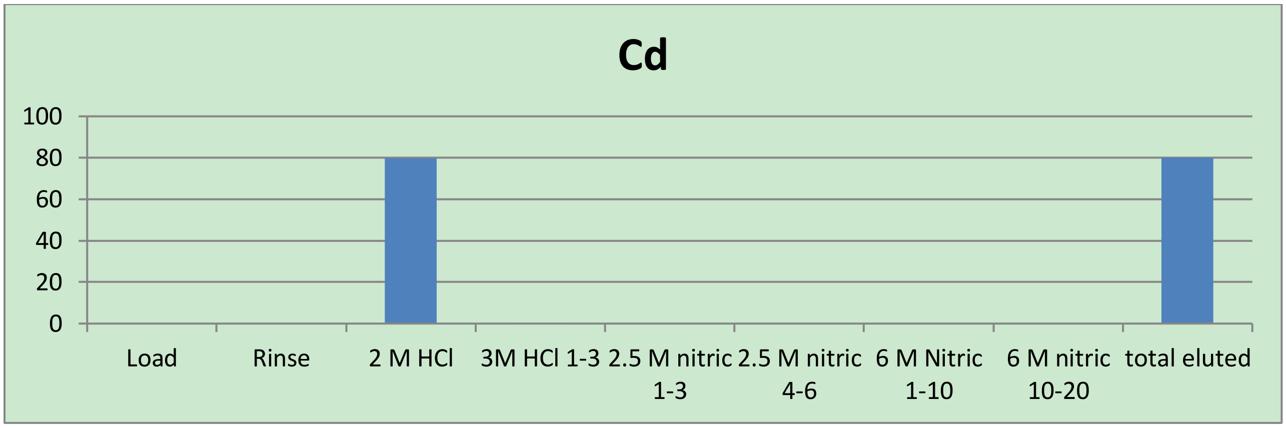

Figure s109. Elution profile of Cd.

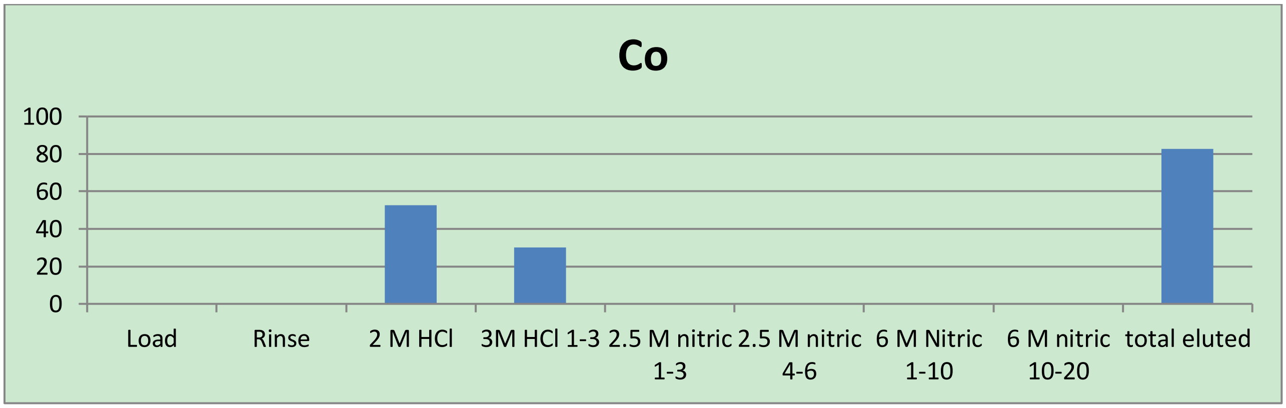

Figure s110. Elution profile of Co.

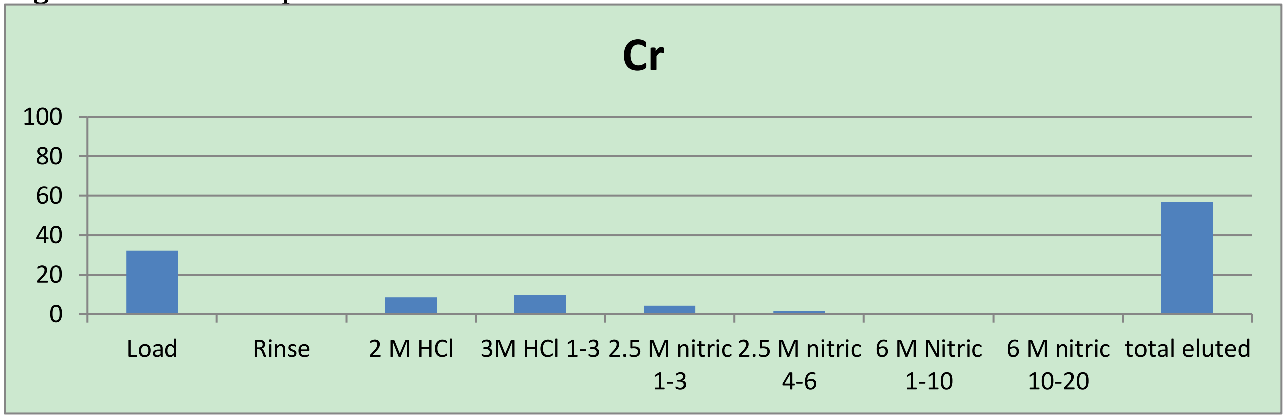

Figure s111. Elution profile of Cr.

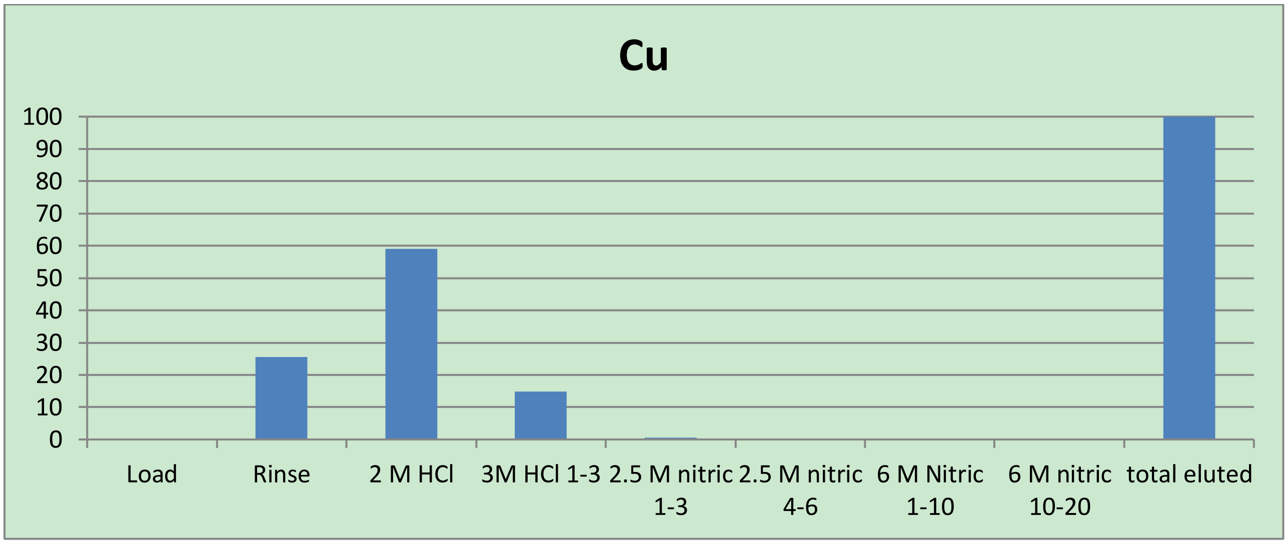

Figure s112. Elution profile of Cu.

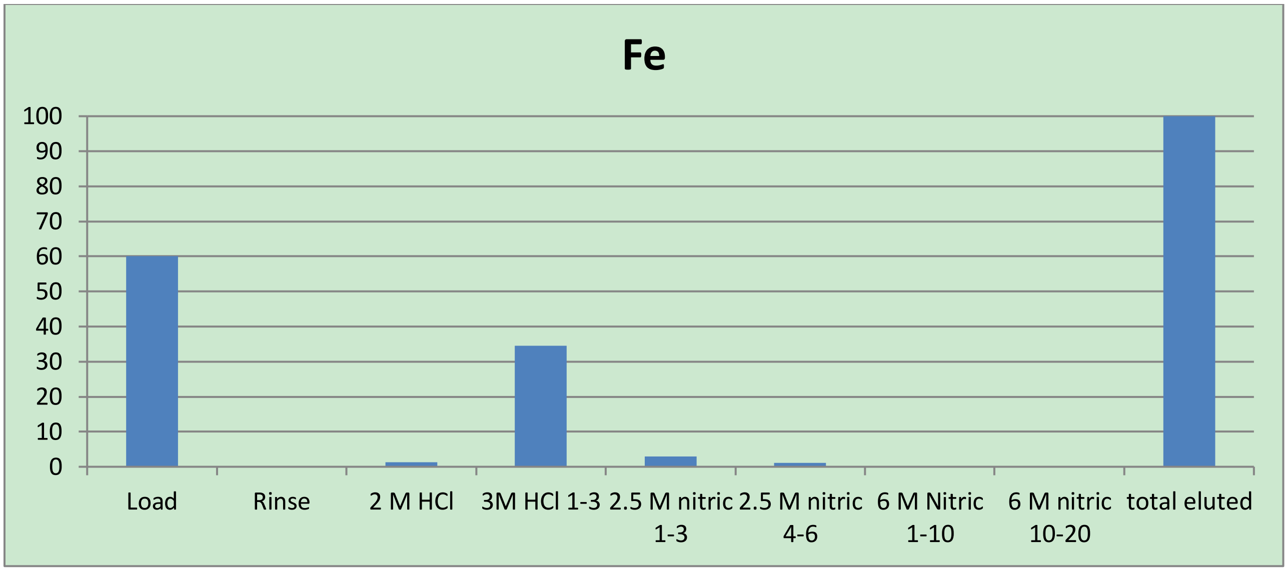

Figure s113. Elution profile of Fe.

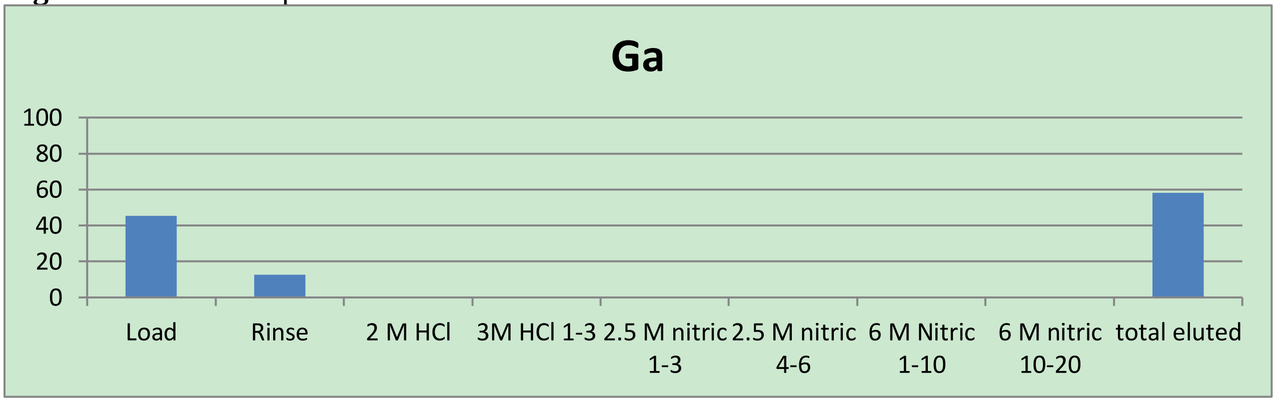

Figure s114. Elution profile of Ga.

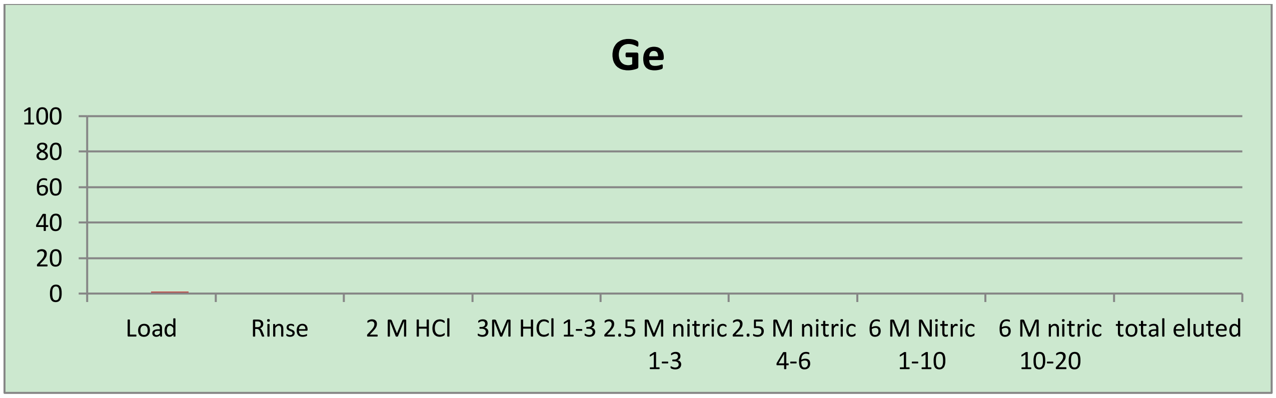

Figure s115. Elution profile of Ge.

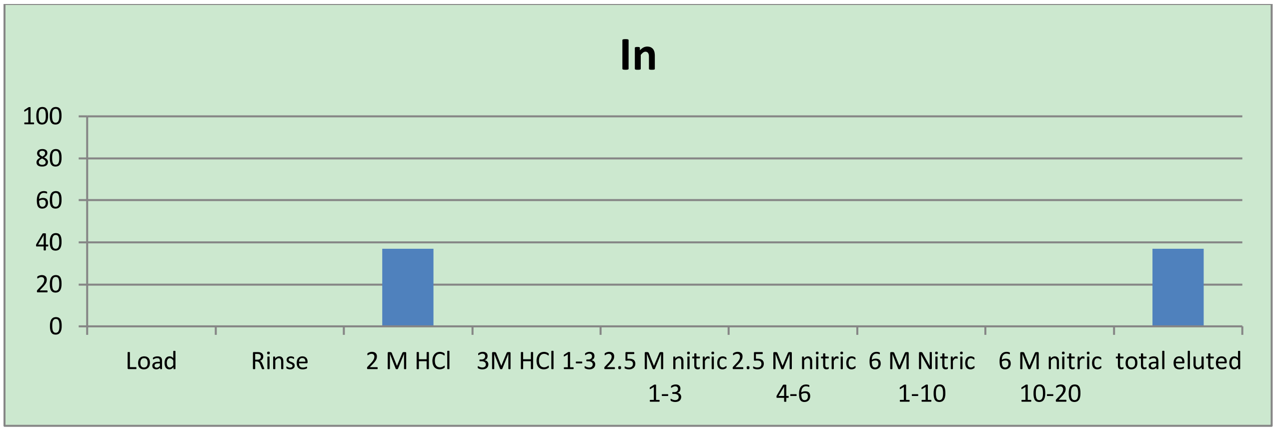

Figure s116. Elution profile of In.

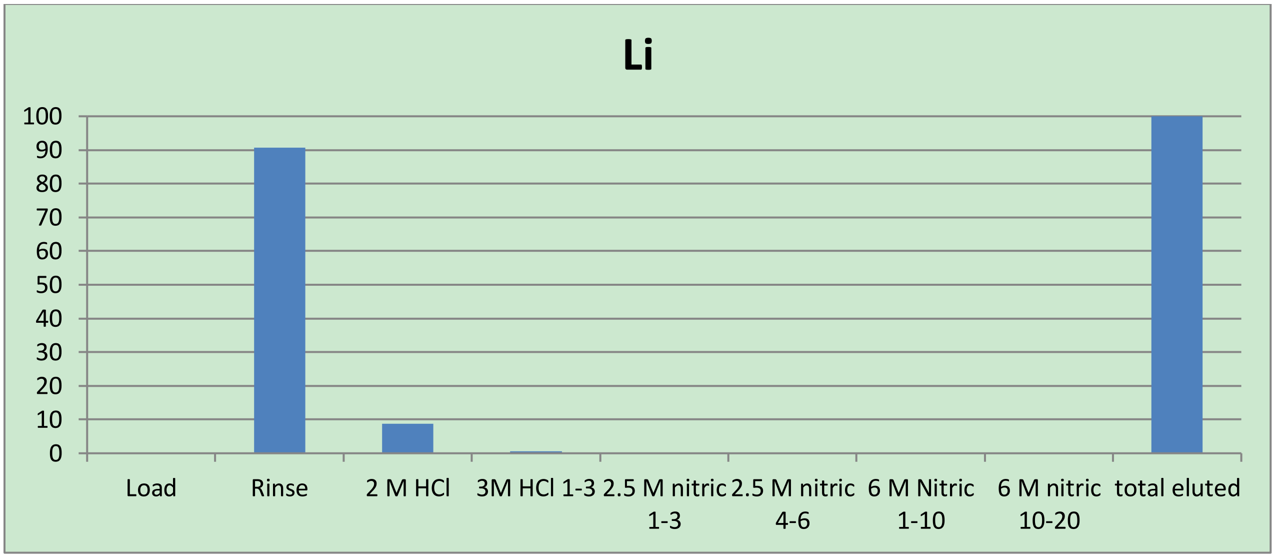

Figure s117. Elution profile of Li.

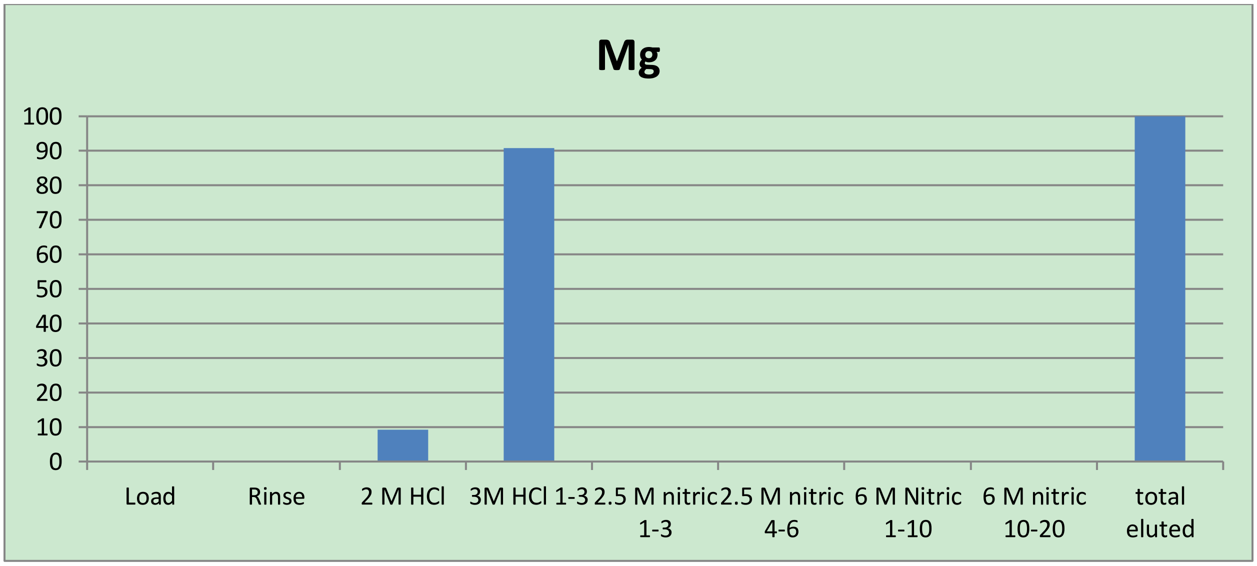

Figure s118. Elution profile of Mg.

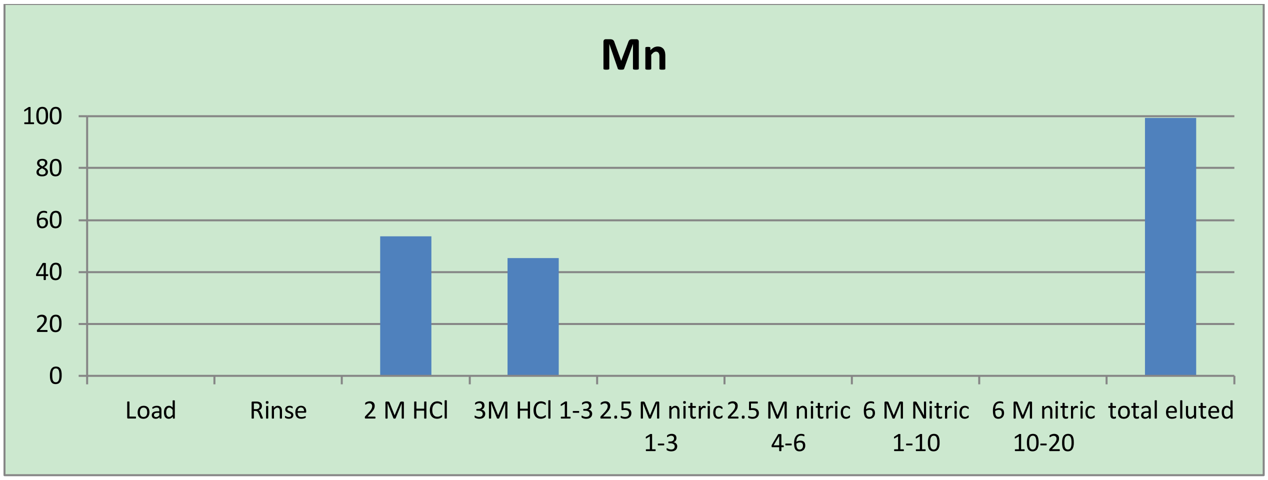

Figure s119. Elution profile of Mn.

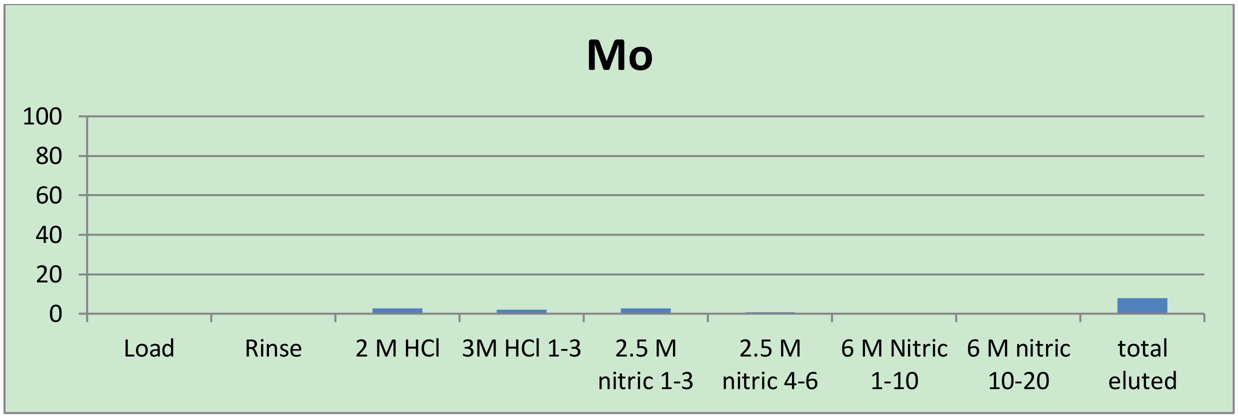

Figure s120. Elution profile of Mo.

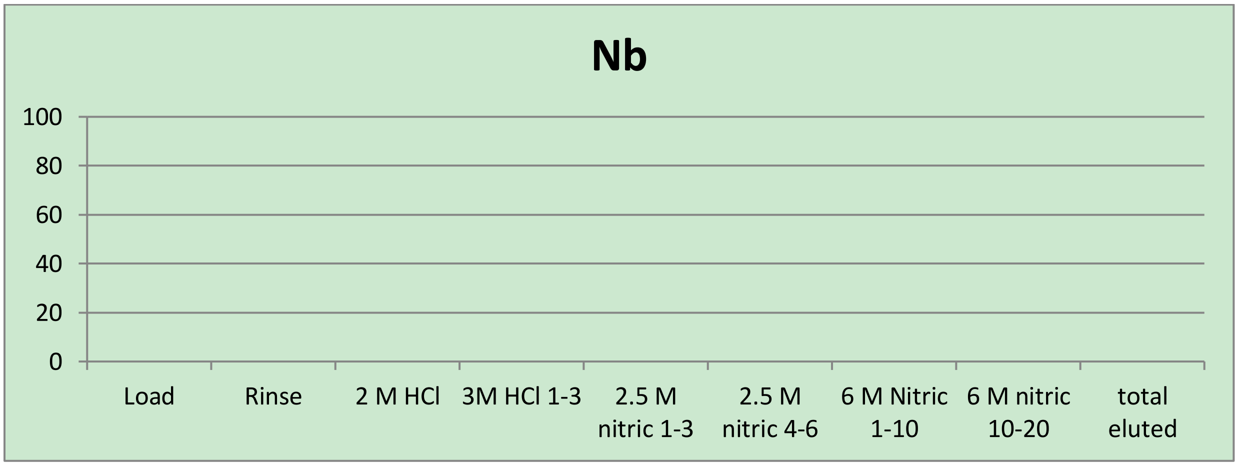

.**Figure s121.** Elution profile of Nb.

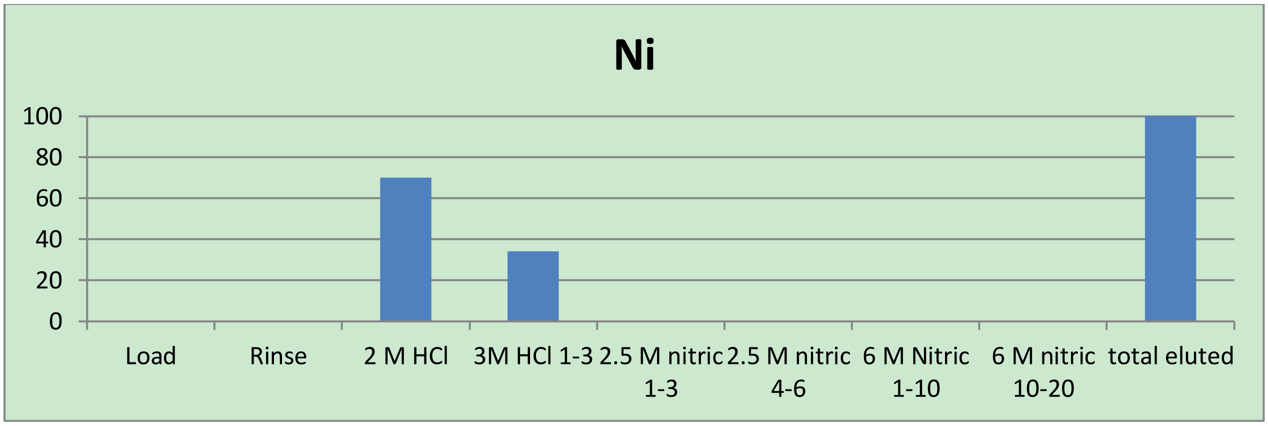

Figure s122. Elution profile of Ni.

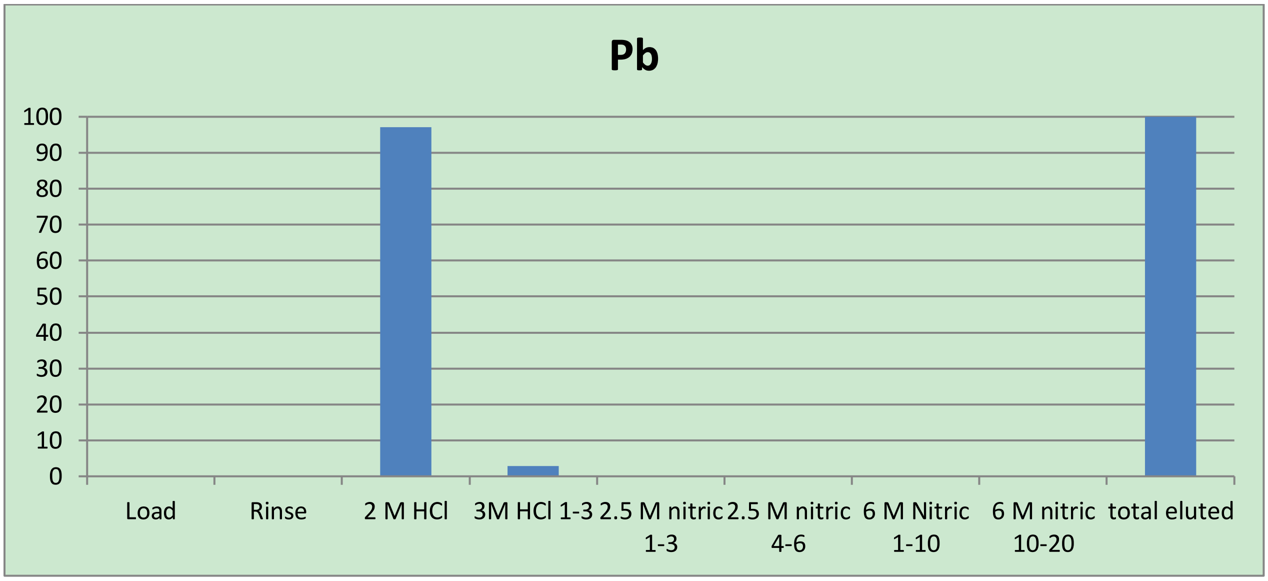

Figure s123. Elution profile of Pb.

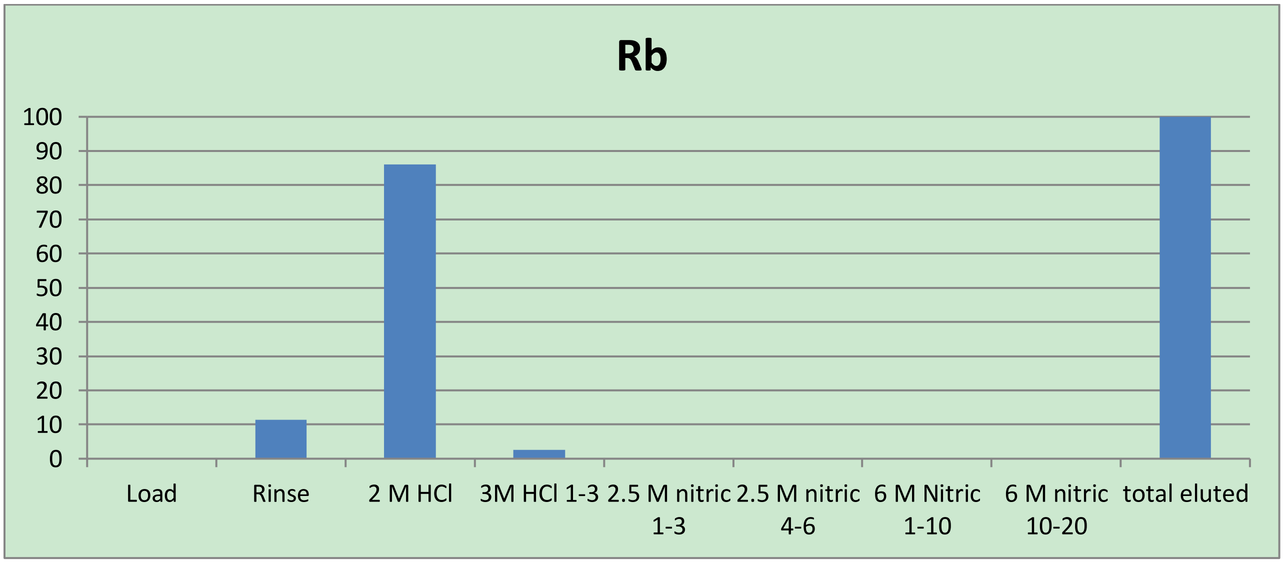

Figure s124. Elution profile of Rb.

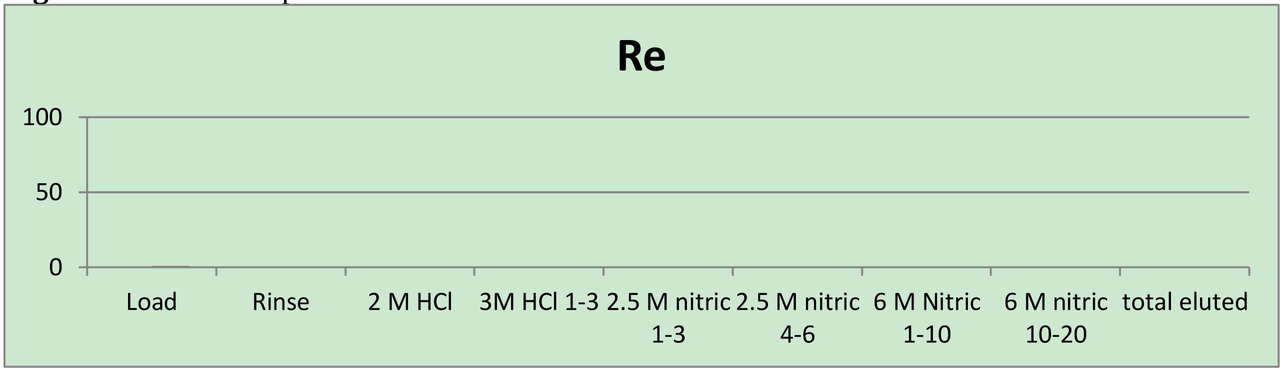

Figure s125. Elution profile of Re.

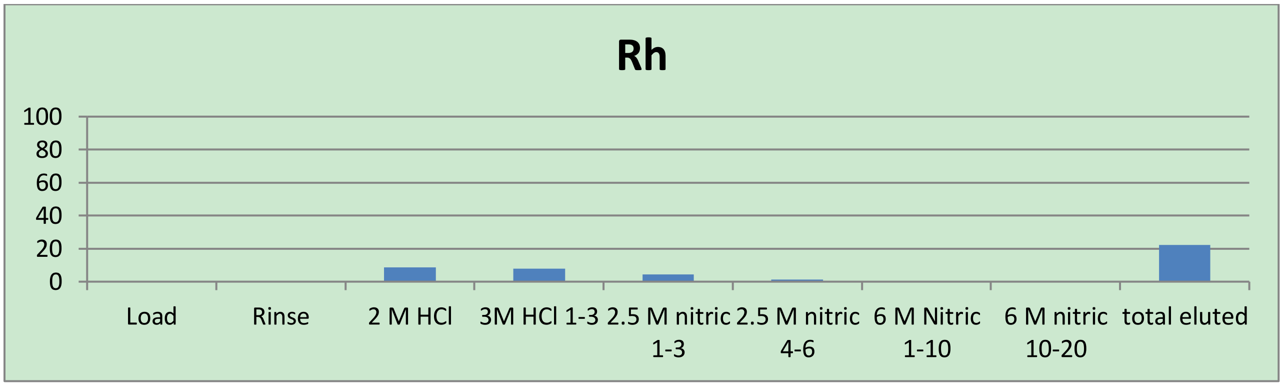

Figure s126. Elution profile of Rh.

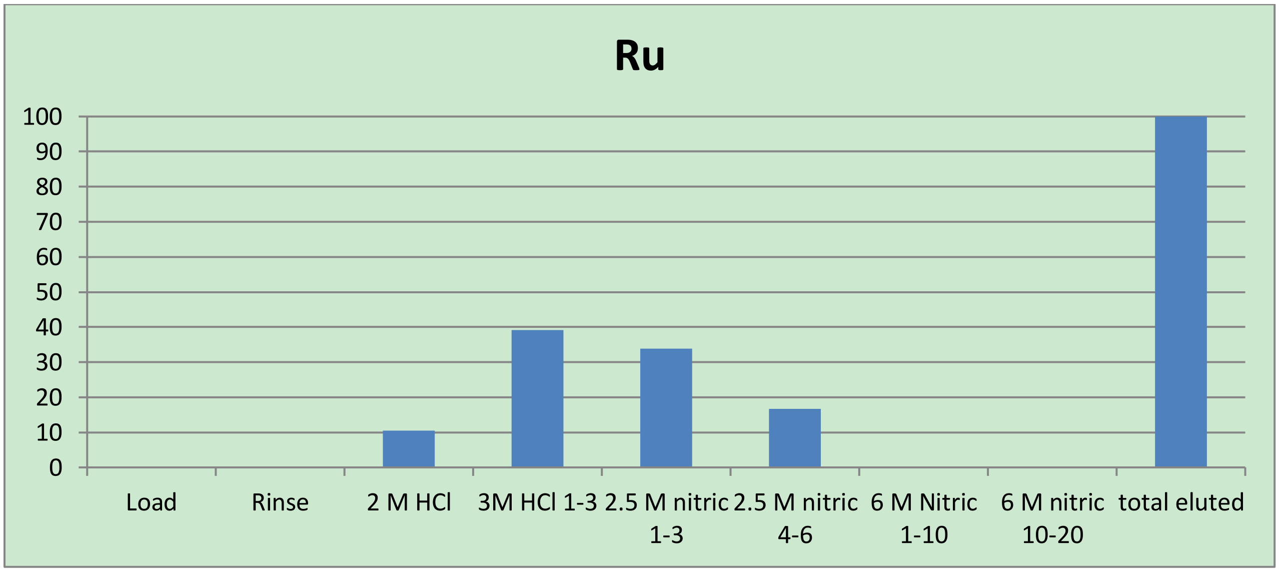

Figure s127. Elution profile of Ru.

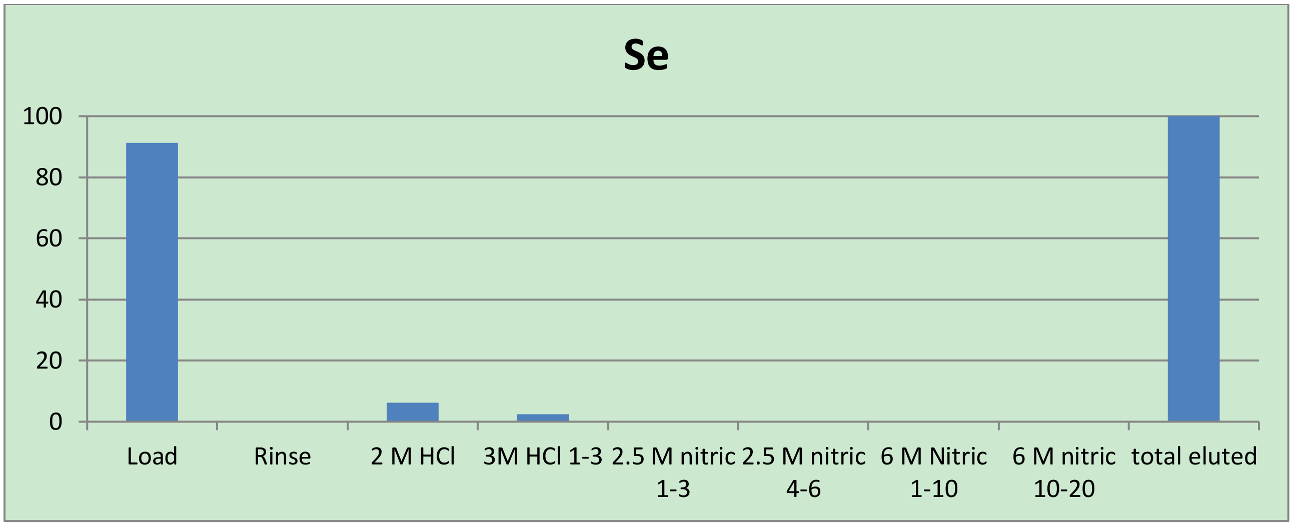

Figure s128. Elution profile of Se.

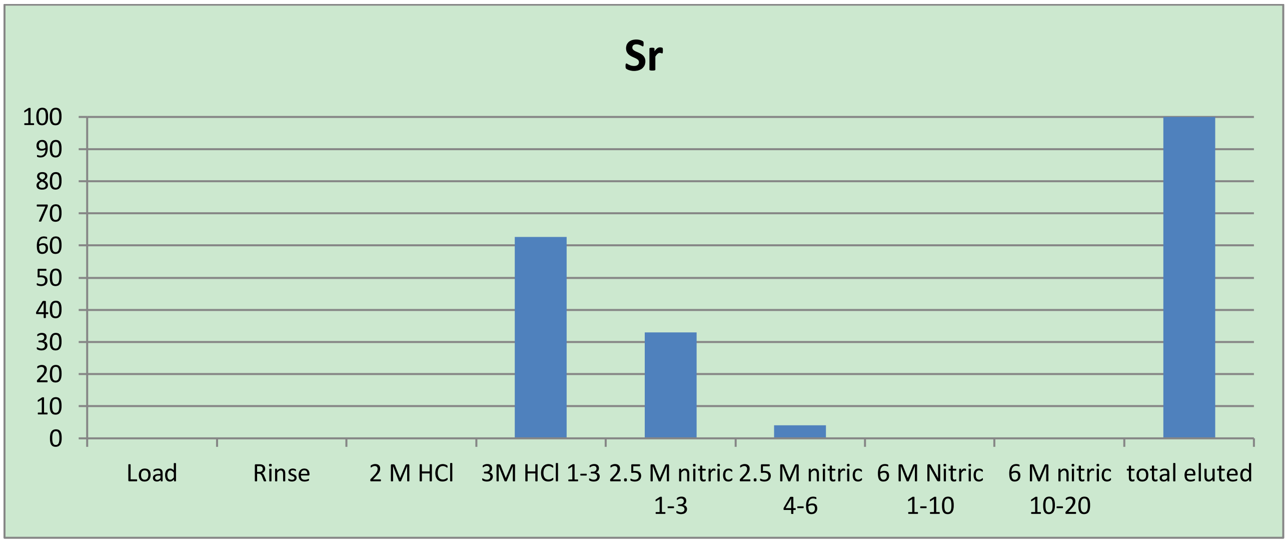

Figure s129. Elution profile of Sr.

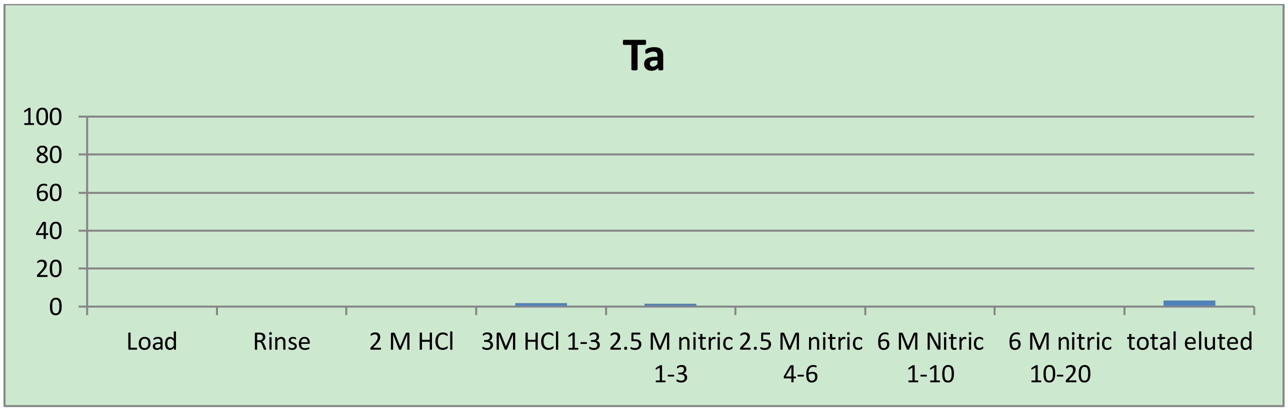

Figure s130. Elution profile of Ta.

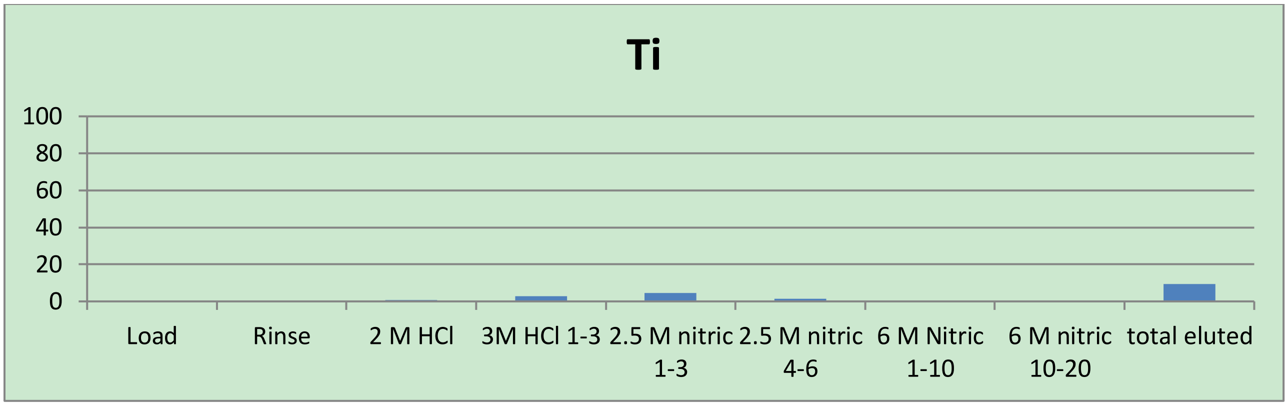

Figure s131. Elution profile of Ti.

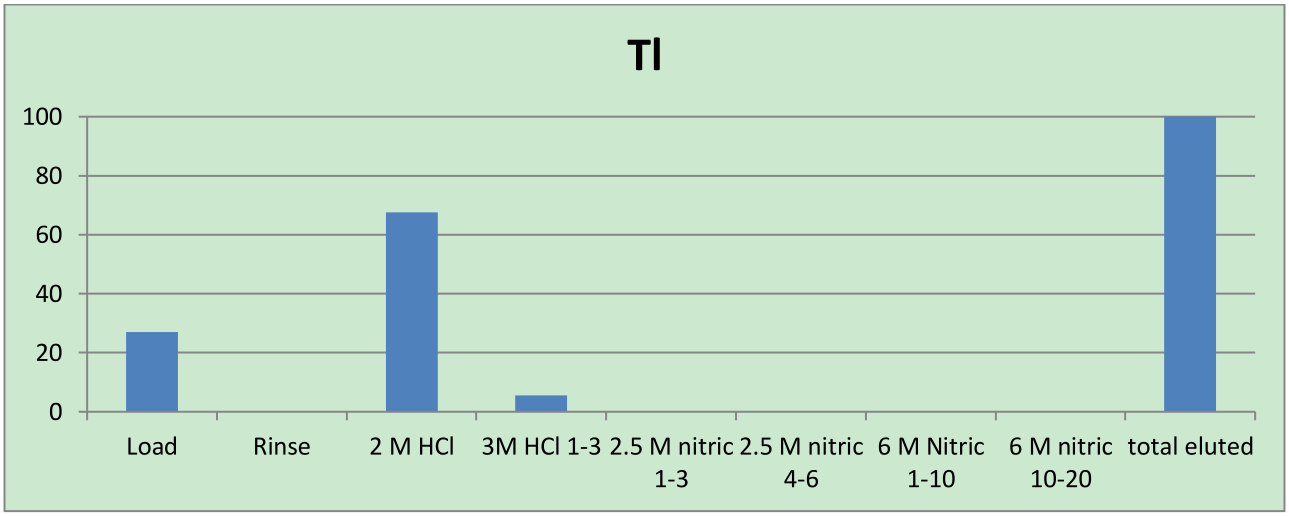

Figure s132. Elution profile of Tl.

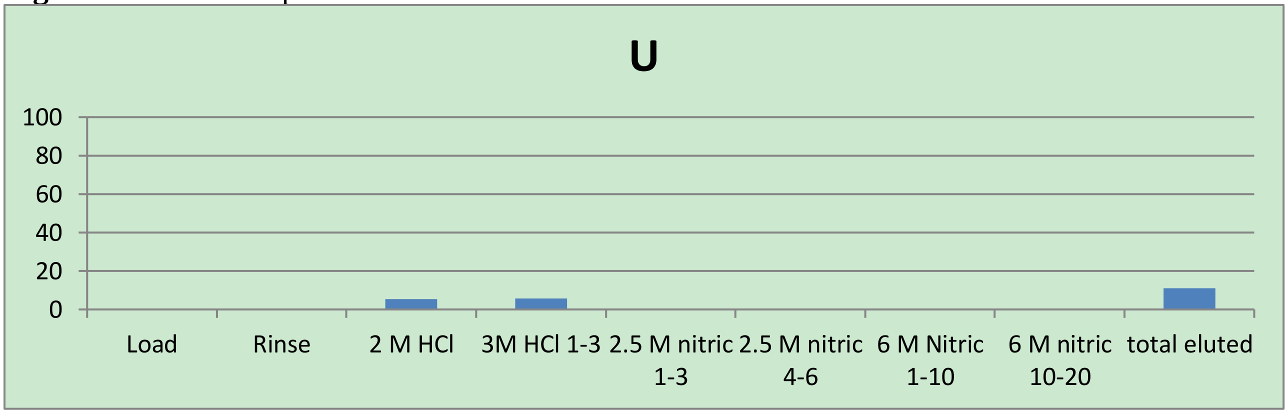

Figure s133. Elution profile of U.

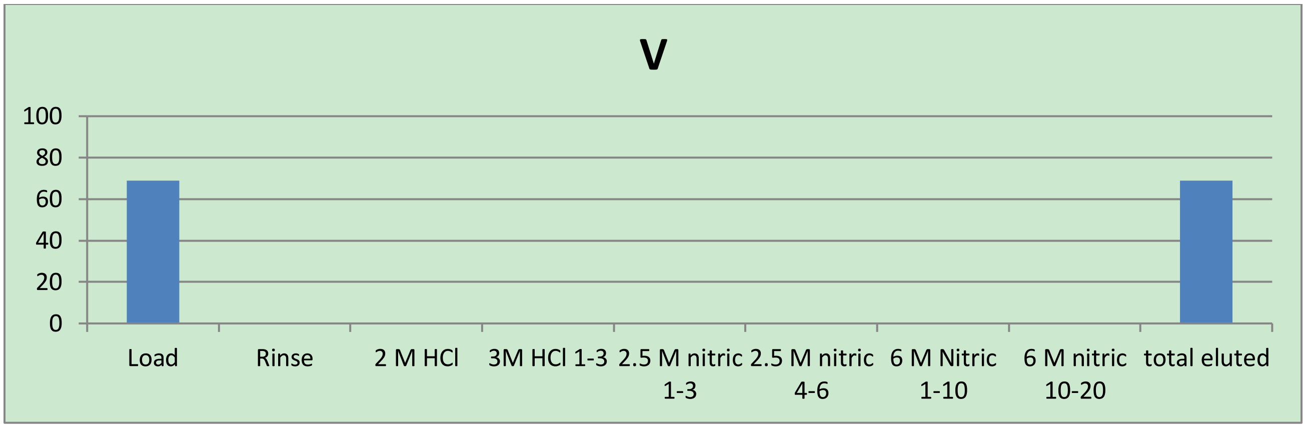

Figure s134. Elution profile of V.

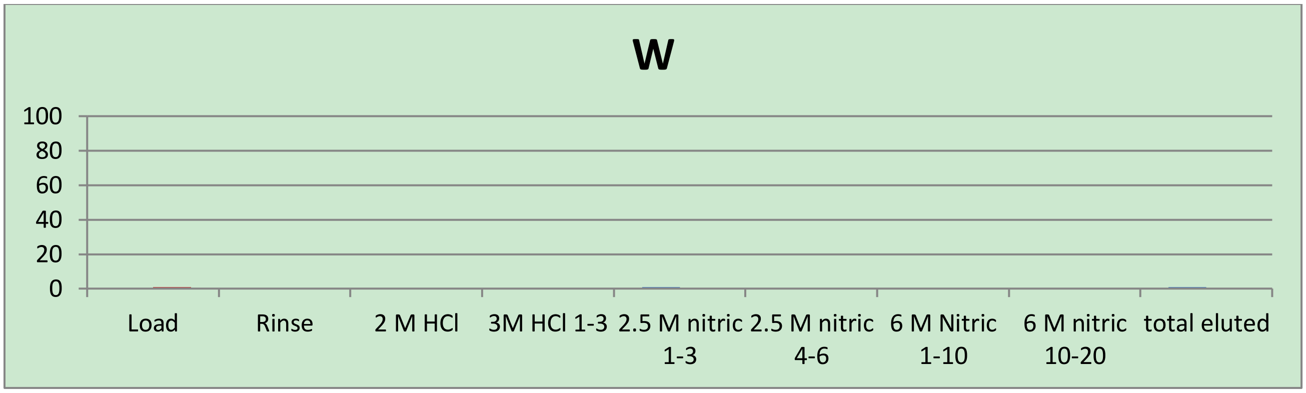

Figure s135. Elution profile of W.

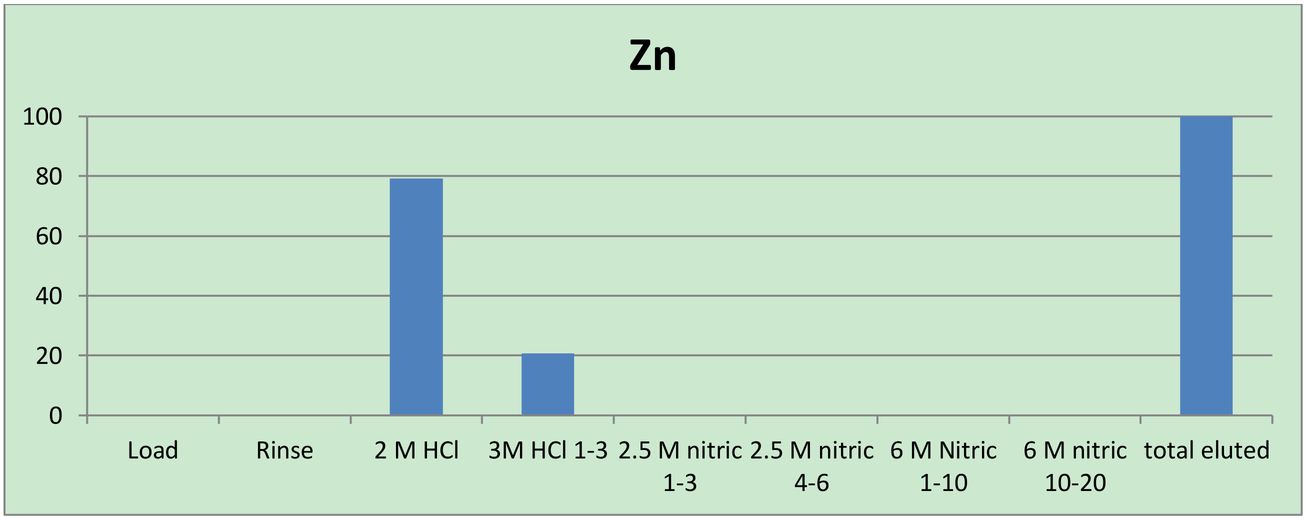

Figure s136. Elution profile of Zn.

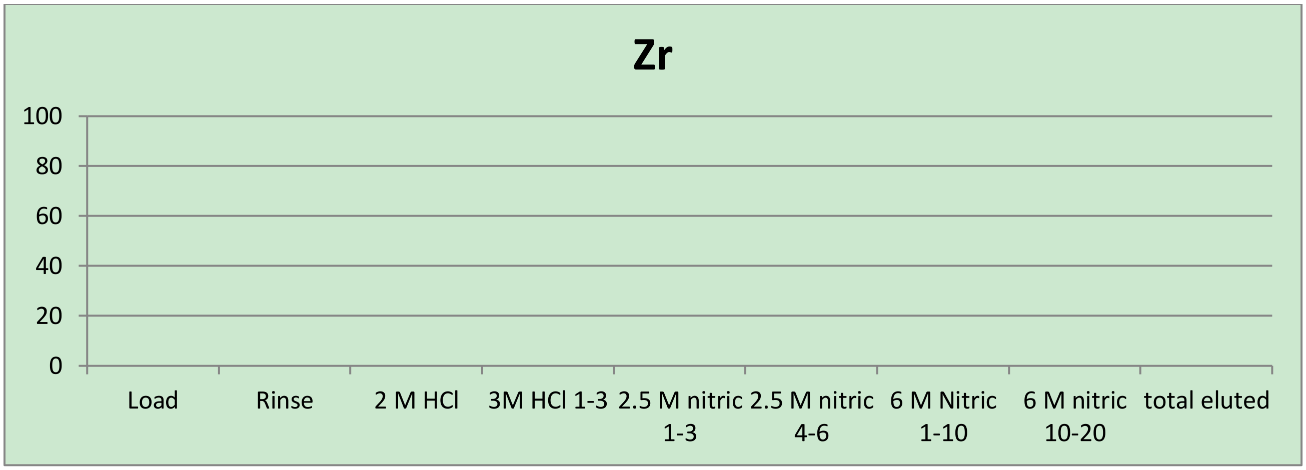

Figure s137. Elution profile of Zr.

Lanthanides and Th

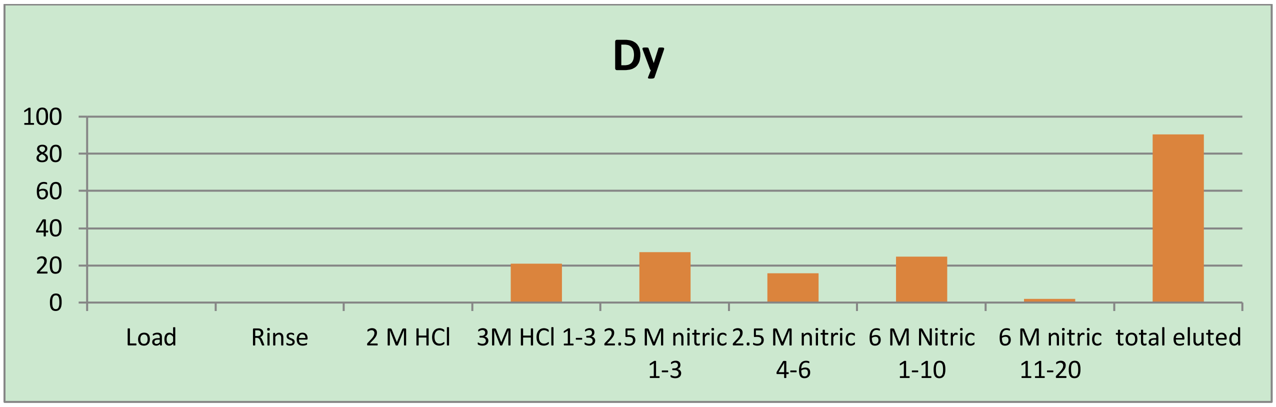

Figure s138. Elution profile of Dy.

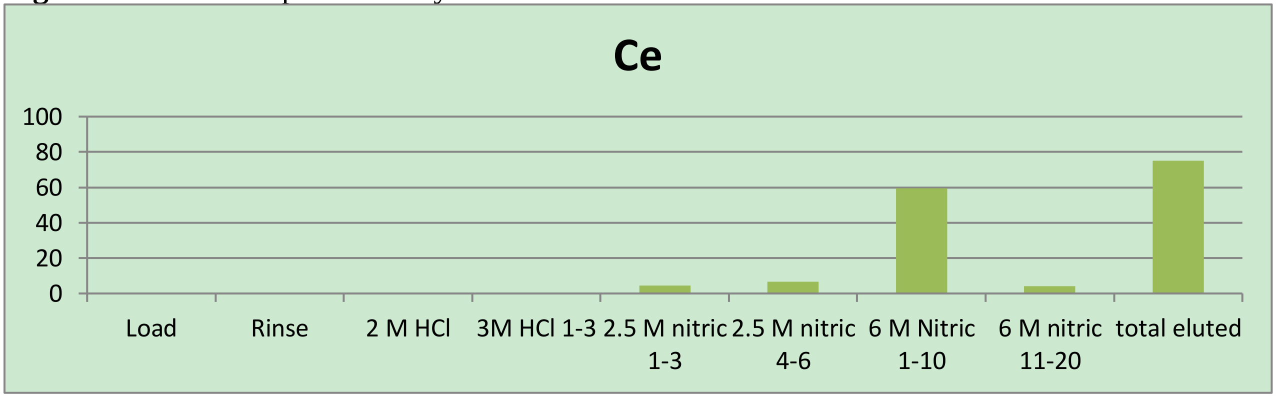

Figure s139. Elution profile of Ce.

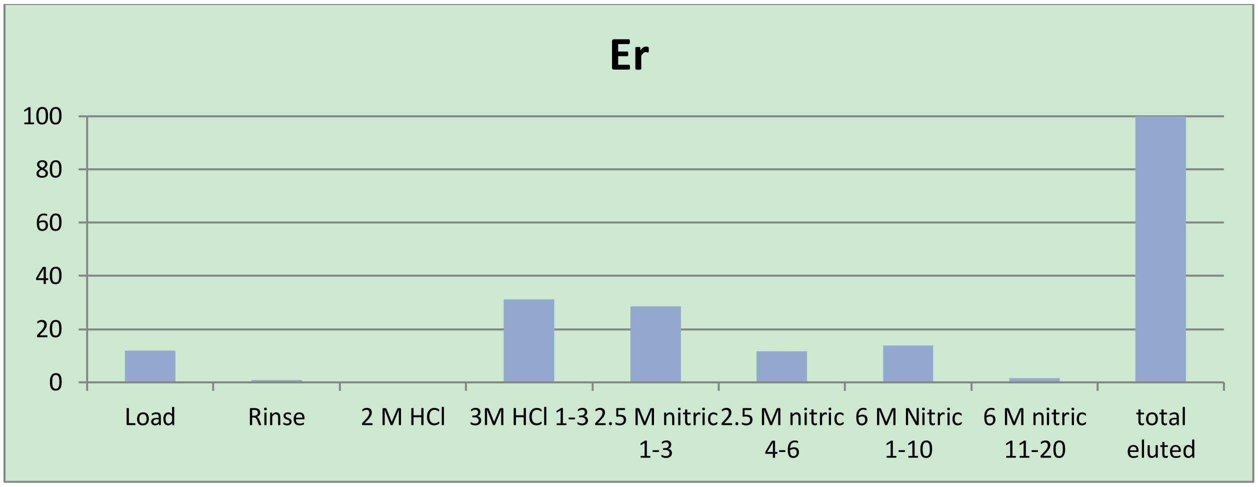

Figure s140. Elution profile of Er.

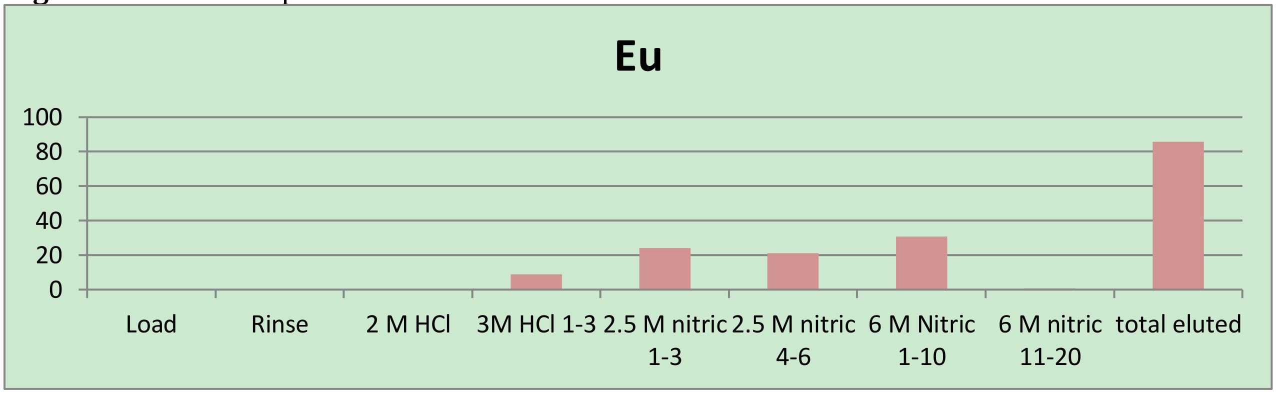

Figure s141. Elution profile of Eu.

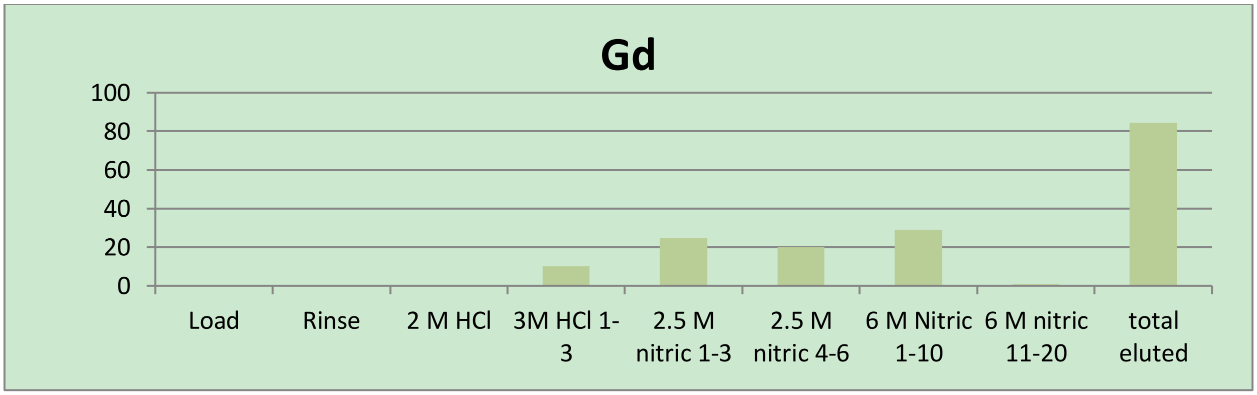

Figure s142. Elution profile of Gd.

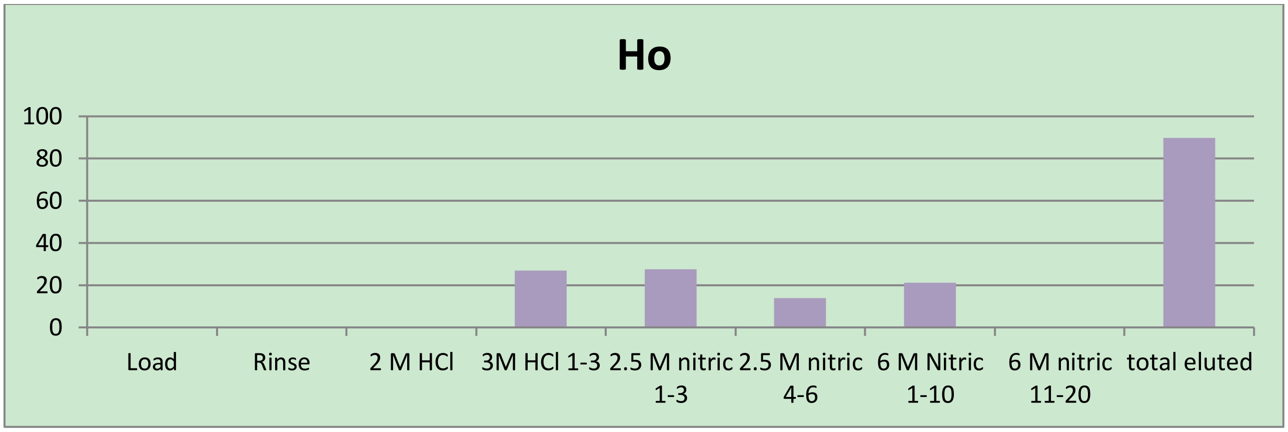

Figure s143. Elution profile of Ho.

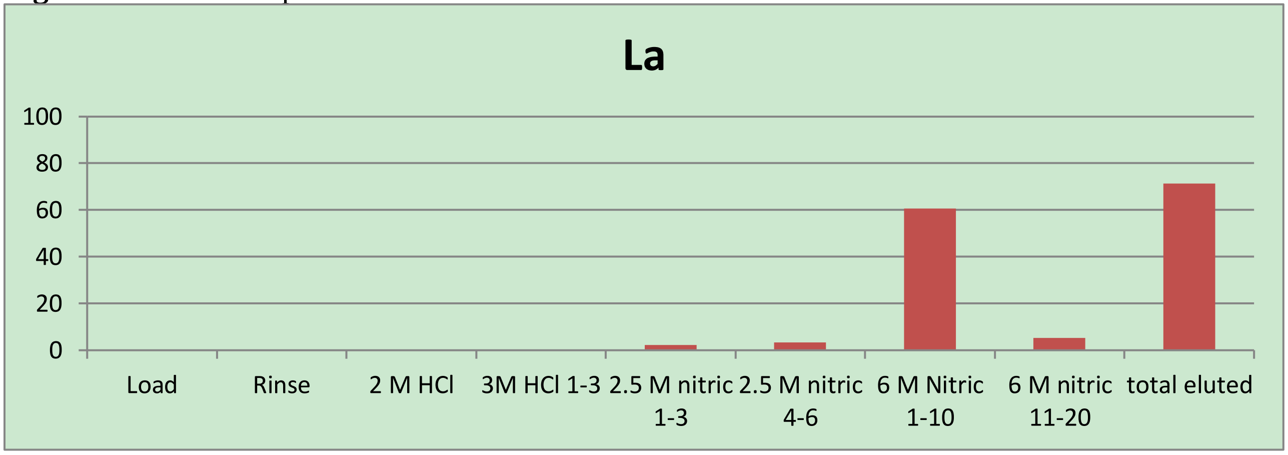

Figure s144. Elution profile of La.

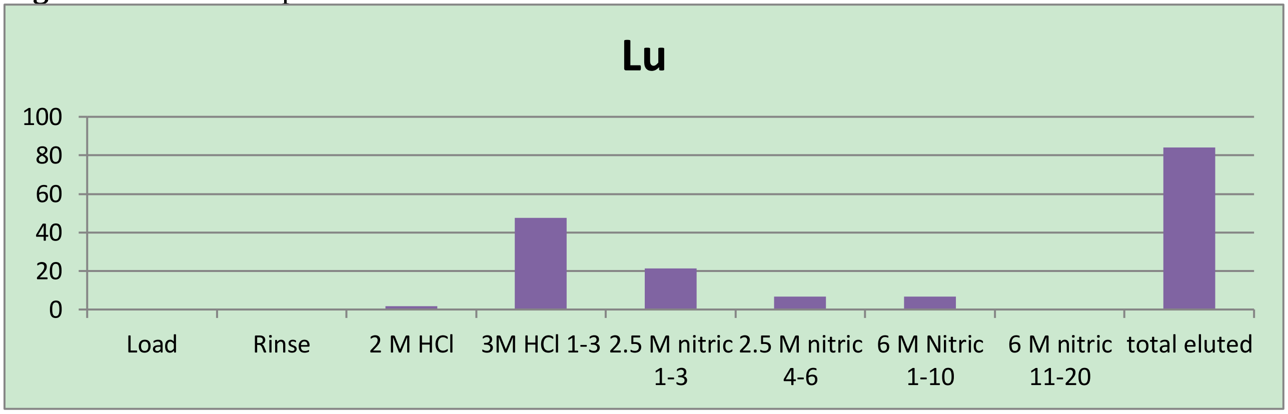

Figure s145. Elution profile of Lu.

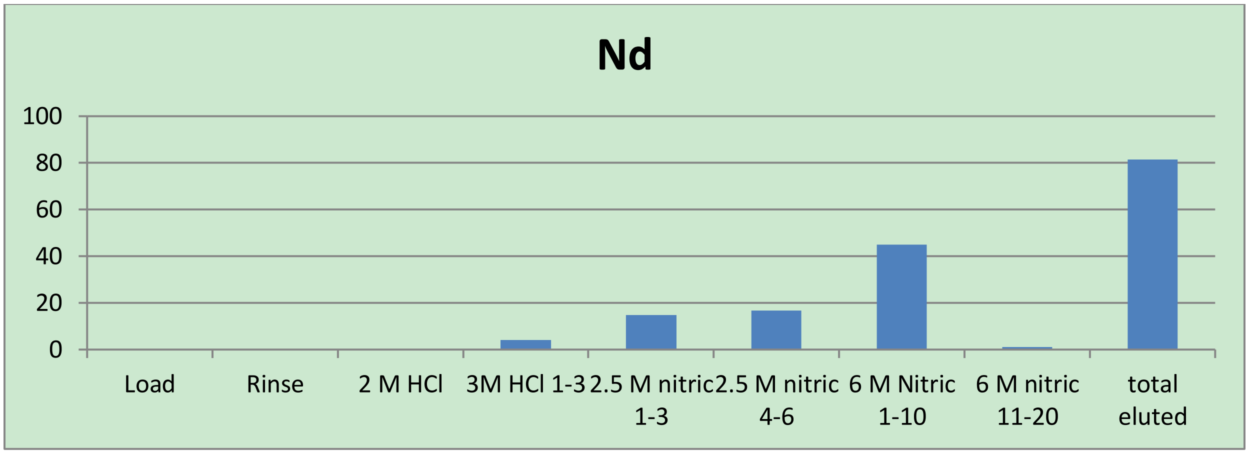

Figure s146. Elution profile of Nd.

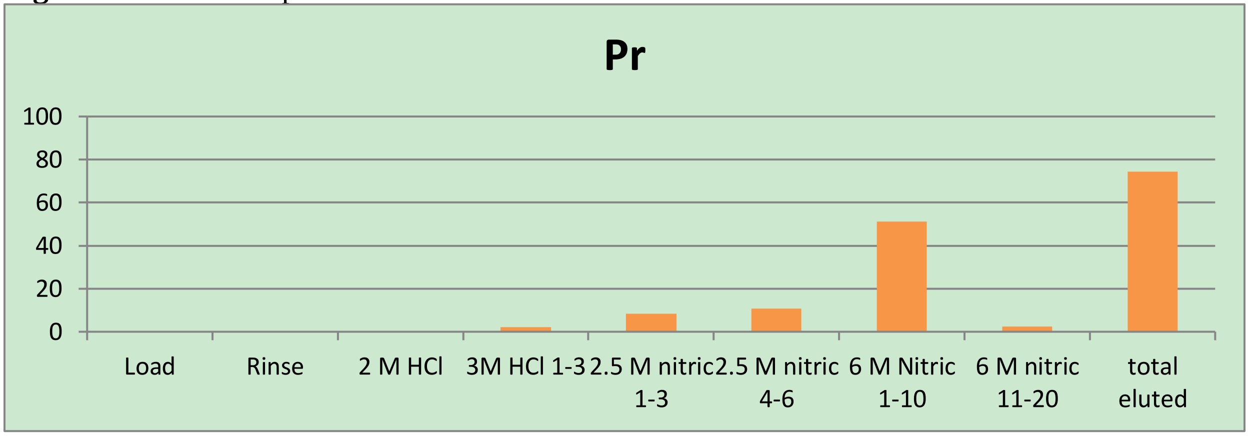

Figure s147. Elution profile of Pr.

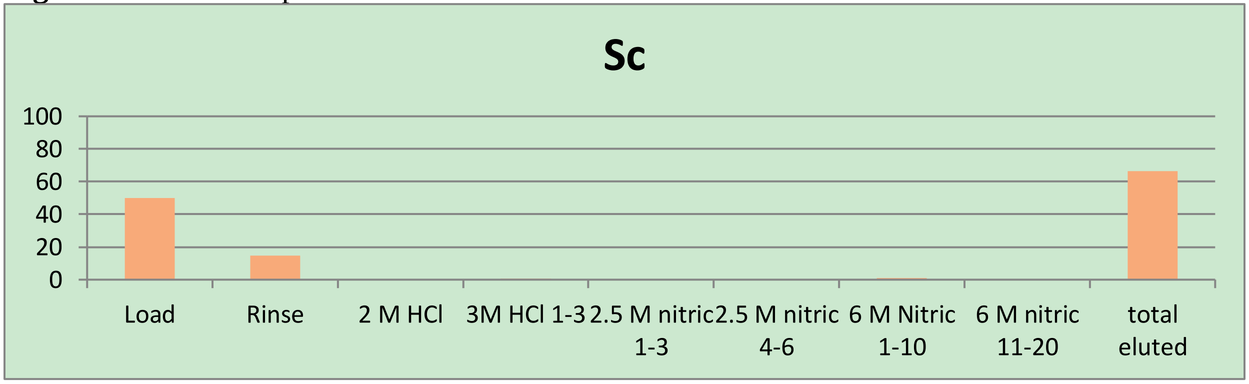

Figure s148. Elution profile of Sc.

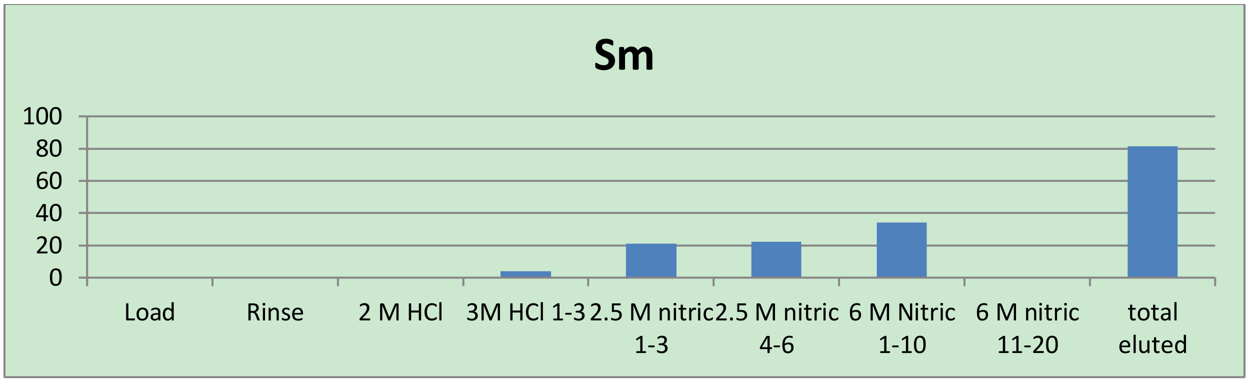

Figure s149. Elution profile of Sm.

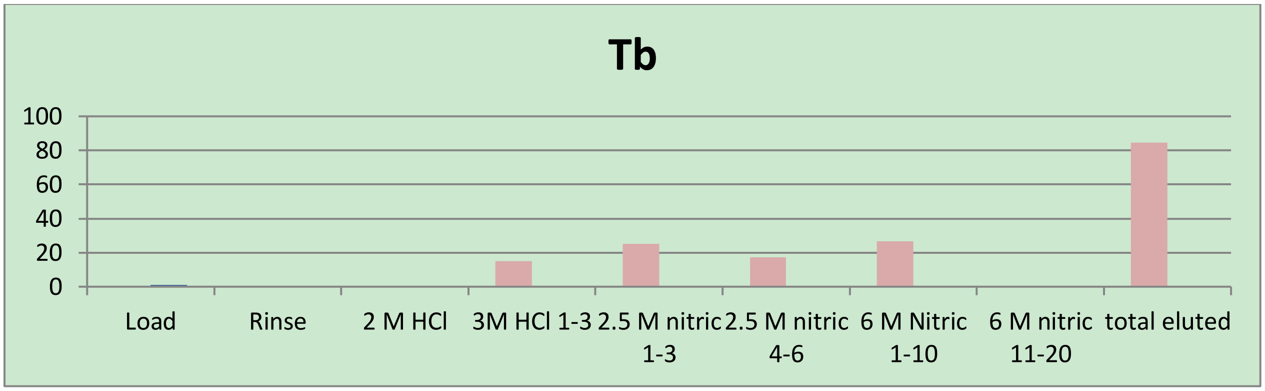

Figure s150. Elution profile of Tb.

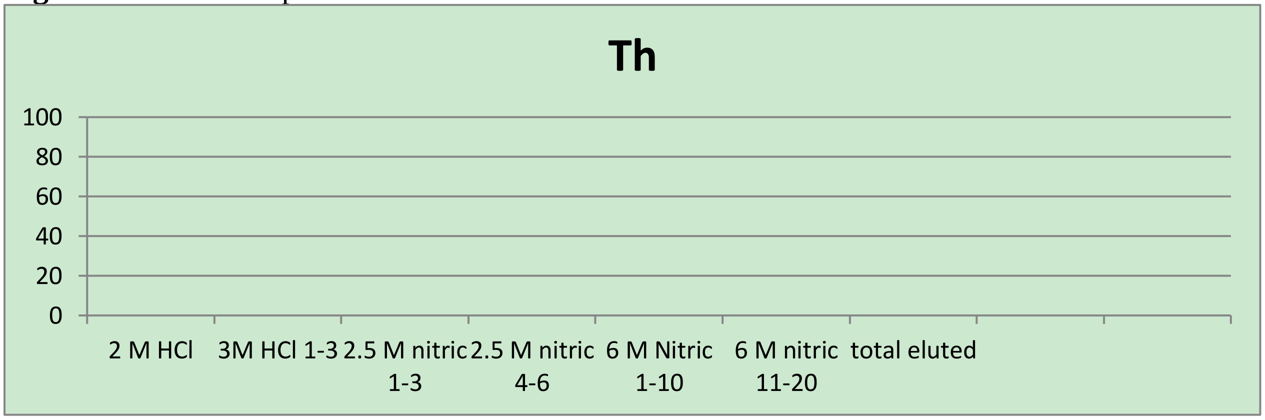

Figure s151. Elution profile of Th.

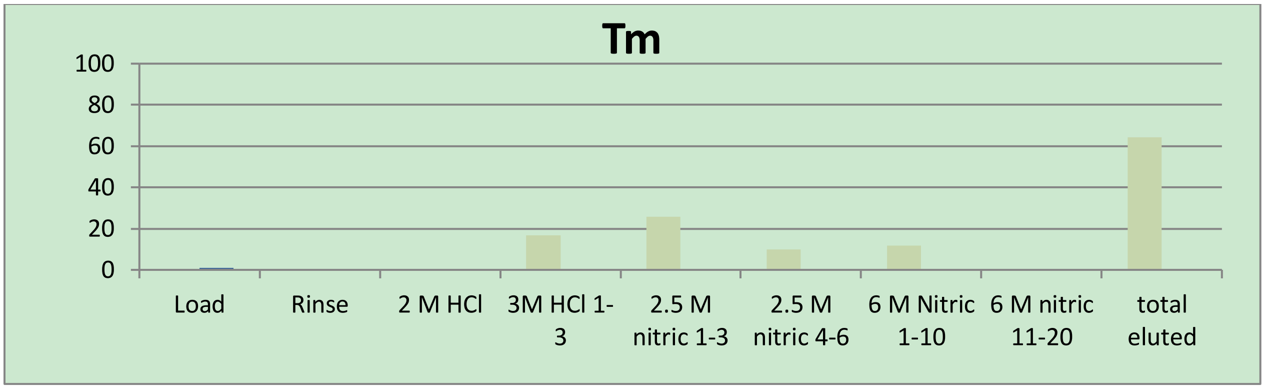

Figure s152. Elution profile of Tm.

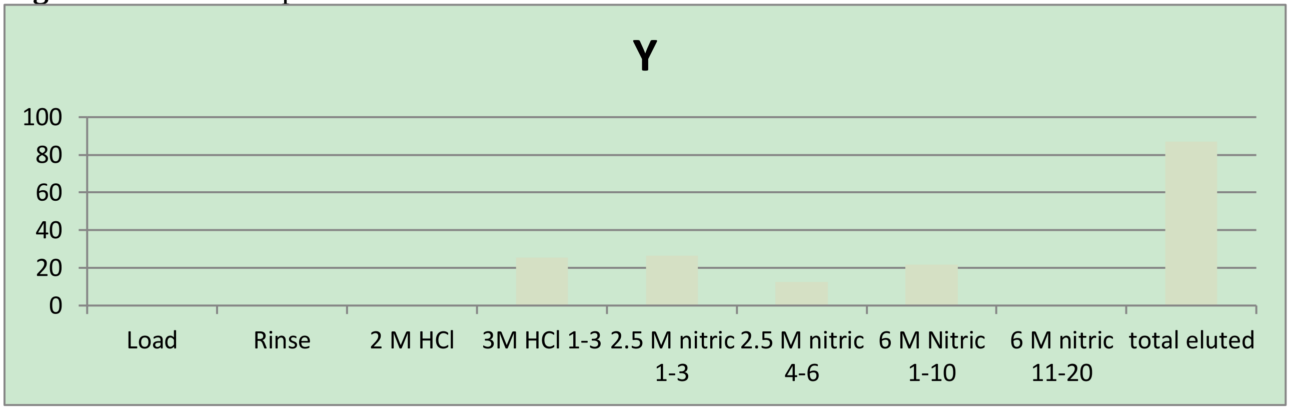

Figure s153. Elution profile of Y.

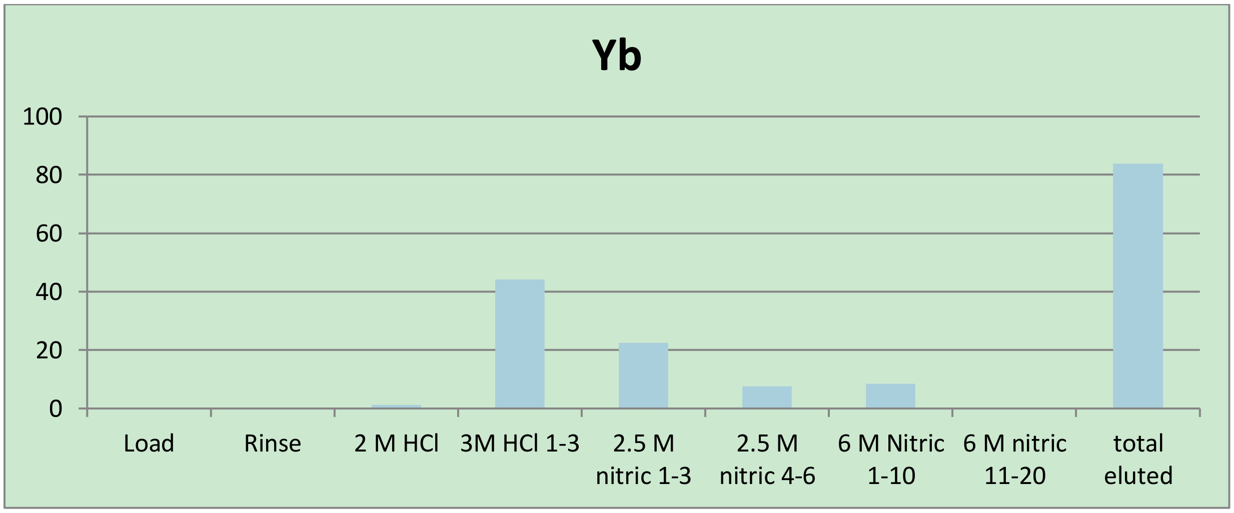

Figure s154. Elution profile of Yb.

Examine rinse sequence with mixed metals and thorium

Experiment: Experimental method summarized in manuscript.

Table S1. Percent of various elements in the various steps of the separation using the rinse sequence: 3 ml of 1 M citric acid pH 2, 3 ml of water, 2X 3 ml of 2.5 M HCl, 3 ml of water, 2x 3 ml of 2.5 M nitric acid, 2x10 ml of 6 M nitric acid. Bolded values are the highest value for the elution of the element, and empty boxes represent samples that were below 1% or not detected. NM not measured.

	Load	Citric	water	2.5 M HCl	2.5 M HCl	water	2.5 M HNO_3	2.5 M HNO_3	6 M HNO_3	6 M HNO_3
Al	NM	**15.8**		3.6	2.6			1.8	7.2	11.1
Ba	NM			3.7	13.4	6.0	**62.0**	13.8		
Be	NM	**83.3**	1.0	23.1	1.1				6.6	3.0
Cd	NM	10.2		**62.3**						
Co	NM			**60.0**	4.8					
Cr	NM	6.2		22.8	**26.9**	2.5	1.8			
Fe	NM	**61.4**	1.1	10.4	2.0			1.7	5.8	1.9
Mn	NM		3.4		**74.0**	6.9				
Mg	NM	9.1		**81.5**	4.7			1.0	4.2	3.4
Ni	NM			**54.7**	5.6			1.5		
Pb	NM	9.3		**79**				2.1	5.0	2.5
^{223}Ra	6.2	2.1			4.8	1.4	**54.8**	21.5	7.3	1.1
Zn	NM	16.6		53.5			1.8		2.1	

Lanthanides

	Load	Citric	water	2.5 M HCl	2.5 M HCl	water	2.5 M HNO_3	2.5 M HNO_3	6 M HNO_3	6 M HNO_3
Ce	NM	NM	6.8				3.3	5.7	**43.7**	18.5
Dy	NM	NM		30.3	**59.0**	1.1	1.3	1.8	4.7	1.8
Eu	NM	18.7	5.4	5.0		2.8	4.7	**43.1**	19.9	18.7
Gd	NM	NM					2.4	5.5	**60.6**	23.5
Ho	NM	3.1	5.2	6.3	2.2	6.1	8.5	**47.7**	21.0	3.1
La	NM	7.5	2.1	1.8		3.4	5.6	**53.8**	25.2	7.5
Lu	NM	NM		1.3	1.5	1.0	4.5	7.1	**58.6**	26.8
Nd	NM		1.8	2.5		3.1	5.5	**59.3**	27.6	
Pr	NM	9.7	1.8	1.5	2.2	8.3	11.8	**47.9**	19.7	9.7
Y	NM	NM	1.9	3.9	4.6	1.3	2.7	3.5	**15.4**	6.3
Yb	NM	NM	2.8	1.2	1.4	1.1	4.3	6.6	**56.6**	26.1
^{227}Th	**92.3**							2.7	3.6	
^{225}Ac					1.4		4.9	13.8	**68.7**	8.7

Elemental Analysis Methods

Table S2: Used for analysis in study 1&2: 30+ element method: Wavelengths, and quantification limits in ppm for the elements detected by the analysis method. 0.15 ppm Sc was used as an internal standard.

wavelength	Element	LOQ	wavelength	Element	LOQ	wavelength	Element	LOQ
338.289	Ag	0.005	209.426	Ge	0.05	181.975	S	0.05
396.153	Al	0.1	230.606	In	0.01	196.026	Se	0.05
188.979	As	0.05	766.49	K	0.25	407.771	Sr	0.005
249.677	B	0.1	670.784	Li	0.001	226.23	Ta	0.1
233.527	Ba	0.005	285.213	Mg	0.01	283.73	Th	0.25
313.107	Be	0.001	257.61	Mn	0.001	334.94	Ti	0.005
223.061	Bi	0.01	202.031	Mo	0.005	190.801	Tl	0.05
214.437	Cd	0.005	232.001	Ni	0.01	385.958	U	0.01
228.616	Co	0.05	309.418	Nb	0.05	290.88	V	0.005
205.561	Cr	0.01	220.353	Pb	0.05	207.912	W	0.005
324.752	Cu	0.01	780.023	Rb	0.05	213.857	Zn	0.1
238.204	Fe	0.05	197.248	Re	0.005	343.823	Zr	0.001
294.363	Ga	0.01	240.272	Ru	0.01			

Table S3: Used for analysis in study 1& 2: Lanthanide method: Wavelengths, and quantification limits in ppm for the analysis method of lanthanide elements. 0.15 ppm In was used as an internal standard.

wavelength	Element	LOQ	wavelength	Element	LOQ	wavelength	Element	LOQ
413.764	Ce	0.005	408.672	La	0.005	359.26	Sm	0.005
353.17	Dy	0.005	261.542	Lu	0.005	350.917	Tb	0.005
337.271	Er	0.005	406.109	Nd	0.010	283.73	Th	0.100
381.967	Eu	0.005	390.844	Pr	0.005	313.126	Tm	0.010
342.247	Gd	0.005	343.489	Rh	0.050	371.029	Y	0.005
345.6	Ho	0.005	361.383	Sc	0.005	328.937	Yb	0.005

Table S4: Used for analysis in study 3: Periodic Table mix 1: Wavelengths, and quantification limits in ppm for the elements detected by the analysis method. 0.15 ppm Sc was used as an internal standard.

wavelength	Element	LOQ	wavelength	Element	LOQ	wavelength	Element	LOQ
328.068	Ag	0.005	236.380	Co	0.01	232.003	Ni	0.005
394.401	Al	0.05	205.560	Cr	0.005	220.353	Pb	0.05
188.979	As	0.05	327.393	Cu	0.005	780.023	Rb	0.1
249.772	B	0.1	234.349	Fe	0.01	203.985	Se	0.05
233.527	Ba	0.005	417.206	Ga	0.01	421.552	Sr	0.001
313.107	Be	0.001	325.609	In	0.05	351.924	Tl	0.05
190.171	Bi	0.05	670.784	Li	0.1	270.093	V	0.01
393.366	Ca	0.1	280.271	Mg	0.05	202.548	Zn	0.05
226.502	Cd	0.001	259.372	Mn	0.005			

Table S5: Used for analysis in study 3: Periodic Table mix 3: Wavelengths, and quantification limits in ppm for the analysis method of lanthanide elements. 1 ppm In was used as an internal standard.

wavelength	Element	LOQ	wavelength	Element	LOQ	wavelength	Element	LOQ
413.764	Ce	0.005	408.672	La	0.005	350.917	Tb	0.005
353.17	Dy	0.005	261.542	Lu	0.005	283.73	Th	0.1
337.271	Er	0.005	406.109	Nd	0.01	313.126	Tm	0.005
381.967	Eu	0.005	390.844	Pr	0.01	371.029	Y	0.005
342.247	Gd	0.005	357.253	Sc	0.05	328.937	Yb	0.005
345.6	Ho	0.005	359.26	Sm	0.005			
